# Hematopoietic aging promotes cancer by fueling IL-1α-driven emergency myelopoiesis

**DOI:** 10.1126/science.adn0327

**Published:** 2024-10-25

**Authors:** Matthew D. Park, Jessica Le Berichel, Pauline Hamon, C. Matthias Wilk, Meriem Belabed, Nader Yatim, Alexis Saffon, Jesse Boumelha, Chiara Falcomatà, Alexander Tepper, Samarth Hegde, Raphaël Mattiuz, Brian Y. Soong, Nelson M. LaMarche, Frederika Rentzeperis, Leanna Troncoso, Laszlo Halasz, Clotilde Hennequin, Theodore Chin, Earnest P. Chen, Amanda M. Reid, Matthew Su, Ashley Reid Cahn, Laura L. Koekkoek, Nicholas Venturini, Shira Wood-isenberg, Darwin D’souza, Rachel Chen, Travis Dawson, Kai Nie, Zhihong Chen, Seunghee Kim-Schulze, Maria Casanova-Acebes, Filip K. Swirski, Julian Downward, Nicolas Vabret, Brian D. Brown, Thomas U. Marron, Miriam Merad

**Affiliations:** 1 Department of Immunology and Immunotherapy, Icahn School of Medicine at Mount Sinai; New York, NY 10029, USA; 2 Marc and Jennifer Lipschultz Precision Immunology Institute, Icahn School of Medicine at Mount Sinai; New York, NY 10029, USA; 3 The Tisch Cancer Institute, Icahn School of Medicine at Mount Sinai; New York, NY 10029, USA; 4 Division of Hematology/Oncology, Icahn School of Medicine at Mount Sinai; New York, NY 10029, USA; 5 Center for Thoracic Oncology, Icahn School of Medicine at Mount Sinai; New York, NY 10029, USA; 6 Icahn Genomics Institute, Icahn School of Medicine at Mount Sinai; New York, NY 10029, USA; 7 Oncogene Biology Laboratory, Francis Crick Institute; London, UK; 8 Lung Cancer Group, Division of Molecular Pathology, Institute of Cancer Research; London, UK; 9 Brain and Body Research Center, Icahn School of Medicine at Mount Sinai; New York, NY 10029, USA; 10 Cardiovascular Research Institute, Icahn School of Medicine at Mount Sinai; New York, NY 10029, USA; 11 Human Immune Monitoring Center, Icahn School of Medicine at Mount Sinai; New York, NY 10029, USA; 12 INSERM U932, Immunity and Cancer, Institut Curie, Paris-Cité University; Paris, France

## Abstract

Age is a major risk factor for cancer, but how aging impacts tumor control remains unclear. Here, we establish that aging of the immune system, regardless of the age of the stroma and tumor, drives lung cancer progression. Hematopoietic aging enhances emergency myelopoiesis, resulting in the local accumulation of myeloid progenitor-like cells in lung tumors. These cells are a major source of IL-1α that drives the enhanced myeloid response. The age-associated decline of DNMT3A enhances IL-1α production, and disrupting IL-1R1 signaling early during tumor development normalized myelopoiesis and slowed the growth of lung, colonic, and pancreatic tumors. In human tumors, we identified an enrichment for IL-1α-expressing monocyte-derived macrophages linked to age, poorer survival, and recurrence, unraveling how aging promotes cancer and offering actionable therapeutic strategies. (125 words)

Aging is one of the most important overall risk factors for cancer. Individuals over the age of 65 bear more than half of the annual cancer burden in the United States, and projections estimate even higher proportions over the next three decades (1). This association between aging and tumorigenesis has often been ascribed to cumulative mutational burden, repeated exposure to environmental triggers (e.g., smoking), metabolic syndromes, progressive T cell dysfunction, and the aging-driven increase in systemic inflammation (inflammaging) (2–8). While these studies have offered insights into the link between aging and the increased incidence of cancer, their translation into actionable therapies for cancer prevention or treatment has not seen widespread success. The lack of an unbiased inquiry into the cellular basis for the association between aging, inflammation, and tumorigenesis has left the study of cancer incomplete. Filling this gap in knowledge could help characterize the early stages of tumor development and thusly aid the design of screening platforms and cancer prevention therapeutics. As non-small cell lung cancer (NSCLC) is strongly associated with aging and is the most common cause of cancer-related mortality (9–12), we sought to elucidate the reasons underlying the effect of aging on the host response to NSCLC development and outcome.

We combined heterochronic bone marrow transplantation studies with an orthotopic model of primary lung adenocarcinoma (13) and single-cell RNA sequencing (scRNAseq) to complete an unbiased and detailed dissection of the tumor microenvironment (TME) in young and old mice. We identified locally-sourced IL-1α from myeloid progenitor-like cells as a critical driver of the aging-enhanced emergency myelopoiesis response that promotes immunosuppression. Blocking this axis with an anti-IL-1α antibody early during tumor initiation not only slowed lung tumor growth but also normalized emergency myelopoiesis in old mice. Blocking IL-1β also helped to reduce tumor progression, but combining IL-1α/IL-1β blockade or targeting them both using the IL-1R1 antagonist anakinra most effectively disrupted tumor initiation. In addition, we determined that hematopoietic aging enhances IL-1α and IL-1β production via a downregulation of DNA methyltransferase 3A (DNMT3A). We also examined both primary tissue and blood specimens from patients to show that these mechanisms are conserved in human myeloid cells and supports the translational relevance to human NSCLC. Altogether, our study establishes that the relationship between aging and the host response to cancer is immunologically-driven and that targeting IL-1α-driven emergency myelopoiesis may be a potential immuno-therapeutic strategy.

## Results

### The aging lungs undergo an attrition of alveolar macrophages, despite enhanced myelopoiesis

**Fig. 1:**
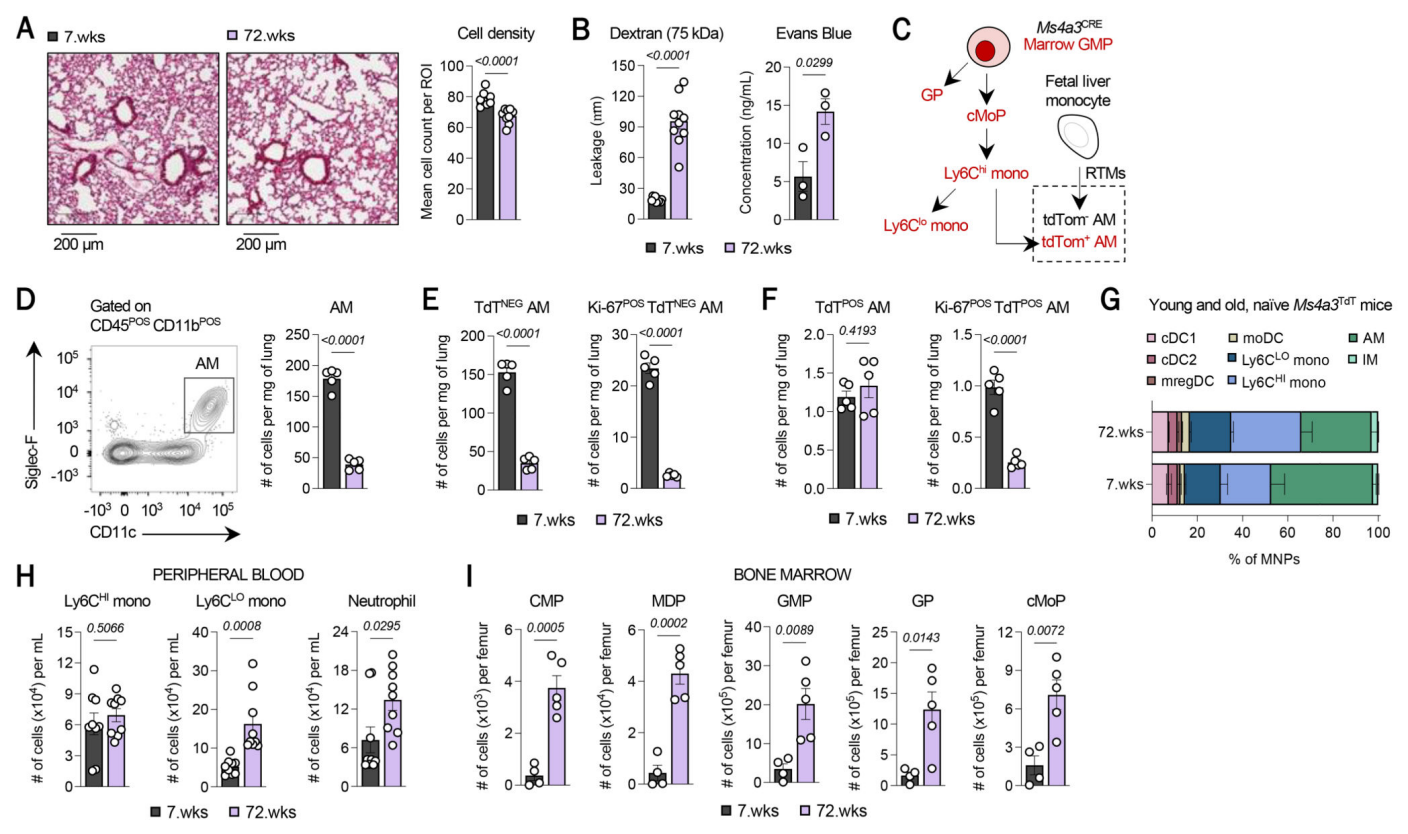
Age is associated with an attrition of tissue-resident alveolar macrophages, despite increased myelopoietic potential. (**A**) Hematoxylin and eosin staining of lung sections derived from lungs of naïve young (7-week-old, 7.wks) and old (72-week-old, 72.wks) mice (left) and quantification of cell density (right). (**B**) Vessel leakage assessed by retro-orbital administration of either Evans Blue (left) or fluorophore-conjugated Dextran (right). (**C**) Schematic of fate-mapped GMP-derived cells produced during adult hematopoiesis using the *Ms4a3*^CRE^-tdTomato (TdT) mouse. (**D**) Absolute number of alveolar macrophages (AM) in the lungs of young and old *Ms4a3*^CRE^-TdT mice at steady-state. Quantification of (**E**) TdT^NEG^ and self-renewing (Ki67^POS^) TdT^NEG^ AM and of (**F**) TdT^POS^ AM and self-renewing TdT^POS^ AM in the lungs of young and old *Ms4a3*^CRE^-TdT mice at steady-state. (**G**) Frequency distribution of major myeloid cell types identified from scRNAseq of TdT^NEG^ and TdT^POS^ myeloid cells in naïve lungs of young and old *Ms4a3*^CRE^-TdT mice. (**H**) Absolute number of Ly6C^HI^ and Ly6C^LO^ monocytes and neutrophils in the blood of naïve young and old mice. (**I**) Absolute number of CMP, MDP, GMP, GP, and cMoP in the bone marrow of naïve young and old mice. Across all experiments, n=4-5 mice were used per group. Data shown in panels (**A**), (**B**), and (**G**) are each representative of one independent experiment; panels (**D**)-(**F**), (**H**), and (**I**) are each representative of two independent experiments. Across all panels, data represent mean ± SEM. *P-*values were computed by unpaired *t*-test. (AM, alveolar macrophage; cDC1, type 1 conventional dendritic cell; cDC2, type 2 conventional dendritic cell; mregDC, mature DCs enriched in immunoregulatory molecules; moDC, monocyte-derived DC; IM, interstitial macrophage; CMP, common myeloid progenitor; MDP, monocyte-dendritic cell progenitor; GMP, granulocyte-monocyte progenitor; GP, granulocyte progenitor; cMoP, common monocyte progenitor)

To understand the effect of aging on lung cancer development and progression, we first sought to define the steady-state differences that exist between the naïve lungs of young and old mice. We used 7- and 72-week-old mice to meet this objective. The lungs of 72-week-old mice exhibited a major reduction in alveolar cell density (Fig. 1A) and increased vascular leakiness into the lung parenchyma (Fig. 1B), compared to the lungs of 7-week-old mice. We then investigated additional indicators of pulmonary aging (9) and measured cells involved in immunosurveillance. We found that old lungs harbor significantly fewer dendritic cells (DCs) and effector lymphocytes, including NK cells in the lungs and both CD8 and CD4 T cells in the blood (fig. S1A-S1C). Notably, amongst DCs, we documented a decline of type 1 conventional DCs (cDC1), but not of cDC2; rather, monocyte-derived DCs (moDC) were enriched in the lungs of old mice (fig. S1D).

As the preservation of lung function is dependent on tissue-resident alveolar macrophages (AMs) (14), we next chose to study how they are impacted by aging. As their function can be influenced by their developmental origin (i.e., fetal-derived or bone marrow monocyte-derived) (14–16), we used *Ms4a3*^CRE^ reporter (*Ms4a3*^TdT^) mice to distinguish tdTomato-expressing (TdT^POS^) AMs – derived from granulocyte-monocyte progenitors (GMPs) in the bone marrow – from TdT^NEG^ or fetal-derived/embryonic AMs (17) (Fig. 1C). This comparison enabled us to assess how aging affects the ontogenic composition of AMs. By flow cytometry, we first observed a significant decline in the absolute number of AMs (Fig. 1D), but not of other GMP-derived myeloid cells in old lungs; while neutrophils were enriched in old lungs, no significant changes were seen among monocytes and interstitial macrophages (IMs) (fig. S1, E and F). This major decline in AMs was largely due to the loss of embryonic AMs but no major changes in the number of adult monocyte-derived AMs (Fig. 1, E and F), in line with similar analyses of tissue-resident macrophages in other aging tissues (18). The concomitant attrition of cycling (Ki-67^POS^) AMs in old mice, suggested a reduced capacity for self-renewal among these tissue-resident phagocytes (Fig. 1, E-G). Taken together, our data indicated that AMs are less able to self-renew and that steady-state myelopoiesis during aging is unable to repopulate the AMs lost to time.

To further contextualize this observation, we quantified the hematopoietic stem and progenitor cells (HSPC) and mature myeloid cells (monocytes and neutrophils) in the blood and bone marrow of young and old mice. In the blood, the number of Ly6C^HI^ monocytes changed only modestly, which aligned with recent studies of circulating classical monocytes in young and old healthy donors (19–21). Instead, there were significantly more Ly6C^LO^ monocytes and neutrophils in the blood of old mice (Fig. 1H), consistent with other human studies (22–24). The bulk of these circulating monocytes that accumulated in the blood with age were TdT^NEG^ in old *Ms4a3*^TdT^ mice, suggesting that they were largely derived from monocyte-dendritic cell progenitors (MDPs) (fig. S1, G and H) (25). In bone marrow of old mice, we found an expansion of hematopoietic stem cells (HSCs) and myeloid-committed multipotent progenitors (MPP2, MPP3) and a major enrichment for the common myeloid progenitors (CMPs), MDPs, GMPs, granulocyte progenitors (GPs), and common monocyte progenitors (cMoPs) at the expense of lymphoid-committed progenitors (MPP4 and common lymphoid progenitors [CLP]) (fig. S1I and Fig. 1I). Neutrophils and monocytes were also significantly enriched in the bone marrow of old mice (fig. S1J). These data were consistent with existing knowledge on the myeloid bias of hematopoiesis at the expense of lymphopoiesis with age (26–28). Altogether, these findings indicated that though steady-state aging generates a larger reservoir of myeloid progenitors and differentiated myeloid cells in bone marrow that may be poised to respond to recruitment cues, there is still a major attrition of AMs in the aging lungs.

### Lung cancer progression is enhanced in old mice

**Fig. 2:**
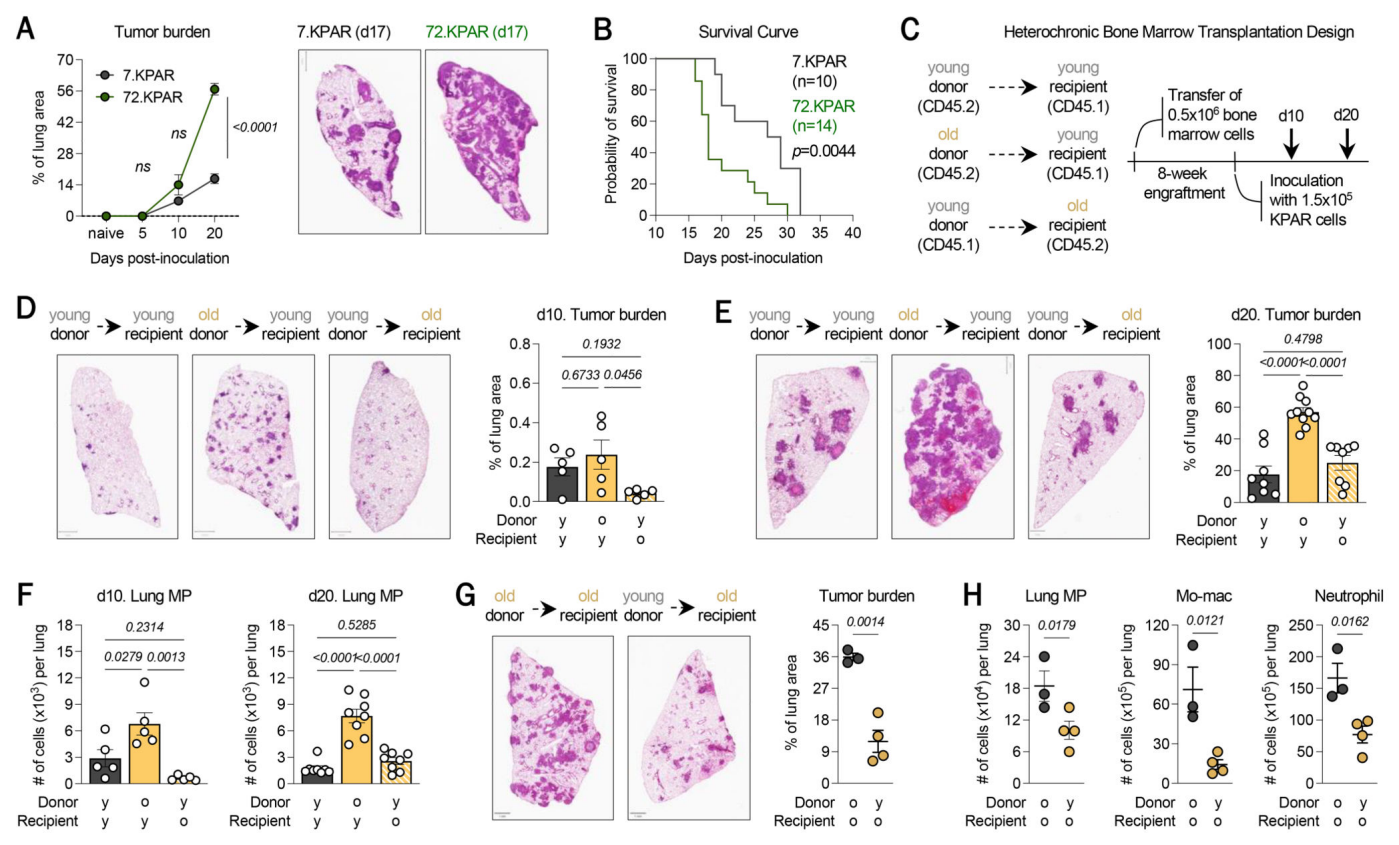
Aging of the hematopoietic compartment promotes lung cancer progression. An orthotopic model for primary lung adenocarcinoma involving the Intravenous injection of *Kras*^G12D/+^ *Tp53*^-/-^ *Rosa26*^*A3Bi-*^ *Rag1*^-/-^ (KPAR) cells was used to assess tumor growth in young (7-week-old, 7.KPAR) and old (72-week-old, 72.KPAR) mice. (**A**) (Left) Longitudinal kinetic analysis of tumor burden in the lungs of young and old mice at 5-, 10- and 20-days post-tumor cell inoculation, and (Right) tumor burden in the lungs of young and old mice at 17 days post-inoculation (n=3-5 mice per group) with representative H&E cross-sections shown at left. Scale bar = 1 mm. (**B**) Survival curve of tumor-bearing young (n=10) and old (n=14) mice. *P*-value was computed using the Log-rank Mantel-Cox test. (**C**) Experimental design of heterochronic bone marrow transplantation, involving the transfer of (1) donor bone marrow from young CD45.2 mice into young CD45.1 recipient mice, (2) donor bone marrow from old CD45.2 mice into young CD45.1 recipient mice, and (3) donor bone marrow from young CD45.1 mice into old CD45.2 recipient mice. Chimeric mice were inoculated with tumor cells after an eight-week engraftment period. (**D**) Tumor burden in the lungs of chimeric mice at 10 days post-tumor cell inoculation. Scale bar = 1mm. (**E**) Tumor burden in the lungs of chimeric mice at 20 days post-inoculation. Scale bar = 1mm. (**F**) Absolute number of lung MP in the lungs of chimeric mice at 10 (left) and 20 (right) days post-inoculation. (**G**) Tumor burden in the lungs of (black) old recipients of old donor bone marrow and of (yellow) old recipients of young donor bone marrow at 17 days post-inoculation. (**H**) Absolute number of lung MP, mo-macs, and neutrophils in the tumor-bearing lungs of chimeric mice shown in (**G**). Across all experiments, except in panel **(B**), n=3-5 mice were used per group. Data shown in panels (**A**), (**D**), (**G**), and (**H**) are from one experiment. Data shown in panels (**B**), (**E**), and (**F**) are each representative of two independent experiments. Across all panels, data represent mean ± SEM. *P-*values were computed by either unpaired *t*-test or a one-way ANOVA. (Lung MP, lung myeloid progenitor-like cell; mo-mac, monocyte-derived macrophage)

As a corollary to this, we postulated that when there is inflammation during tumorigenesis, old mice may mount a more magnified emergency myelopoiesis response, compared to young mice, and suffer from the greater burden of myeloid cells in the TME (29). In order to address this hypothesis, we first asked how aging shapes the TME and influences lung tumor growth. We used a model of NSCLC based on the transplantation and orthotopic growth of *Kras*^G12D/+^ *Trp53*^-/-^ *Rosa26*^*A3Bi*^ *Rag1*^*-/-*^ (KPAR) cells in the lungs (13). This model enabled us to specifically assess the effect of aging of host mice on tumor growth. No major differences were observed during the early stage of seeding and initiation, based on the presence of GFP-expressing tumor cells in the lung parenchyma of young (7.KPAR) and old (72.KPAR) mice at 1 day post-inoculation (fig. S2A). During the later stages of progression, however, old mice exhibited significantly greater tumor burden, compared to young mice (Fig. 2A). This was accompanied by reduced frequencies of both cytotoxic CD8 T cells and activated NK cells in lung tumors of old mice (fig. S2B). In contrast, among myeloid cells, neutrophils and monocytes were significantly enriched in lung tumors of old mice (fig. S2C). In further profiling the monocyte-macrophage lineage, we observed a steady-state decline in AMs in the lungs of both young and old mice during tumor growth (fig. S2D, left) (30–32). In their place, monocyte-derived macrophages (mo-macs), which we had shown impair tumoricidal NK cells (33), significantly accumulated more significantly in old mice than in young mice (fig. S2D, middle). These changes were associated with a shorter survival time for old mice than for young mice (Fig. 2B).

In the lungs, we also identified many myeloid progenitor-like cells (lung MPs) defined as CD45^POS^ Lin^NEG^ [CD11b, Ly6G, CD3, B220, Ter-119, NK1.1] Sca-1^NEG^ c-Kit^POS^ CD135^NEG^ FcɣRII/III^POS^ CD34^POS^ cells in lung tumors of young and old mice (fig. S3A) (34–39). Lung MPs also highly expressed both Ly6C and CD115, suggesting that they also share a monocytic phenotype with *bona fide* cMoPs found in bone marrow (fig. S3B). Intravenous labeling of circulating CD45^POS^ cells enabled us to distinguish extravascular from intravascular immune cells in the lungs (40, 41), and this showed that lung MPs remain largely unlabeled (i.e., largely reside in the lung parenchyma) and are a true local population (fig. S3C). In old, tumor-bearing *Ms4a3*^TdT^ mice, we confirmed that lung MPs are derived from adult myelopoiesis during tumor growth (fig. S3D) and are able to proliferate (fig. S3E).

Lung MPs exhibited the most dramatic expansion in old mice, compared to young mice, and as early as 10 days post-inoculation with tumor cells, before a major difference in tumor load could be discerned between young and old mice (fig. S2D, right). Moreover, while we could appreciate a significant expansion of GMPs in bone marrow of old mice either in the presence or absence of tumor cues, the substantial accumulation of lung MPs observed in the lungs of old mice was driven by tumor cues only (fig. S3, F and G). In sum, these findings pointed to an exacerbated emergency myelopoiesis response that associates with worse tumor outcome in old mice.

### Aging of the immune system is a major driver of lung cancer progression

Whether these immunological differences were alone responsible for the age-dependent progression of lung cancer remained uncertain. Prior studies had already reported on the pro-tumorigenic effects of an aged stromal compartment (42–44), so we designed a heterochronic bone marrow transplant (BMT) to parse out whether aging of the immune and/or non-immune ecosystems of the lung TME are responsible for the effect of host aging on lung cancer growth (Fig. 2C). Briefly, donor bone marrow from young or old mice were transferred to either young or old recipient mice; this approach was justified by prior work that demonstrated that HSCs are resistant to reprogramming by an age-mismatched bone marrow niche (45–47). After an eight-week engraftment period, mice were inoculated with KPAR cells, and tumor burden was evaluated at 10 and 20 days post-inoculation (Fig. 2C). Strikingly, as early as 10 days, young recipients of old bone marrow exhibited the greatest tumor burden (Fig. 2D), and this was statistically significant at day 20 (Fig. 2E). Importantly, old recipients of young bone marrow bore a tumor load that was comparable to that of young mice reconstituted with young bone marrow (Fig. 2, D and E), suggesting that aging of immune cells significantly impacts lung tumor growth.

In accordance with the enhanced tumor growth seen in the lungs of young mice reconstituted with old bone marrow, there were more proliferative regulatory T cells (Tregs), fewer cytotoxic CD8 T cells, and significantly fewer activated NK cells in these mice, compared to mice reconstituted with young bone marrow (fig. S4, A and B). Early during tumor formation, AMs were more abundant in the lungs of young recipients, compared to old recipients, likely reflecting the steady-state presence of AMs that had not yet been perturbed by nascent micro-lesions (fig. S4C, left). However, the frequency of mo-macs was already significantly higher in the lungs of mice with old bone marrow, relative to either group of recipients of young bone marrow (fig. S4D, left), indicative of an early and robust emergency myelopoiesis response in carriers of an old immune system. At the later timepoint, once tumors were established, the frequency of AMs was significantly reduced in the lungs of mice with old marrow, as the tumor burden was the greatest (fig. S4C, right). By contrast, mo-macs had accumulated in the lungs of all mice, but this expansion was most pronounced in the young recipients of old bone marrow (fig. S4D, right), consistent with the inferior NK cell response in these mice. Finally, we observed that lung MPs were significantly enriched in mice reconstituted with old bone marrow as early as 10 days post-inoculation, before the age-dependent difference in tumor burden could even be realized with sufficient statistical power (Fig. 2F).

To demonstrate whether reconstitution with a youthful immune system rescues the anti-tumor response to the age-associated growth of lung tumors in old mice, we compared old recipients of either young or old bone marrow. In this setting, we found that old mice with young bone marrow exhibited a significantly lower tumor load than their counterparts with old marrow (Fig. 2G). In accordance with this measurement, we documented a reduced number of the lung MPs, mo-macs, and neutrophils in the tumor-bearing lungs of old mice with young bone marrow (Fig. 2H). Altogether, these data established that hematopoietic aging is sufficient to promote lung cancer progression – independently of the aging of the non-immune stroma – by augmenting tumor-induced emergency myelopoiesis.

### An IL-1α signature defines myeloid progenitor-like cells in lung tumors of old mice

These findings prompted us to further characterize the immune cells within the lung TME of young and old mice. To that end, we performed scRNAseq on 33,572 CD45^POS^ cells sorted from the tumor-bearing lungs of young and old mice. Unsupervised clustering segregated contaminating non-immune cells, and this yielded the first comparative single-cell atlas of the lung TME in young and old mice. This included T cells, NK cells, B cells, and myeloid cells. The tumor-bearing lungs of old mice harbored reduced frequencies of stem-like CD4 T cells and activated NK cells, compared to young tumor-bearing mice (fig. S5A-S5C), whereas mo-macs were notably enriched in lung tumors of old mice (fig. S5, D and E). To determine the relevance of their enrichment, we challenged *Trem2* knockout mice with tumor cells, since these tumor-associated mo-macs broadly expressed *Trem2* (fig. S5D). In alignment with our prior study of TREM2 (31), we found a drastically reduced tumor load in these mice, compared to their wild-type counterparts (fig. S5F). As TREM2 is not expressed by non-immune cells of the lung TME of either mice (fig. S5G, left) or humans (fig. S5G, right), these data supported the notion that myeloid cells contribute significantly to the enhanced growth of lung cancer in old mice.

**Fig. 3:**
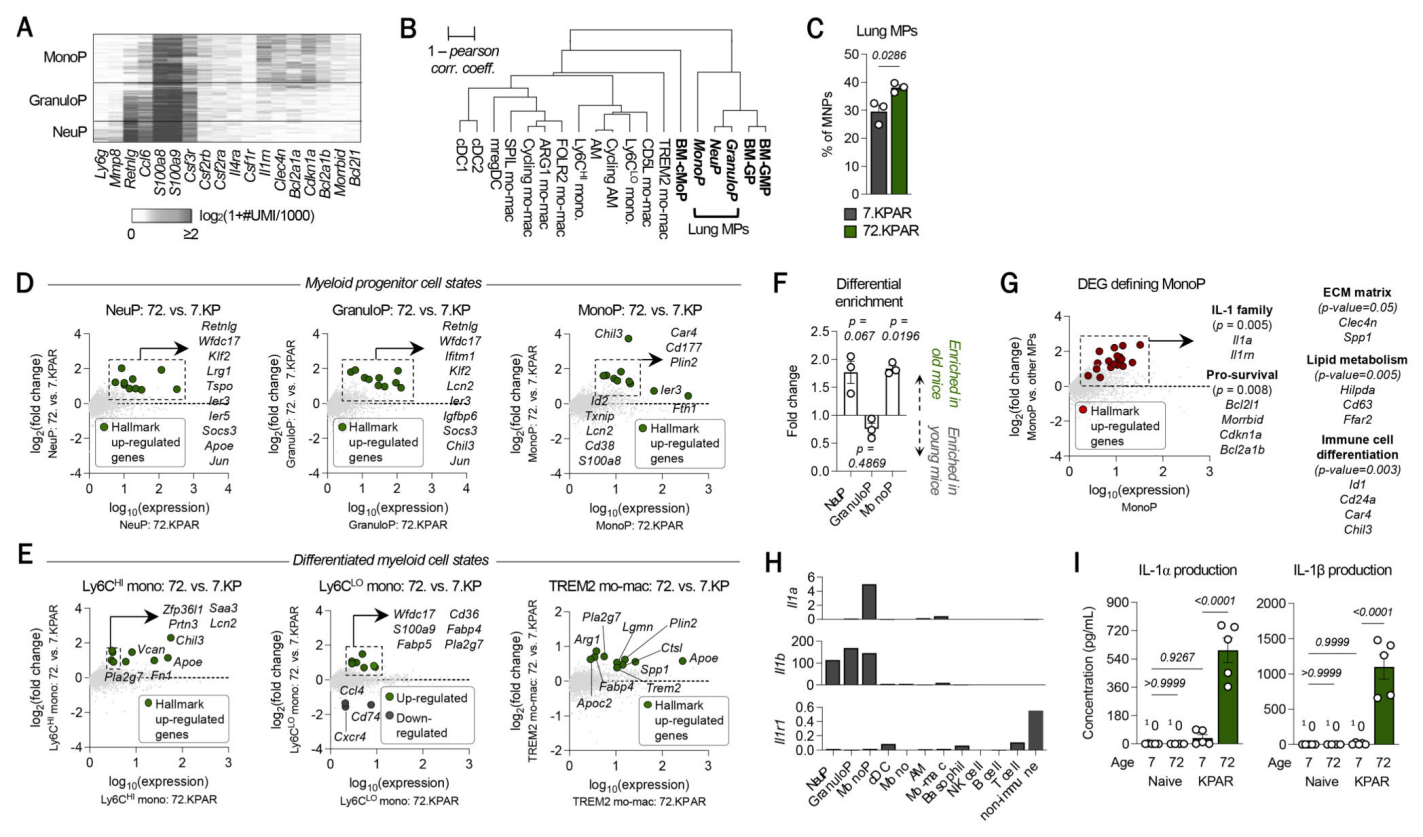
An IL-1α signature defines myeloid progenitor-like cells born from age-enhanced emergency monopoiesis. CD45^POS^ cells were sorted from young (n=3) and old (n=3) tumor-bearing mice and sequenced at the single-cell level. (**A**) Sub-clustering of myeloid progenitors (MPs) using a *K*-nn graph partitioning approach and annotation of MP cell states (i.e., MonoP, GranuloP, NeuP) based on defining markers and shown through a heatmap plotting UMI counts per cell. (**B**) Transcriptional similitude amongst myeloid cells was determined by hierarchical clustering based on mRNA expression profile. Transcriptomes of bone marrow progenitors (BM-GMP, BM-GP, BM-cMoP) in the tumor-bearing setting were used. (**C**) Frequency of total lung MPs in tumor-bearing lungs of young and old mice. (**D**) Differentially expressed genes (DEGs) between (top) NeuP, (middle) GranuloP, and (bottom) MonoP from lung tumors of old vs. young mice. (**E**) DEGs between (left) Ly6C^HI^ monocytes, (middle) Ly6C^LO^ monocytes, and (right) TREM2 monocyte-derived macrophages (mo-macs) from lung tumors of old vs. young mice (**F**) Fold change in the frequency of NeuP, GranuloP, and MonoP, relative to the mean frequency of each respective cell state in lung tumors from young mice. (**G**) DEGs that define MonoP, based on Wilcoxon Rank Sum testing of MonoP vs. all other MPs. Hallmark genes plotted in red. Significant gene networks identified by gene ontology analysis. (**H**) Mean UMI of *Il1a, Il1b*, and *Il1r1* across immune cells. (**I**) Concentration of IL-1α (left) and IL-1β (right) in the lung homogenate of digested naïve and tumor-bearing lungs of young and old mice. In panel (**I**), n=5 mice were used per group. Data shown in (**A**)-(**G**) are from one independent experiment. Raw data from (**B**) are taken from Caiado et al., 2023. Data represent mean ± SEM. *P*-values were computed by unpaired *t-*test. (MonoP, monocytic progenitor; GranuloP, granulocytic progenitor; NeuP, neutrophilic progenitor; GMP, granulocyte-monocyte progenitor; GP, granulocyte progenitor; cMoP, common monocyte progenitor)

By scRNAseq, we also identified the local population of lung MPs, based on their co-expression of *Csf1r, Csf2ra, Csf3r, Cebpe*, and *Klf4* and their lack of expression of markers defining differentiated myeloid cells. The lung MPs included neutrophilic (NeuP; *Ly6g*), granulocytic (GranuloP; *Mmp8, Ccl6, S100a8, S100a9*) (48), and monocytic progenitors (MonoP; *Clec4n, Csf1r, Bhlhe40*) (49–51) (Fig. 3A). To contextualize their relationship to their bone marrow counterparts, we compared the transcriptome of the lung MPs with those of GMPs, GPs, and cMoPs from bone marrow of tumor-bearing mice (29). Hierarchical clustering showed that NeuP, GranuloP, and MonoP exhibit the greatest transcriptional similitude with their bone marrow progenitors, relative to the differentiated myeloid cells of the lung TME (Fig. 3B), suggesting that lung MPs likely represent myeloid progenitor-like cells upstream of differentiated monocytes and mo-macs. Importantly, in line with their quantification via flow cytometric methods, we found a significant enrichment of cells expressing the lung MPs’ mRNA program in lung tumors of old mice (Fig. 3C).

We next asked whether comparing lung MPs from lung tumors of young mice with those of old mice could elucidate molecular differences in myelopoiesis due to aging. Among DEGs, genes upregulated in old NeuP, GranuloP, and MonoP included *Ier3, Wfdc21, Txnip*, and *Socs3*, among others (Fig. 3D, Table S1). As the expression of these markers is typically higher in precursors upstream of lung MPs – such as the hematopoietic stem cells (HSCs), multipotent progenitors (MPPs), and the GMPs (Table S2) – these DEGs suggested that lung MPs in tumors of old mice are less mature than those of young mice. Extending this analysis to differentiated myeloid cells, we found that Ly6C^HI^ monocytes in old mice, for instance, express higher levels of *Prtn3, Lcn2*, and *Chil3*, compared to those in young mice (Fig. 3E, left; Table S1); as these markers are highly expressed in bone marrow myeloid progenitors and monocytes, these DEGs also indicated that old Ly6C^HI^ monocytes in lung tumors are transcriptionally less mature than their counterparts in young mice. In comparing the broad collection of TREM2 mo-macs in old mice with those in young mice, we found that old mo-macs expressed higher levels of genes co-regulated with TREM2 (31) (Fig. 3E, right). In sum, the relative abundance of myeloid progenitor-like cells and immature myeloid cells in old mice, compared to their corresponding counterparts in young mice, supported our hypothesis that tumor-induced emergency myelopoiesis is worsened with age.

Among the lung MPs, MonoP were significantly enriched in lung tumors of old mice (Fig. 3F). As our phenotypic profiling had also indicated that the bulk of lung MPs are monocytic (fig. S3B), this narrowed our study’s focus to MonoP. Compared to the other lung MPs, MonoP expressed significantly higher levels of mRNA encoding *Il1a* and *Il1rn* and of those encoding pro-survival molecules like BCL-xL (*Bcl2l1*), a regulator of myeloid cell life-span (*Morrbid*) (52, 53), the negative regulator of cell cycle and senescence marker p21 (*Cdkn1a*), and other BCL-2 family members (*Bcl2a1b, Bcl2a1d*) (Fig. 3G, Table S3). The transcription of *Il1a* (encoding the alarmin IL-1α) by MonoP was quite striking to us, as it was highly specific to these cells, whereas *Il1b* (encoding the pro-form cytokine IL-1β) was ubiquitously expressed by all lung MPs and mo-macs (Fig. 3H). Notably, the expression of IL-1 receptor 1 (IL-1R1, encoded by *Il1r1*), the cognate receptor for IL-1α and IL-1β, was absent from local immune cells in the TME (Fig. 3H). We confirmed that both IL-1α and IL-1β were highly upregulated at the protein level in the tumor-bearing lungs of old mice by ELISA (Fig. 3I). The high levels of these two cytokines in lung tumors of old mice further rationalized our study of the functional relevance of IL-1 in lung cancer progression in old mice.

### Blocking IL-1α:IL-1R1 signaling delays lung cancer progression

**Fig. 4:**
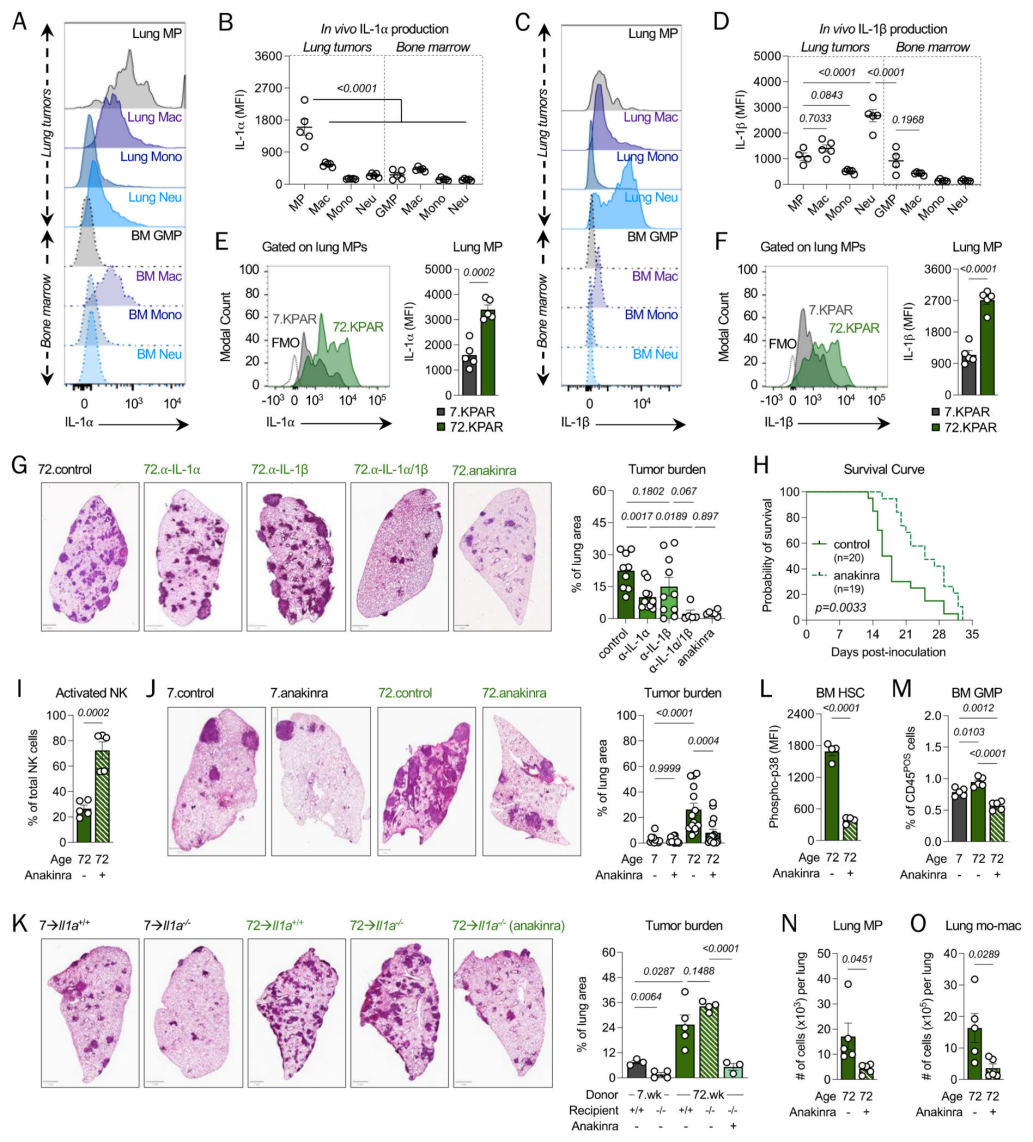
IL-1α signaling fuels aging-driven tumor growth. Brefeldin A was administered to young (n=5) and old (n=5) tumor-bearing mice to quantify *in vivo* production of IL-1α and IL-1β. (**A**), (**B**) IL-1α and (**C**), (**D**) IL-1β production levels in MPs, macrophages (macs), monocytes (mono), neutrophils (neu) from lung tumors and in GMP, macrophages, monocytes, and neutrophils from bone marrow of the same mice. Expression of (**E**) IL-1α and (**F**) IL-1β by lung MPs in young and old tumor-bearing mice. (**G**) Tumor burden in the lungs of old mice that received either isotype control, anti-IL-1α neutralizing antibody, anti-IL-1β neutralizing antibody, or anakinra after 16 days post-tumor cell inoculation. Scale bar = 1mm. (**H**) Survival curve of control (n=20) and anakinra-treated (n=19) tumor-bearing old mice. Solid line: control; Dotted line: anakinra-treated. Difference in median survival of 8 days. *P*-value was computed using the Log-rank Mantel-Cox test. (**I**) Frequency of activated NK cells in lung tumors of control (PBS) old mice and old mice that received anakinra immediately after tumor cell inoculation. (**J**) Tumor burden in the lungs of young and old mice that received either control (PBS) or anakinra after 16 days post-tumor cell inoculation. Scale bar = 1mm. (**K**) Tumor burden in chimeric mice: wild-type or *Il1a*^-/-^ recipient mice were transplanted with either young or old donor bone marrow and were either treated with control (PBS) or anakinra. Scale bar = 1mm. (**L**) Levels of phosphorylated p38 (phospho-p38) in bone marrow HSCs of old mice that either received control (PBS) or anakinra. (**M**) Frequency of bulk GMPs in the bone marrow of young and old mice that either received control (PBS) or anakinra immediately after tumor cell inoculation. (**N**) Absolute number of lung MP in lung tumors of control (PBS) old mice and old mice that received anakinra immediately after tumor cell inoculation. (**O**) Absolute number of mo-mac in lung tumors of mice shown in (**N**). Data shown in panels (**A**)-(**J**) and (**M**)-(**O**) are representative of at least two independent experiments; data in panels (**K**) and (**L**) are each from one independent experiment. Data are represented as mean ± SEM. *P*-values were computed by either one-way ANOVA or by unpaired *t-*test. (Lung MP, lung myeloid progenitor-like cell; GMP, granulocyte-monocyte progenitor; NK cell, natural killer cell; HSC, hematopoietic stem cell; mo-mac, monocyte-derived macrophage)

We first validated the transcriptomic data by measuring the production of IL-1α and IL-1β by lung MPs, relative to differentiated myeloid cells, at the protein level. Lung MPs produced the highest level of IL-1α (Fig. 4, A and B), whereas IL-1β was largely produced by neutrophils in lung tumors (Fig. 4, C and D). Strikingly, still, both IL-1α and IL-1β production by lung MPs were significantly increased in tumor-bearing lungs of old mice than in young mice (Fig. 4, E and F), suggesting that hematopoietic aging enhances their production. To determine the functional relevance of this, we then performed antibody-based blockade of IL-1α and/or IL-1β and compared these therapies with the IL-1R1 antagonist anakinra, which would interfere with IL-1α and IL-1β signaling simultaneously. As lung MPs were shown to accumulate in lung tumors early during tumor initiation, we began treatment at 24 hours post-inoculation of tumor cells. IL-1α blockade delayed lung cancer progression more robustly than IL-1β blockade (Fig. 4G); notably, though, the combination of both exhibited an apparent synergistic effect in reducing tumor growth that was comparable to treating mice with anakinra (Fig. 4G). Importantly, anakinra therapy yielded a significant benefit in survival of old mice that was linked to an enhanced tumoricidal NK cell response (Fig. 4, H and I). Comparing the effectiveness of anakinra in young and old mice, we found that IL-1R1 antagonism elicits a therapeutic effect in old mice (Fig. 4J), earlier than in young mice (fig. S6A), since an effect was observed in old mice but not in young mice at day 16 post-inoculation. A delay in lung cancer progression could be realized in young mice 22 days post-inoculation (fig. S6A). These effects still translated to improvements in survival for both young and old mice, albeit starting at different times during disease course (Fig. 4H and fig. S6B).

To understand how disrupting IL-1R1 signaling attenuates the age-enhanced growth of lung tumors, we sought to identify the cell types that express IL-1R1. We first considered direct signaling of IL-1α/β to the tumor cells themselves, as they expressed IL-1R1 (fig. S7A). Upon deleting *Il1r1* in the tumor cells, we found that the growth of these IL-1R1-deficient cells *in vivo* was comparable to IL-1R1-proficient tumor cells; in fact, aging of the host still promoted the progression of IL-1R1-deficient tumors (fig. S7B). To determine whether there might be a difference in growth fitness between IL-1R1-proficient and -deficient tumor cells, we also evaluated the simultaneous growth of IL-1R1-proficient and deficient tumors in the context of a shared TME. In brief, tumor cells either proficient or deficient in IL-1R1 were generated using CRISPR-Cas9 genomics, mixed in equivalent ratios, and injected intravenously into mice. Cyclic immunofluorescence was used to stain for protein tags that distinguished IL-1R1-proficient tumor cells from -deficient ones, based on the gRNA they received. Even in this scenario, we found no significant fitness advantage between the two genotypes (fig. S7C). This suggested that, within the local TME, the likely responders of IL-1α are the non-cancerous, immune and non-immune cells that populate the TME.

At the protein level, we found that non-immune cells are dominant expressors of IL-1R1 in the local TME and include cancer-associated fibroblasts (CAFs), epithelial cells, and endothelial cells (fig. S7D). Though our BMT studies had already highlighted the unique relevance of hematopoietic – but not stromal – aging to the age-dependent progression of lung cancer, our experiments did not yet exclude the age-independent role of the IL-1R1^POS^ non-immune cells, such as CAFs (54–56), in indirectly mediating the recruitment of immunosuppressive myeloid cells to the TME. To address this point, we transplanted either young or old bone marrow into young, age-matched *Il1r1*^+/+^ (wild-type) and *Il1r1*^-/-^ recipients, and we then challenged these mice with KPAR cells. Strikingly, while we saw a reduction in tumor burden in *Il1r1*^-/-^ mice with young marrow, compared to their WT counterparts, genetic deletion of IL-1R1 in the non-immune compartment (*Il1r1*^-/-^ recipients) of mice with an aged immune system exhibited tumor burden comparable to that in WT recipients (fig. S7E). In line with this observation, though the number of lung MPs was reduced in *Il1r1*^-/-^ recipients of old bone marrow, the number of mo-macs and Tregs was comparable between the *Il1r1*^+/+^ and *Il1r1*^-/-^ recipients of old bone marrow (fig. S7, F and G). These findings highlighted that IL-1 signaling to immune cells has a much stronger, pro-tumor effect in the presence of an aged immune system than in the presence of a youthful immune system.

To further refine this observation, we also tested whether IL-1α derived from stressed non-immune cells – given its prototypical function as an alarmin released by cell stress, damage, or senescence (57–60) – contributes to the age-enhanced progression of lung cancer. Similar to the aforementioned transplant study involving *Il1r1*^-/-^ recipients, we transferred young or old donor bone marrow into either *Il1a*^+/+^ (wild-type) or *Il1a*^-/-^ recipients. A subset of *Il1a*^-/-^ recipients that received old donor bone marrow were also treated with anakinra. We observed that *Il1a*^-/-^ mice with a young immune system exhibited significantly reduced tumor burden, compared to WT recipients, but notably, we discovered that genetically deleting IL-1α from non-immune cells does not impact lung tumor growth in the presence of an aged immune system (Fig. 4K). This suggested to us that hematopoietic aging may compensate for the lack of IL-1α derived from non-immune cells. Moreover, we found that anakinra therapy significantly delays lung cancer progression in *Il1a*^-/-^ recipient mice with old donor bone marrow (Fig. 4K), indicating that the anti-tumoral potency of anakinra is determined by the disruption of IL-1α signaling from immunological sources, which we specified is largely contributed by lung MPs in the local TME. Together, these transplant studies demonstrated that IL-1α:IL-1R1 signaling from lung MPs to hematopoietic targets is central and specific to the pro-tumorigenic effect of hematopoietic aging.

Based on these data and transcriptional profiling documenting negligible levels of *Il1r1* expression by local immune cells (Fig. 3H and fig. S7D), we explored peripheral immune cells that could respond to IL-1. So, we surveyed hematopoietic cells in bone marrow. By flow cytometry, we determined that hematopoietic progenitors of bone marrow expressed significantly higher levels of IL-1R1 than local innate and adaptive immune cells, which expressed minimal levels of IL-1R1, in the tumor-bearing lungs of old mice (fig. S7H). Even in naïve mice, we found that hematopoietic progenitors of bone marrow expressed higher levels of both IL-1R1 protein and mRNA than differentiated myeloid (i.e., neutrophils, monocytes) and lymphoid (i.e., B cells, which showed the highest levels of IL-1R1 in the local TME) cells (fig. S7I). While IL-1β is known to act directly on HSCs and promote myeloid differentiation (61–66), the impact of IL-1α on HSCs has been less studied. So, we completed our own analysis of a publicly available dataset, generated by bulk RNA sequencing of sorted HSCs that were treated with PBS or IL-1α (67). We identified a significant upregulation of major myeloid genes, including *Ms4a3, Chil3, Ly6c2, Csf2rb, Itgam*, and *Ly6g* (fig. S7J), in HSCs treated with IL-1α, which suggested that IL-1α also promotes HSC commitment to the myeloid lineage. Based on this observation, we surmised that the IL-1α produced by local MPs found in tumor lesions may fuel enhanced emergency myelopoiesis in old mice by promoting a pro-myeloid feedback loop with HSPCs in bone marrow.

To determine whether there is a normalization of emergency myelopoiesis upon blocking IL-1 signaling, we examined the bone marrow and lungs of anakinra-treated mice. We first measured phosphorylated (phospho-) p38 levels in HSCs to determine whether anakinra reduced the impact of IL-1 signaling, and other tumorigenesis-associated MAPK-activating cues, in HSCs. Phospho-p38 levels were significantly reduced in HSCs of anakinra-treated, tumor-bearing old mice (Fig. 4L), and the frequency of bone marrow GMPs was also reduced to levels comparable to that seen in young tumor-bearing mice (Fig. 4M). In the lungs, lung MPs and mo-macs were also significantly reduced in number upon anakinra therapy (Fig. 4, N and O), indicating that blocking IL-1 signaling during tumor initiation deters HSC commitment to the GMP lineage and the recruitment of GMP-derived cells to the TME. These phenotypes could also be observed in young mice but only after approximately a week later (fig. S6C). These data indicated that IL-1 signaling from lung MPs to hematopoietic progenitors in bone marrow forms a dominant axis of emergency myelopoiesis in old mice.

### The precancer setting defines the therapeutic window for optimal anakinra efficacy

To validate the importance of the time in which we administer anakinra, we tested whether delaying anakinra therapy would compromise its efficacy. We compared tumor growth in old mice that received anakinra at day 14 (4 doses) (treatment of established tumors) with both untreated controls and with mice that received anakinra based on our original regimen (starting at day 1 post-inoculation, total of 9 doses) prior to the formation of solid tumors. Postponing treatment with anakinra – which was timed to act during the exponential growth phase of lung tumors in old mice (i.e., on established tumors), according to our kinetics study – did not decrease tumor burden in old mice (fig. S8A). We also tested these conditions in aged, genetically-engineered mice that develop *Kras*^G12D/+^ *Trp53*^-/-^ lung cancer after being given an adenovirus that induces Cre recombination (68). In this setting, mice that were given anakinra to treat established tumors (starting at 18-weeks post-inoculation) had comparable tumor burden to control mice (fig. S8B); this contrasted with mice that received anakinra starting at 1-week post-inoculation, suggesting that inhibiting IL-1α signaling at the precancer stage/during tumor initiation is a unique opportunity to derail lung tumor growth.

### Age-enhanced myelopoiesis via IL-1R1 signaling promotes growth of multiple murine cancers

To broaden the scope of our study of the IL-1α/IL-1R1 axis of age-enhanced emergency myelopoiesis, we wanted to determine whether this proposed mechanism for aging-driven cancer progression is relevant to other cancer settings. Colorectal cancer (CRC) is the third most common cancer globally and is the second leading cause of cancer-related death in the United States. As risk for CRC increases exponentially with age (69), we sought to assess the relevance of our findings in this setting. We leveraged an orthotopic model of CRC, involving the intracecal transplantation of *Apc*^fl/fl^ *Kras*^LSL-G12D/+^ *p53*^fl/fl^ *Smad4*^fl/fl^ (AKPS) colon tumor organoids. We surgically implanted these tumor cells into young and old mice and administered anakinra after one week post-implantation. Strikingly, we found that AKPS growth was significantly greater in old mice, compared to in young mice (fig. S9A). Importantly, old mice treated with anakinra exhibited significantly delayed progression of AKPS tumors, relative to control old mice (fig. S9A). In the bone marrow of these mice, we observed significant age-enhanced expansion of myeloid progenitors linked to hematopoietic aging in old controls, and anakinra normalized this myeloid bias (fig. S9B). Consistent with these results, we found that anakinra also reduced the accumulation of mo-macs in AKPS tumors (fig. S9C) and helped reduce the frequency of exhausted T cells while rescuing the cytotoxic T cell response (fig. S9, D and E). Finally, we also performed the orthotopic transplantation of tumor organoids derived from *Kras*^LSL-G12D/+^; *Ptf1a*^CRE^ (KC) mice into the pancreas of young and old mice and administered anakinra after one week post-implantation. Here, we found that an old host response promotes KC tumor progression but anakinra ameliorates this effect (fig. S9F). Consistent with this phenotype, the bone marrow of anakinra-treated KC tumor-bearing old mice harbored significantly fewer HSPCs and GMPs (fig. S9, G and H), suggesting that anakinra also normalizes emergency myelopoiesis that is enhanced due to hematopoietic aging during pancreatic cancer progression. Altogether, these data suggest that the IL-1-dependent promotion of age-enhanced myelopoiesis is relevant to the age-associated anti-tumor response to other solid tumors.

### Decline of DNMT3A during hematopoietic aging enhances IL-1α production

**Fig. 5:**
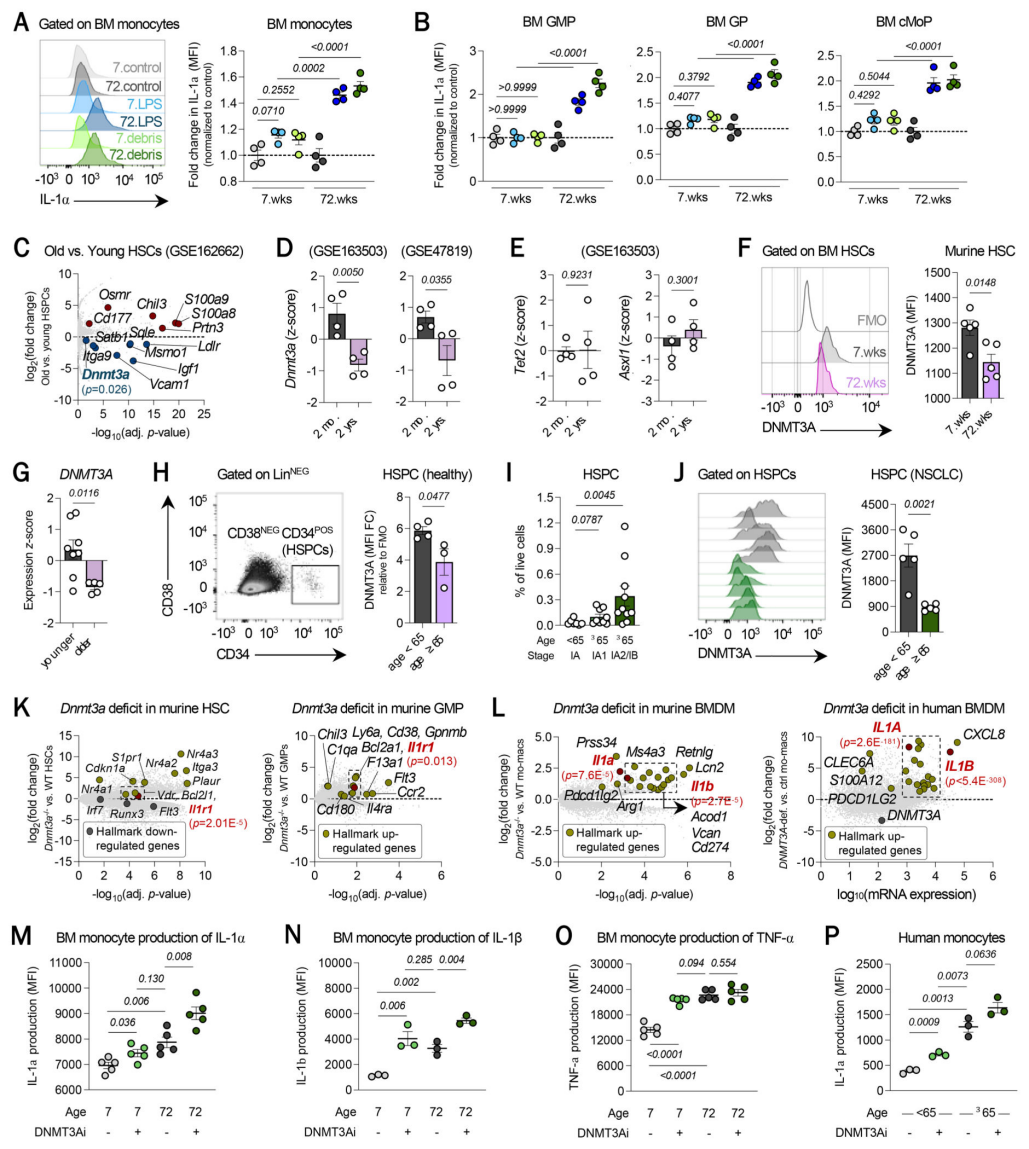
Aging of myeloid cells results in DNMT3A deficiency and promotes expression of the IL-1α program. IL-1α production by (**A**) bone marrow monocytes and (**B**) GMPs, GPs, cMoPs from young and old mice in the presence of control media, LPS, or apoptotic cell debris. (**C**) DEGs between young and old HSCs sorted from bone marrow of young and old mice. Relative expression of (**D**) *Dnmt3a* and (**E**) *Tet2* and *Asxl1* mRNA by sorted bone marrow HSCs from young and old mice. (**F**) Protein expression of DNMT3A by HSCs from bone marrow of naïve, young and old mice. (**G**) Relative expression of *DNMT3A* mRNA by HSCs from younger (n=3; ages 30, 31, 41) and older (n=3; ages 60, 60, 84) healthy donors. (**H**) Protein expression of DNMT3A by circulating HSPCs from the peripheral blood of younger (age<65) and older (age≥65) healthy donors. (**I**) Frequency of circulating HSPCs from the peripheral blood of NSCLC patients. (**J**) Protein expression of DNMT3A by circulating HSPCs from younger (age<65) and older (age≥65) NSCLC patients. (**K**) DEGs between *Dnmt3a*^-/-^ and *Dnmt3a*^*+*/+^ (WT) murine HSCs (left) and GMPs (right). (**L**) DEGs between DNMT3A-proficient and –deficient (left) murine and (right) human bone marrow monocyte-derived macrophages and blood monocyte-derived macrophages, respectively. (**M**) IL-1α, (**N**) IL-1β and (**O**) TNF-α production by LPS-stimulated young and old bone marrow monocytes that were either untreated or treated with the DNMT3A inhibitor across independent experiments. (**P**) IL-1α production by LPS-stimulated blood monocytes from younger (age<65) and older (age≥65) healthy donors that were either untreated or treated with the DNMT3A inhibitor. Data shown in (**A**), (**B**), (**M**)-(**P**) are representative of at least two independent experiments. Data shown in (**H**)-(**J**) were collected across at least three independent experiments. Raw data for panels (**C**) and (**D**) were obtained from Itokawa et al., 2022, Kovtonyuk et al., 2022, and Sun et al., 2014; for panel (**G**) were obtained from Oetjen et al., 2018 and Ainciburu et al., 2023; for panels (**K**) and (**L**) were obtained from Zhang et al., 2022 and Guryanova et al., 2016 and Rausch et al., 2023 and Cobo et al., 2022. Data are represented as mean ± SEM. *P*-values were computed by either unpaired *t*-test or one-way ANOVA.

Given that *Il1a* transcription by MPs was triggered in lung tissues (Fig. 4B), we endeavored to identify local cues that might elicit this response from the lung MPs within the TME. Among the potential candidates, we postulated that exposure to cell debris could instigate the production of IL-1α by MPs in lung tumors, given the cellular turnover of tumor cells that occurs in the local microenvironment. To test this hypothesis, we employed a reductionist *in vitro* system, in which we exposed bone marrow monocytes that transcriptionally resemble lung MPs to apoptotic cell debris and measured IL-1α production. We compared this to standard agonism of TLR4 with low-dose LPS (lipopolysaccharide). Old monocytes were more sensitive to TLR4 agonism, relative to young monocytes, and produced much higher levels of IL-1α (Fig. 5A). Importantly, we also observed that exposing these cells to apoptotic tumor cell debris elicited a much stronger IL-1α response from old monocytes than from young monocytes (Fig. 5A). This was also true for bone marrow progenitors (Fig. 5B).

These data revealed (1) that cell debris can induce the production of IL-1α and (2) that hematopoietic aging alters the cell-intrinsic phenotype of both monocytic cells and their MPs towards states that predispose them to produce more IL-1α. To further explore the latter, we analyzed bulk RNA-seq of sorted HSCs from bone marrow of young and old mice to identify transcriptional differences associated with aging (47). From this analysis, we found that old HSCs significantly downregulated the DNA methyltransferase 3A (*Dnmt3a*) (Fig. 5C), and this decline in mRNA expression could be observed in our own independent analyses of two other external datasets, as well (Fig. 5D) (70, 71). This was significant to us, as loss-of-function mutations in DNMT3A are leading drivers of clonal hematopoiesis of indeterminate potential (CHIP), a highly inflammatory condition common in the elderly, and are strongly associated with lung cancer risk (72). Other genes, such as *Tet2* and *Asxl1*, in which mutations also result in CHIP, did not exhibit a similar reduction (Fig. 5E). We confirmed this downregulation at the protein level with our own young and old mice; by flow cytometry, we observed a decrease in intranuclear DNMT3A levels in murine bone marrow HSCs with age (Fig. 5F). We also confirmed this steady-state decline in *DNMT3A* in our independent analyses of two external datasets of human HSCs that were sequenced from otherwise healthy donors (Fig. 5G) (73, 74) and in other published reports (75, 76). To further establish the relevance of these findings to human biology, we measured DNMT3A levels in circulating HSPCs from the blood of cancer-free, healthy younger and older donors (Fig. 5H) and of patients with NSCLC, from whom we documented increasing frequencies of HSPCs with age and worsening stage (Fig. 5I). In both subsets of HSPCs, we observed decreased levels of DNMT3A in those of older individuals (Fig. 5, H and J). Not only do these data indicate increasing degrees of emergency hematopoiesis with age but also confirm an age-associated decline in DNMT3A, even in cancer patients.

To characterize the effect of declining DNMT3A levels, we looked to comparisons of cells with DNMT3A mutations conferring a loss-of-function and their WT counterparts. Our independent analysis of bulk RNA-seq of *Dnmt3a*^-/-^ and *Dnmt3a*^+/+^ (wild-type, WT) HSCs (77, 78) revealed that, among many major transcriptional changes, DNMT3A deficiency results in the upregulation of *Cdkn1a, Itga3, S1pr1*, and *Vdr* and downregulation of *Runx3, Flt3*, and *Irf7* (Fig. 5K, left), which are all associated with a potential bias towards the myeloid lineage (79–86) and is consistent with the myeloid bias of aged HSCs. DNMT3A deficiency also promoted the transcription of *Il1r1*, which highlighted the potential implication that the loss-of-function of DNMT3A may accentuate the response of HSCs to circulating IL-1α from lung MPs. To further characterize the impact of DNMT3A deficiency, we analyzed RNA-seq of murine *Dnmt3a*^-/-^ and WT GMPs (87). Here, mutant GMPs significantly upregulated several genes associated with a monocytic phenotype (i.e., *F13a1, Il4ra, Gpnmb, Ccr2, C1qa*) and with immaturity (i.e., *Cd38, Flt3, Chil3, Ly6a*) and up-regulated *Il1r1* (Fig. 5K, right), suggesting that DNMT3A loss-of-function in hematopoietic progenitors leads to a greater production of myeloid progenitors with enhanced susceptibility to IL-1 signaling. More strikingly, when we then analyzed publicly available RNA-seq datasets of *Dnmt3a*^-/-^ and WT monocytes/mo-macs (88), we found that the absence of DNMT3A upregulates *Il1a, Il1b*, immunoregulatory molecules (i.e., *Cd274, Pdcd1lg2*), myeloid lineage markers (i.e., *Vcan, Ms4a3, Mpo, Elane, Retnlg, S100a8, S100a9, Prtn3*), metabolic enzymes associated with immunosuppression (i.e., *Arg1, Acod1, Hmox1*) (Fig. 5L, left) (89–91). Many of these markers were DEGs belonging to the IL-1α program (Table S3) and were also significant targets from an independent analysis of human DNMT3A-deficient and -proficient mo-macs (Fig. 5L, right) (92). In fact, other upregulated markers included those indicative of (i) immaturity (i.e., *CD38, CD48, CSF2RB*), (ii) innate immune sensing (i.e., *CGAS, NLRP3, MYD88*), (iii) cell survival (i.e., *BCL2L1, CDKN1A, TNF*), and (iv) an immunosuppressive phenotype (i.e., *CD274, TGFB1, IL18BP, NFE2L2*) with the potential to recruit other monocytes or Tregs (i.e., *CCL5, CCL7*). These changes suggested that DNMT3A regulates IL-1α and a broader myeloid phenotype in ways that are conserved in both mice and humans.

To experimentally validate these transcriptional data, we used a highly specific and potent small molecule inhibitor of DNMT3A on bone marrow monocytes from young and old mice. We observed a significant increase in IL-1α and IL-1β production by both young and old monocytes, treated with the DNMT3A inhibitor and stimulated with low-dose LPS (Fig. 5, M and N). This aligned with published work reporting increased IL-1α production by *Dnmt3a*^-/-^ mo-macs (93). Importantly, young monocytes treated with the DNMT3A inhibitor produced IL-1 at levels comparable to that of untreated, old monocytes (Fig. 5, M and N). This finding suggested that reducing DNMT3A activity can contribute to the aging-driven increase in ability for monocytic cells to produce IL-1. In contrast, the production of TNF-α exhibited a different profile; while an age-associated increase in production, like that of IL-1α, could be seen, DNMT3A inhibition increased TNF-α production in young but not in old monocytes (Fig. 5O). This was significant to us, as TNF-α was shown to also drive myeloid bias (94, 95). These data suggested that the age-associated regulation of IL-1α by DNMT3A decline does not necessarily extend to other cytokines capable of hematopoietic skewing. Importantly, we found that that this relationship between DNMT3A and IL-1α applies to human monocytes, as well (Fig. 5P).

### IL-1α-associated mRNA program stratifies patients on the basis of age and outcome

**Fig. 6:**
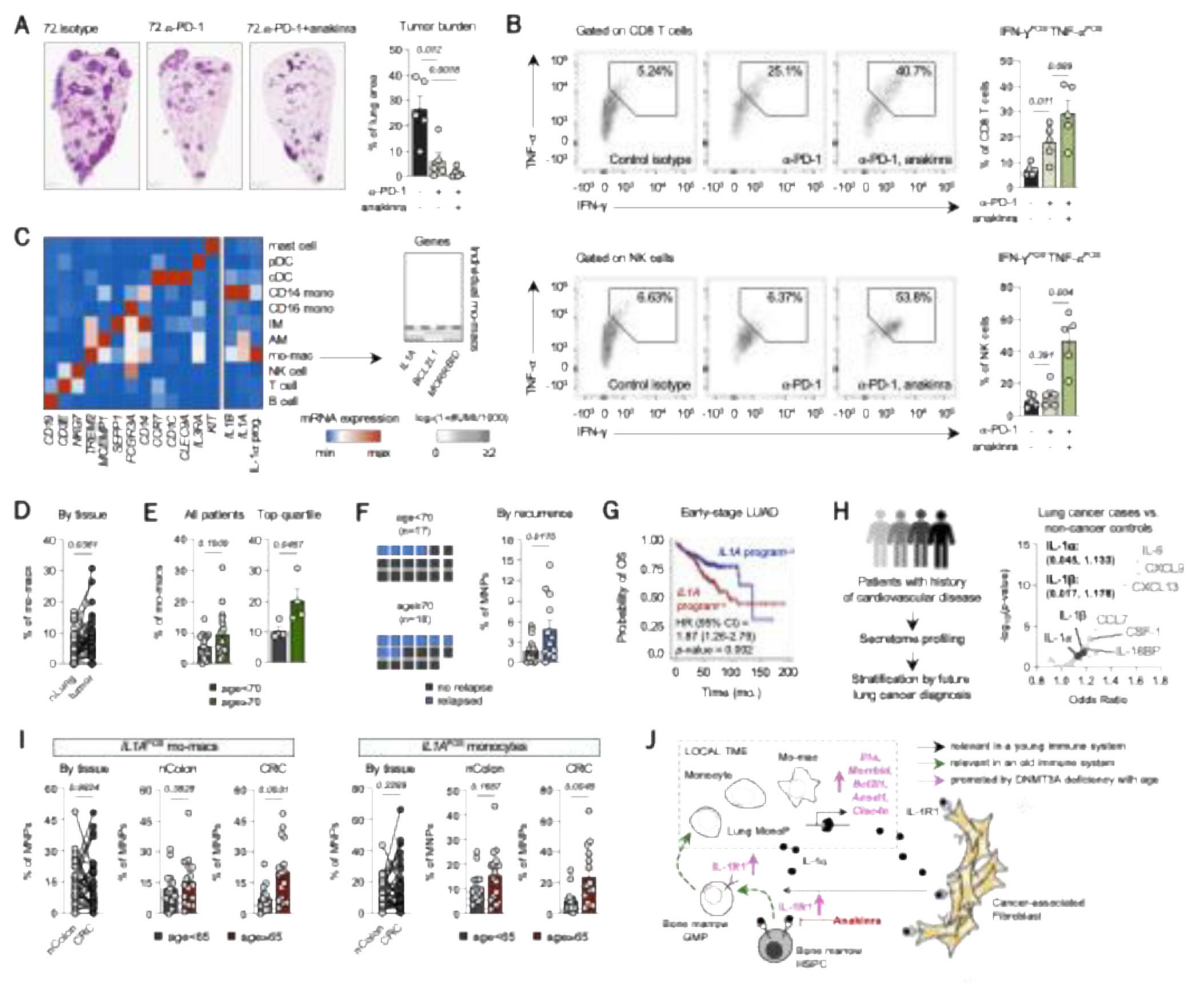
The IL-1α mRNA program is a marker for aging and outcome in cancer patients. (**A**) Tumor burden in lungs of old mice either treated with a control isotype antibody, PD-1 blocking antibody, or the combination of PD-1 blocking antibody and anakinra. (**B**) Frequency of cytotoxic CD8 T cells (top) and NK cells (bottom) in the tumor-bearing lungs of mice shown in (**B**). (**C**) (Left) mRNA expression of cell type-defining genes, *IL1A, IL1B*, and a composite score for the IL-1α-associated program, defined in Fig. 2, according to scRNA-seq of immune cells in lung tissues from NSCLC patients, and (right) heatmap of UMI counts of individual genes per cell (each individual row is a single cell) belonging to the mo-mac cluster. (**D**) Frequency mo-macs expressing the *IL1A* program (*IL1A*^POS^) in paired tumor and adjacent, normal lung (nLung) tissue specimens from NSCLC patients. (**E**) Frequency of *IL1A*^POS^ mo-macs in resected tumor lesions from (left) all patients and (right) just the top quartile of patients of each age group, based on frequency values after outlier exclusion. (**F**) (left) Distribution of patients that experienced a recurrence of cancer depending on age, and (right) frequency of tumoral *IL1A*^POS^ mo-macs per patient depending on status of recurrence. (**G**) Kaplan-Meier curve showing overall survival difference between high and low scorers of the *IL1A* mRNA program among NSCLC patients in The Cancer Genome Atlas (TCGA) and other studies belonging to the Genomic Data Commons Data Portal and independent datasets. (**H**) Associations between risk of lung cancer incidence and cytokines and chemokines measured in the blood of lung cancer patients, collected prior to diagnosis, and age-matched and smoking controls without cancer. Statistical values shown as (*p-*value, odds ratio). (**I**) Frequency of *IL1A*^POS^ mo-macs (left) and monocytes (right) in the CRC tumor and adjacent, non-involved colon tissues from 31 CRC patients and separated by age. (**J**) Summary diagram of mechanism. Raw data from panels (**C**)-(**F**) were obtained from Leader et al., 2021. Processed data for panel (**H**) were obtained from the Lung Cancer Cohort Consortium (LC3). Data are represented as mean ± SEM. *P*-value was computed by either paired two-tailed *t*-test or unpaired *t*-test.

Finally, we sought to explore the potential therapeutic relevance of our collective findings to the treatment of lung cancer. As PD-(L)1 blockade remains the first-line treatment for NSCLC, we assessed the efficacy of combining early intervention with anakinra with PD-1 blockade in old mice. Notably, combining these two modalities significantly enhanced tumor cell clearance (Fig. 6A); while PD-1 blockade improved cytotoxic CD8 T cell responses that were further elevated in combination with anakinra, anakinra played a unique role in rescuing the tumoricidal NK cell response that PD-1 blockade could not elicit alone (Fig. 6B). These data rationalized the combination of anakinra with standard-of-care immune checkpoint inhibition for lung cancer patients.

We then mined scRNAseq of our published map of treatment-naïve, surgically-resected NSCLC lesions of 35 patients, from whom we characterized immune cells in adjacent normal and tumor tissues (27). In our single-cell dataset, we were not able to detect MPs, likely owing to their scarcity and that millions of cells per patient would need to have been sequenced to capture them in sufficient numbers (33). Nevertheless, across major immune cell types identified by scRNAseq, we found that *IL1A* and *IL1B* mRNA were detected most strongly in classical monocytes and mo-macs. However, the broader IL-1α mRNA program (defined using the hallmark genes *IL1A, MORRBID, BCL2L1, CDKN1A, IL1RN, CLEC6A, SPP1*) correlated strongly with the TREM2 mo-mac cluster as exhibiting the dominant signal (Fig. 6C, left). Examining the single-cell expression of some of these genes showed that a discrete subset of mo-macs expressed the IL-1α mRNA program (*IL1A*^POS^) (Fig. 6C, right). These *IL1A*^POS^ mo-macs were significantly enriched in primary resected tumors, compared to normal tissues, of NSCLC patients (Fig. 6D). These tumor-associated *IL1A*^POS^ mo-macs were greater in frequency in the tumors of older patients (age ≥ 70, n=18) than in younger patients (age < 70, n=17) (Fig. 6E); this age-dependent difference was not seen in adjacent, normal tissues (*p*=0.94). These data supported our study of the IL-1α mRNA program as a tumoral, molecular signature that is associated with age. Moreover, we found that a greater proportion of older patients experienced a recurrence (n=8 of 18, 44.4%), compared to younger patients (n=4 of 17, 23.5%) (Fig. 6F, left). Strikingly, we found a significant enrichment for tumoral *IL1A*^POS^ mo-macs in the tumors of recurrent patients, compared to those who did not recur (Fig. 6F, right), indicating a predictive association between the abundance of *IL1A*^POS^ mo-macs in primary resected tumors and recurrence.

Finally, we sought to assess the prognostic value of the broader IL-1α mRNA program. We first leveraged datasets integrated from the Genomic Data Commons Data Portal, including The Cancer Genome Atlas (TCGA), to test the relevance of the IL-1α mRNA program in a much larger population. Overall survival analysis using a Kaplan-Meier estimator and Cox regression indicated that high expressors of the IL-1α mRNA program were predicted to exhibit a worse prognosis, compared to low expressors (Fig. 6G). We then tested the probative value of the IL-1α mRNA program as a predictor of lung cancer risk. Here, we harnessed the circulating proteome of individuals, whose blood was collected up to three years prior to lung cancer diagnosis and abundance levels of over 1,000 proteins were compared between lung cancer patients and smoking, age-matched controls (96). We found that both IL-1α and IL-1β were predictive of elevated risk for lung cancer risk (Fig. 6H).

As we showed that the IL-1α/IL-R1 axis of age-enhanced emergency myelopoiesis also contributes to the aging-driven progression of CRC, we also sought to explore the relevance of the IL-1α mRNA program in human CRC. We analyzed tumor and non-involved colon tissues of 31 patients with CRC by scRNAseq. Unsupervised clustering identified discrete subsets of mo-macs and monocytes that expressed the IL-1α mRNA program. These *IL1A*^POS^ mo-macs and monocytes were comparably distributed between tumor and normal tissues, but strikingly, the tumoral cells were significantly enriched in colon tissues from older than from younger patients (Fig. 6I). These observations strengthened the point that our observations in the lung cancer setting are relevant for individuals with other solid tumors.

## Discussion

In this study, we established that aging of the immune system is one key determinant of lung cancer outcome. We showed that a major underlying mechanism involves the enhanced tumor-induced myelopoietic response that is seen in old mice. Specifically, we demonstrated that this results in the local accumulation of myeloid progenitor-like cells. While it remains to be proven whether these cells are able to locally supply monocytes and mo-macs to the TME, we found that these cells produce significantly higher levels of IL-1α with age (Fig. 6J). The age-associated decline in DNMT3A expression contributes to this phenotype, but the cause for this downregulation also remains unclear and warrants independent study. One hypothesis could be that the loss of DNMT3A destabilizes heterochromatin and facilitates access to endogenous retrotransposable elements, whose genetic byproducts can activate innate sensing pathways and promote IL-1α release (97–104). We further demonstrated that a local trigger of IL-1 could be tumor cell debris, which aligns with published reports of environmental particulates that have been shown to cause tissue damage and elicit IL-1 release from lung-resident myeloid cells (105). The broader etiology is likely not singular, as a number of variables linked to increased TLR ligand presence, such as age-associated cilia dysfunction, smoking, and microbial dysbiosis, promote lung cancer (106, 107).

As they may pertain to patients, our data illuminate the therapeutic window for anakinra therapy during lung cancer progression, which appears analogous to results from phase III trials that sought to test the therapeutic potential of IL-1β blockade. In the Canakinumab Anti-Inflammatory Thrombosis Outcomes Study (CANTOS) that reported on the use of IL-1β blockade with canakinumab to reduce the risk of cardiovascular events, a planned secondary analysis demonstrated reduced risk of *de novo* diagnoses of lung cancer risk (108). Notably, as we demonstrate in our studies of early versus late IL-1 blockade, trials of canakinumab in the advanced cancer setting failed to demonstrate similar clinical benefit. Our findings highlight several important considerations, including (i) the role of IL-1α, which is not inhibited with canakinumab alone, and (ii) the role of timing (i.e., whether IL-1 inhibition is introduced during the early stages of tumor initiation versus once tumors are fully established). To date, no other trials are testing blockade of IL-1R1 signaling for lung cancer therapy (109). For these reasons, our observation that early, but not late, use of anakinra delays lung cancer progression by normalizing myelopoiesis, therefore, rationalizes prophylactic use of IL-1-based interventions, like IL-1R inhibition, for the prevention or early management of nascent NSCLC, with the added nuance that earlier administration is likely to be more effective for older patients.

This therapeutic stratagem is also likely to be relevant for age-related co-morbidities that are linked to poorer prognoses for lung cancer patients, such as CHIP (110), since it is associated with lung cancer risk, with age, and with enhanced myelopoiesis. Moreover, because CHIP is linked to various chronic inflammatory conditions (e.g., cardiovascular disease, chronic kidney disease, arthritis, cirrhosis) (111–116) that would likely further polarize bone marrow and promote myelopoiesis, targeting pathways that link CHIP with lung cancer would effectively ameliorate the pro-tumorigenic contributions of these co-morbidities. Our data identified the steady-state decline in DNMT3A as a contributing factor to the enhanced production of IL-1α by monocytic cells, providing novel mechanistic evidence for the relationship between CHIP, inflammaging, and lung cancer.

Finally, as our study elucidated the role of the IL-1α/IL-1R1 axis in aging-driven emergency myelopoiesis during the progression of colorectal and pancreatic cancer, we suspect that this mode of pathogenic myelopoiesis is active during the growth of other solid tumors and propose that the blockade of IL-1R1 signaling will likely also be therapeutic in different cancer settings. The importance of these other orthotopic models is underscored by the fact that some past studies have observed delayed tumor growth in older mice (117–121), while others have reported – using the same sub-cutaneous models – accelerated growth of tumors in old mice (122, 123). More interestingly, recent work with genetically-engineered murine models proposed that aging reduces tumor initiation and growth upon inactivating tumor suppressor genes in young and old mice (124, 125). These bodies of work suggest that the pro-tumoral effect of aging is driven mostly by the microenvironment – in alignment with our work – rather than by the aging of the tumor cells themselves. Furthermore, we find that our findings are consistent with recent observations that hematopoietic aging drives systemic increases in immunosenescence, which promotes lung cancer (126–128).

Outside of murine models, aging does not occur in humans within a vacuum. Over the lifetime, a variety of perturbations will influence the dynamics of hematopoiesis and of tissue-resident immune cells in the lungs and other organs. And many of these age-associated afflictions, like obesity, infections, or physical injury, are known to elicit an emergency myelopoietic response and may likely underscore aging-related differences in other causes of mortality (e.g., COVID-19). Therefore, our work proffers a key step forward in our understanding of aging, by establishing the unique role played by an aged hematopoietic compartment in cancer progression. Considering the immune system’s amenability to therapeutic interventions, our findings suggest that myeloid cell-targeting strategies should be prioritized for cancer prevention and for treatment methods in more elderly patients.

## Supplementary Material

Supplementary Materials

Table S1

Table S2

Table S3

## Data Availability

All murine sequencing data are publicly available under GEO accession number GSE275150. No custom code was generated for this study.

## One-Sentence Summary

With age, myeloid progenitors accumulate in lung tumors and produce IL-1α to fuel pro-tumorigenic emergency myelopoiesis. (121 characters with spaces)
