# Supplementary material for: Hematopoietic aging promotes cancer by fueling IL-1α-driven emergency myelopoiesis
Source: Science. Author manuscript; Available in PMC 2024 Oct 25. (PMC7616710; doi:10.1126/science.adn0327)
Supplement: Supplementary Materials [file EMS199319-supplement-Supplementary_Materials.pdf]

Science
AAAS

Supplementary Materials for

Hematopoietic aging promotes cancer by fueling IL-1α–driven emergency myelopoiesis

Matthew D. Park *et al.*

Corresponding author: Miriam Merad, miriam.merad@mssm.edu

DOI: 10.1126/science.adn0327

The PDF file includes:

Materials and Methods
Figs. S1 to S9
Tables S1 to S3
References

Other Supplementary Material for this manuscript includes the following:

MDAR Reproducibility Checklist

Materials and Methods

Mice. For this study, the following murine strains were used: 7-week-old and 72-week-old C57BL/6J (JAX, #000664), B6.SJL-*Ptprc*^{a}*Pepc*^{b}/BoyJ (CD45.1) (JAX, #002014), C57BL/6J.129S7-*Il1r1*$^{tm1/mx}$/J (IL-1R1$^{-/-}$) (JAX, #003245) mice, C57BL/6J-*Il1a*^{em1Tdk}/J (IL-1α$^{-/-}$) (JAX, #037967) mice, and 72-week-old C57BL/6J-*Trem2*^{em2Adiuj}/J (*Trem2*$^{-/-}$) (JAX, #027197). *Ms4a3*^{CRE} reporter mice were gifted from Dr. Florent Ginhoux. *Kras*$^{G12D/+}$ *p53*$^{-/-}$ genetically-engineered (GEMM) mice were generated by crossing *LSL-Kras*$^{G12D/+}$ mice (JAX, #008179) with *p53*$^{fl/fl}$ mice (JAX, #008462). All mice were housed at the Icahn School of Medicine at Mount Sinai for a minimum of a week before experimental use. Mice were maintained at specific-pathogen-free health status in individually-ventilated cages at 21-22 deg C and 39-50% humidity. All animal procedures were approved by the Institutional Animal Care and Use Committee (IACUC) of the Icahn School of Medicine at Mount Sinai. Mice within experiments were age- and sex-matched. All studies performed on mice were done in accordance with the IACUC at the Icahn School of Medicine at Mount Sinai.

Orthotopic lung cancer model. To model primary lung adenocarcinoma, mice were intravenously injected via the tail vein with either 1.5x10^{5} tumor cells derived from *Kras*$^{LSL-G12D/+}$ *Trp53*$^{-/-}$;*Rosa26*^{A3Bi};*Rag1*$^{-/-}$ (KPAR) mice or 5.0x10^{5} GFP-expressing tumor cells derived from *Kras*$^{LSL-G12D/+}$ *Trp53*$^{-/-}$ (KP) mice , which were generated, as previously described. KPAR and KP cells were grown in complete cell culture medium (DMEM, 10% fetal bovine serum, 1% penicillin/streptomycin) and detached for use at 80% confluence using 0.25% trypsin. Tumor-bearing lungs were analyzed at indicated timepoints; the left lung lobe was fixed in paraformaldehyde, embedded in paraffin, and examined as 4 µm cross-sections. Upon hematoxylin and eosin staining, lung tissue sections were scanned on slides using an Olympus digital scanner and analyzed using the Panoramic viewer and QuPath software.

Orthotopic colorectal cancer model. For orthotopic transplantation into the cecum, *Apc*$^{fl/fl}$ *Kras*$^{LSL-G12D/+}$ *p53*$^{fl/fl}$ *Smad4*$^{fl/fl}$ (AKPS) colon tumor organoids were dissociated using TrpLE Express (Thermo) for 10 min at 37 deg C and single-cell suspensions were resuspended in base-membrane extract (BME, R&D Systems). 1x10^{5} cells in 10 µL were injected into the cecum using a 30G insulin syringe. Tumors were analyzed at five weeks post-injection; tumor volume was quantified at endpoint using calipers.

Orthotopic pancreatic cancer model. Tumor cells derived from *Kras*$^{LSL-G12D/+}$ *Ptf1a*^{CRE} (KC) mice (*127*) were used for transplantation experiments. In brief, 1x10^{4} tumor cells were orthotopically grafted into the pancreas of syngeneic immunocompetent C56BL/6J mice, as previously described (*128*). Animals were sacrificed after three weeks. Fresh tumor samples were weighed to determine tumor burden.

Vascular permeability. To assess vascular integrity, mice were intravenously injected with either Dextran that was conjugated to an APC fluorophore or the Evans Blue dye. After 15 minutes, mice were pericardially perfused with 50 mL of PBS. Lung tissue was digested to then measure leaked contrast material into the parenchyma.

***In vivo* labeling of circulating immune cells.** To partition immune cells in the intravascular circulation and in the extravascular parenchyma of peripheral tissues, an APC-conjugated antibody against the murine CD45 antigen was administered via the tail vein 12 min prior to euthanasia to label all cells in the intravascular space.

Antibody treatments. To assess the effect of blocking IL-1 signaling on the progression of lung cancer lesions, mice were either given control PBS or the appropriate isotype controls, 250 µg of IL-1α neutralizing antibody (BioXcell, Clone ALF-161, Cat. #BE0243) (i.v.), 50 µg of IL-1β neutralizing antibody (BioXcell, Clone B122, Cat. #BE0246) (i.v.), or the anti-IL-1R antagonist anakinra (500 µg per mouse, i.p.) (Swedish Orphan Biovitrum) every other day.

Bone marrow transplantation. Recipient mice were irradiated with two doses of 5.5 Gy that were administered 6 hrs apart. Donor bone marrow cells (5x10^{6}) were retro-orbitally transferred into irradiated recipient mice. A period of 7-8 weeks was granted to ensure engraftment. The recipients were then supplemented with

sulfamethoxazole / trimethoprim for three weeks. Reconstitution was assessed by flow cytometric analysis of inflammatory or $Ly6C^{HI}$ monocytes in the lungs.

Functional genomics using Perturb-map. The Perturb-map method for spatial functional genomics was used to evaluate IL-1R1-proficient and -deficient KPAR cells within a shared tumor microenvironment, as previously described (*129, 130*).

Mass cytometry: Processing and analysis of cell suspensions by Cytometry by Time of Flight (CyTOF) were performed. Briefly, $3x10^6$ cells in suspension were stained for viability with Cell-ID Intercalator-103 Rh for 15 min at 37 deg C, followed by staining for the surface markers in flow buffer with anti-mouse CD16/CD32 blocking antibody (eBioscience) on ice for 30 min. Then, cells were fixed and permeabilized using eBioscience FOXP3/Transcription Factor Staining Buffer Set (Invitrogen) following the manufacturer's instructions. Fixed and permeabilized cells were then stained with epitope-tag antibodies on ice for 1 hour followed by incubation with 125 nM IR intercalator (Fluidigm) diluted in PBS containing 2.4% formaldehyde at RT for 30 min, washed and stored in FBS (10% DMSO) at -80 deg C until acquisition. The samples were acquired on either a CyTOF2 or Helios (both Fluidigm) at an event rate of <500 events/second. Antibodies were purchased purified and conjugated in-house using MaxPar X8 Polymer Kits (Fluidigm) according to the manufacturer's instructions. CyTOF data was analyzed using Cytobank where gating was performed for single, live, and PC positive cells. Each PC population was then gated by its corresponding epitope positivity (F8 KO: C.Ollas.VSV-G, Il1r1 KO: HA.Ollas.V5, Il1r2 KO: HA.S.VSV-G). The antibodies used include anti-V5 tag mouse mAb (Clone R960-25, Cat. #R960-25), anti-Protein C tag mouse mAb (Clone HPC4, Cat. #A01774), anti-VSVg tag rabbit pAb (Cat. #PA1-30138), anti-Protein C tag mouse mAb (Clone HPC4, Cat. #A01774-100), anti-NWS tag mouse mAb (Clone 5A9F9, Cat. #A01736), anti-VSVg tag rabbit mAb (Clone E8S5G, Cat. #93372), anti-human αSMA rabbit mAb (Clone D4K9N, Cat. #76113S), anti-V5 tag pAb (Cat. #NB600-381), anti-CD45 in AF555 (Clone D3F8Q, Cat. #19581), anti-mouse CD16/32 (Clone 93, Cat. #16-0161-82), anti-HA tag 147 Sm (Clone 6E2, Cat. #2367), anti-V5 tag 152 Sm (Clone R960-25, Cat. #R960-25), anti-VSVg tag 158 Gd (Cat. PA1-30138), anti-NWS tag 159 Tb (Clone 5A9F9, Cat. #A01732), anti-S tag 165 Ho (Clone SBSTAGa, Cat. #ab24838), anti-Olias tag 153 Eu (Clone L2, Cat. #MA5-16125), anti-Protein C tag 171 Yb (Clone HPC4, Cat. #A01774).

Cyclic immunofluorescence (CyCIF): FFPE sections were prepared and stained with a six antibody panel. Briefly, 5 µm thick FFPE tissue sections were baked at 60 deg C overnight, deparaffinized in xylene, and rehydrated in decreasing concentrations of ethanol solutions (100%, 90%, 70% and 50%). Antigen retrieval was performed by incubating the slides in Antigen Retrieval Solution (pH 9, Dako) at 95 deg C for 30 minutes. Slides were then cooled at RT for 30 minutes and washed with TBS, followed by photobleaching by immersing them in a bleaching solution (4.5% H_2O_2, 20 mM NaOH in PBS) with LED light exposure for 2 x 45 min to reduce autofluorescence. To mitigate non-specific antibody binding, slides were washed for 3 x 5 min with 1X PBS and then incubated overnight with secondary antibodies (anti-rat, anti-mouse, and anti-rabbit) diluted 1 to 1000 in 150 µL of Odyssey Blocking Buffer at 4 deg C in the dark. Slides were subsequently washed 3x with 1X PBS before photobleaching them again for 2 x 45 min. For each round of CyCIF, samples were incubated overnight at 4 deg C in the dark with Hoechst 33342 (1 to 10,000 dilution; Thermo Fisher Scientific) for nuclear staining along with either primary conjugated antibodies or primary unconjugated antibodies diluted in 150 µL of Odyssey Blocking Buffer (LI-Cor). Incubation with primary unconjugated antibodies was followed by secondary antibody incubation at room temperature for 2 hours in the dark. Then, the slides were washed for 3 x 5 min and mounted with 200 µL of 70% glycerol. Slides were automatically imaged on the RareCyte Cytefinder II HT using the following channels: UV, cy3, cy5, and cy7. Imaging was performed with the following parameters: Binning: 1 x 1; Objective: 20x; Numerical Aperture: 0.75; Resolution: 0.325 um/pixel. Image exposures were optimized for each channel to avoid signal saturation but kept constant across samples. After imaging, slides were placed in containers of 1X PBS and heated in a water bath for 1 hour to remove coverslips. Between each cycle, slides were photobleached for 2 x 45 min and washed 3 x 5 min in 1X PBS.

The preanalytical CyCIF image processing (stitching, registration, illumination correction, segmentation, and single-cell feature extraction) was performed using the MCMICRO pipeline, an open-source multiple-choice microscopy pipeline (full codes available on GitHub at https://github.com/labsyspharm/mcmicro). For the

generation of probability maps, a trained U-Net model, UnMicst v2, was used followed by a marker-controlled watershed used for single-cell segmentation. A diameter range of 3 to 60 pixels was used for nuclei detection. UnMicst generated probability maps were then passed to S3segmenter to generate nuclear segmentation masks. The cytoplasmic area was captured by expanding the nuclei mask by 3 pixels. After generating the segmentation masks, the mean fluorescence intensities of each marker for each cell were computed, resulting in a single-cell data table for each acquired whole-slide CyCIF image. The X/Y coordinates of annotated histologic regions on the whole-slide image were used to extract the quantified single-cell data of cells that lie within the ROI range.

Multiple approaches were taken to ensure the quality of the single-cell data. At the image level, the cross-cycle image registration and tissue integrity were reviewed; regions that were poorly registered or contained severely deformed tissues and artifacts were identified, and cells inside those regions were excluded. Antibodies that gave low confidence staining patterns by visual evaluation were excluded from the analyses. The quality of the segmentation was assessed, and the segmentation parameters were iteratively modified to improve the accuracy of the segmentation masks. On the single-cell data level, correlations of DNA staining intensities in different cycles were used to filter out cells that were lost in the cyclic process with a threshold of correlation coefficient less than 0.8. Tumors corresponding to each PC were identified by manual ROI selection utilizing scimap for downstream analysis. The markers used for ROI selection were (VSV-G, V5, C, and NWS).

***In vitro* culture.** *Stimulation of KPAR with IL-1:* KPAR cells were cultured for at least three passages. These tumor cells were then counted and plated at 10,000 cells per well in 6-well plates either in the presence of control media (RPMI, supplemented with 10% FBS, 1% penicillin/streptomycin) or with either 25 ng/mL or 100 ng/mL of recombinant murine IL-1α (R&D Systems, Cat. #400-ML-005/CF) or IL-1β (R&D Systems, Cat. #401-ML). Cell viability and numbers were measured at 24, 48, and 72 hours post-plating.

Genetic knockdown of IL-1R1 in KPAR: Knockdown of IL-1R1 expression by KPAR cells were generated by CRISPR-Cas9-mediated genetic deletion. Briefly, a sgRNA targeting *Il1r1* (ACAGCGGCTCCACATTGCCG) was cloned into the pSpCas9(BB)-2A-Puro (PX459) vector. KPAR cells were transfected with 5 µg of the PX459 vector using Lipofectamine 3000 followed by puromycin selection. CRISPR knockout was confirmed by flow cytometry. As a control, KPAR cells were transfected with a PX459 vector containing a non-targeting sgRNA (ACCTGATACGTCGTCGCGTA).

Ex vivo stimulation of bone marrow monocytes: For culture studies using bone marrow monocytes, bone marrow (femur and tibia of both hind legs) were flushed with 3 mL of PBS. Bone marrow monocytes were enriched from these suspensions by depleting magnetically labeled non-target cells (T cells, B cells, NK cells, dendritic cells, erythroid cells, and granulocytes), according to the manufacturer's instructions (Miltenyi Biotec, Cat. #130-100-629). Bone marrow monocytes were then plated in 96-well plates, in the presence of either control media (RPMI, supplemented with 10% FBS, 1% penicillin/streptomycin), 25 µM of the DNMT3A inhibitor DNMT3A-IN-1 (MedChemExpress, Cat. #HY-144433), or 25 µM of each of the reverse transcriptase inhibitors (RTi) abacavir and lamivudine. Cells were treated for 48 hours, after which cells were centrifugated at 1,500 rpm for 5 min and stimulated in media with lipopolysaccharide (LPS) (InvivoGen, Cat. #tlrl-eblps) for 2 hours in the presence of Brefeldin A. Cells were then fixed and stained for cell surface expression and intracellular production of IL-1α.

Single-cell RNA sequencing. *Sample preparation*: Single-cell suspensions from lung tissues were obtained, as described above. Samples were broadly enriched for $CD45^{POS}$ cells by fluorescence-activated cell sorting, and these cells were suspended in PBS supplemented with 0.5% BSA. Samples were loaded onto the 10x Genomics Next GEM 5' assay, as per the manufacturer's instructions, for a target cell recovery of 10,000 cells per lane. Libraries were constructed, according to manufacturer's instructions. All libraries were quantified via Agilent 2100 hsDNA Bioanalyzer and KAPA library quantification kit (Roche, Cat. #0797014001). Libraries were sequenced at a targeted depth of 25,000 reads per cell; all libraries were sequenced using the Illumina NovaSeq S2 100 cycle kit.

scRNAseq analysis: Gene expression reads were aligned to the mm10 reference transcriptome and count matrices were generated using the default CellRanger 2.1 workflow, using the 'raw' matrix output. Following alignment,

barcodes matching cells that contained > 500 unique molecular identifiers (UMIs) were extracted. From these cells, those with transcripts > 25% mitochondrial genes were filtered from downstream analyses. Matrix scaling, logarithmic normalization, and batch correction via data alignment through canonical correlation analysis, and unsupervised clustering using a *K*-nn graph partitioning approach were performed as previously described. Differentially expressed genes were identified using the FindMarkers function (*Seurat*). Mean UMI were imputed to determine logarithmic fold changes in expression between cell states to further the analysis of markers of interest. Gene set enrichment analysis was performed using the Enrichr database. Other R packages used include: *scDissector* v.1.0.0; *shiny* v.1.7.; *ShinyTree* v.0.2.7; *heatmaply* v.1.3.0; *plotly* v.4.10.0; *ggvis* v.0.4.7; *ggplot2* v.3.3.5; *dplyr* v.1.0.7; *Matrix* v.0.9.8; *seriation* v.1.3.5.

Survival analyses were performed using the *survival, survminer,* and *gtsummary* R packages.

Human study participants. Patients' informed consent was obtained using the Universal Consent for Mount Sinai Biorepository (IRB Human Subjects Electronic Research Applications 20-01197) and the study-specific consent form for IRB Human Subjects Electronic Research Applications 10-00472A, following the protocols reviewed and approved by the Institutional Review Board (IRB) at the Icahn School of Medicine at Mount Sinai. Patients provided written consent for the analysis of peripheral blood mononuclear cells (PBMCs). Samples were collected from patients undergoing surgical resection at the Mount Sinai Hospital (New York, NY) under the purview of a collaboration between the Department of Thoracic Surgery, the Mount Sinai Biorepository, and the Department of Pathology. PBMC analyses were completed under IRB Human Subjects Electronic Research Applications 10-00472A.

Flow cytometry. Single-cell suspensions from perfused murine lungs were obtained upon digestion of naïve or tumor-bearing lung tissues using collagenase IV (0.25 mg/mL; Sigma, Cat. #C5138-1G) at 37 deg C for 30 min while spun at 80 r.p.m. Samples were passed through a 70 µm cell strainer and lysed for red blood cells. Cells were stained in flow cytometry buffer (PBS, 2% bovine serum albumin, 5 mM EDTA) with the following anti-mouse monoclonal antibodies: CD45.1/.2 (BV510, Clone 30F-11; Cat. #103138), CD45.1 (APC, Clone A20; Cat. #17-0453-82), CD135 (PerCP-Cy5.5, Clone A2F10; Cat. #46-1351-82), CD117 (PE-Cy7, Clone 2B8; Cat. #25-1171-82), CD16/32 (Alexa Fluor 700, Clone 93; Cat. #56-0161-82), CD11b (APC-Cy7, Clone M1/70; Cat. #47-0112-82), Ly6G (APC-Cy7, Clone 1A8; Cat. #127624), CD3ε (APC-Cy7, Clone 17A2; Cat. #47-0032-82), B220 (APC-Cy7, Clone RA3-6B2; Cat. #47-0452-82), Ter-119 (APC-Cy7, Clone TER-119; Cat. #47-5921-82), NK1.1 (APC-Cy7, Clone PK136; Cat. #108724), Sca-1 (APC-Cy7, Clone D7; Cat. #108125), CD34 (Pacific Blue, Clone RAM34; Cat. #48-0341-82), Ly6C (BV605, Clone HK1.4; Cat. #128035), CD115 (biotinylated, Clone AFS98; Cat. #13-1152-85), CD115 (PE, Clone AFS98; Cat. #12-1152-82), Anti-biotin streptavidin (Cat. #405232), IL-1α (PE, Clone ALF-161; Cat. #12-7011-82), phosphorylated STAT6 (APC, Clone CHI2S4N; Cat. #17-9013-42), Ki-67 (FITC, Clone SOLA15; Cat. #11-5698-82), CD11b (PerCP-Cy5.5, Clone M1/70; Cat. #101230), CD64 (PE, Clone X54-5/7.1; Cat. #139304), CD2 (PE-Cy7, Clone RM2-5; Cat. #100114), Siglec-F (APC-Cy7, Clone E50-2440; Cat. #565527), CX3CR1 (Pacific Blue, Clone SA011F11; Cat. #149023), CD11c (BV785, Clone N418; Cat. #117336), CD8α (FITC, Clone 53-6.7; Cat. #11-0081-82), NKp46 (PerCP-Cy5.5, Clone 29A1.4; Cat. #137610), CD69 (PE, Clone H1.2F3; Cat. #12-0691-82), TNF-α (PE-Cy7, Clone TN3-19; Cat. #25-7423-82), IFN-ɣ (APC-Cy7, Clone XMG1.2; Cat. #505850), CD4 (APC, Clone GK1.5; Cat. #17-0041-82), CD3ε (Pacific Blue, Clone eBio500A2; Cat. #48-0033-82), KLRG1 (BV650, Clone 2F1; Cat. #138405), FoxP3 (PE, Clone FJK-16s; Cat. #12-5773-82), DNMT3A (Clone 64B1446, Cat. #MA5-16171). Human peripheral blood mononuclear cells (PBMCs) were stained in flow cytometry buffer with the following anti-human monoclonal antibodies: CD34 (FITC, Clone RAM34, Cat. #11-0341-82), CD34 (APC, Clone 8G12; Cat. #2242482), CD38 (PE-Cy7, Clone HIT2; Cat. #303515), CD90 (Clone 5E10, Cat. #328119), DNMT3A (Clone 64B1446, Cat. #MA5-16171), CD3 (APC-Cy7, Clone HIT3a; Cat. #300317), CD19 (APC-Cy7, Clone HIB19; Cat. #302217), CD14 (APC-Cy7, Clone HCD14; Cat. #325619), CD10 (APC-Cy7, Clone HI10a; Cat. #312212), CD56 (APC-Cy7, Clone HCD56; Cat. #318331), CD8α (APC-Cy7, Clone RPA-T8; Cat. #301015), CD235α (APC-Cy7, Clone HI264; Cat. #349115), CD3 (APC-Cy7, Clone HIT3a; Cat. #300317), CD11b (APC-Cy7, Clone ICRF44; Cat. #301341), CD2 (APC-Cy7, Clone RPA-2.10; Cat. #300219), CD4 (APC-Cy7, Clone RPA-T4; Cat. #300517). For intracellular staining, cells were fixed with either BD Cytofix/Cytoperm kit or the eBiosciences Foxp3 transcription factor kit, both according to the manufacturer's instructions. To assess the

activation potential of T cells, cells were incubated with 10 μg/mL Brefeldin A, 0.2 μg/mL ionomycin, and 0.5 μg/mL phorbol myristate acetate for 3 hrs at 37 deg C. Upon staining, cells were analyzed using a BD LSR Fortessa. Flow cytometry data were acquired using the FACS Diva software v.7 (BD) and were analyzed using FlowJo.

Sequencing Datasets: The following external bulk and single-cell RNA sequencing (scRNAseq) datasets were used for analyses shown in this study: GSE245236, PRJEB56666, GSE183219, GSE163503, GSE151333, GSE120221, GSE162607, GSE168807, GSE74166, GSE237599, GSE206030, and GSE154826. scRNAseq data generated for the colorectal cancer cohort will be published in a separate manuscript. Murine scRNAseq data generated by this study is available using the GEO accession code GSE275150.

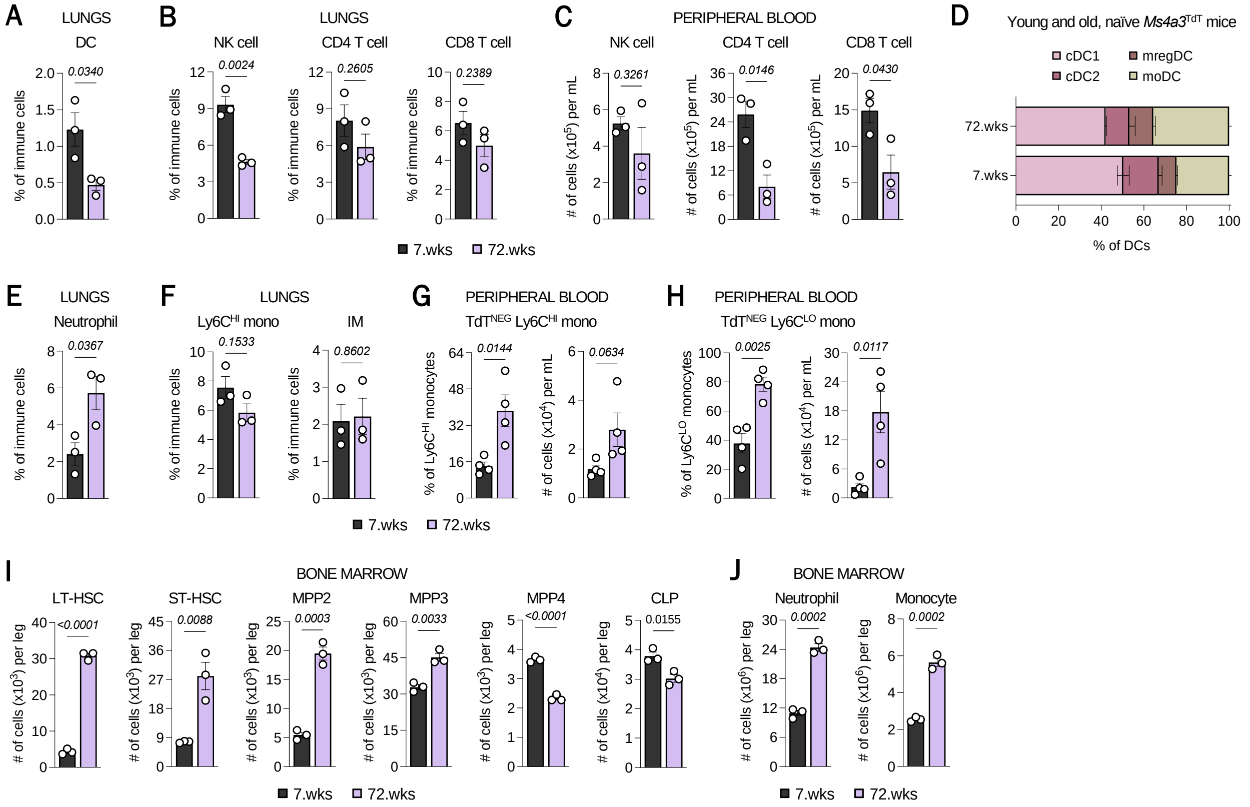

Fig. S1. Differentiated myeloid cells and myeloid progenitors accumulate in the aging bone marrow. Frequency of (**A**) DCs and (**B**) NK cells, CD4, and CD8 T cells in the lungs of naïve young (7-week-old, 7.wks) and old (72-week-old, 72.wks) mice. (**C**) Frequency of NK cells, CD4, and CD8 T cells in the peripheral blood of naïve young and old mice. (**D**) Frequency distribution of DCs identified from scRNAseq of TdT^{NEG} and TdT^{POS} myeloid cells in naïve lungs of young and old *Ms4a3*^{CRE}-TdT mice. Frequency of (**E**) neutrophils and (**F**) $Ly6C^{HI}$ monocytes and IMs in the lungs of naïve young and old mice. Frequency (left) and absolute number (right) of (**G**) TdT^{NEG} $Ly6C^{HI}$ monocytes and (**H**) TdT^{NEG} $Ly6C^{LO}$ monocytes in the peripheral blood of naïve young and old mice. Absolute number of (**I**) LT-HSC (Lin^{NEG} $c\text{-}Kit^{POS}$ $Sca\text{-}1^{POS}$ $CD135^{NEG}$ $CD48^{NEG}$ $CD150^{NEG}$), ST-HSC (Lin^{NEG} $c\text{-}Kit^{POS}$ $Sca\text{-}1^{POS}$ $CD135^{NEG}$ $CD48^{NEG}$ $CD150^{POS}$), MPP2(Lin^{NEG} $c\text{-}Kit^{POS}$ $Sca\text{-}1^{POS}$ $CD135^{NEG}$ $CD48^{POS}$ $CD150^{NEG}$), MPP3 (Lin^{NEG} $c\text{-}Kit^{POS}$ $Sca\text{-}1^{POS}$ $CD135^{NEG}$ $CD48^{POS}$ $CD150^{POS}$), MPP4 (Lin^{NEG} $c\text{-}Kit^{POS}$ $Sca\text{-}1^{POS}$ $CD135^{POS}$), and CLP (Lin^{NEG} $c\text{-}Kit^{POS}$ $Sca\text{-}1^{POS}$ $IL\text{-}7R\alpha^{POS}$) and of (**J**) neutrophils and monocytes in bone marrow of naïve young and old mice. Across all experiments, n=3-5 mice were used per group. Data shown in panels (**A**)-(**D**) are each representative of one independent experiment; in panels (**E**)-(**J**) are each representative of two independent experiments. Across all panels, data represent mean ± SEM. *P*-values were computed by unpaired *t*-test. (DC, conventional dendritic cell; NK cell, natural killer cell; IM, interstitial macrophage; LT-HSC, long-term hematopoietic stem cell; ST-HSC, short-term hematopoietic stem cell; MPP, multipotent progenitor; CLP, common lymphoid progenitor)

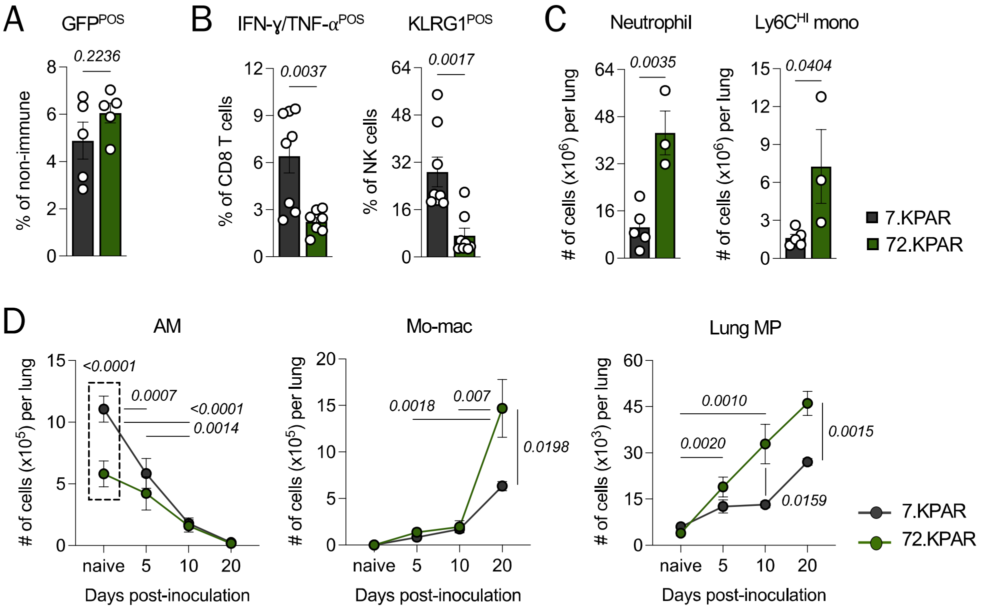

Fig. S2. A poorer lymphoid and a stronger myelopoietic responses underlie enhanced tumor growth in old mice. (**A**) Frequency of seeded GFP^{POS} tumor cells in the lung parenchyma of young (7-week-old, 7.KP-GFP) and old (72-week-old, 72.KP-GFP) mice 24 hours post-inoculation. (**B**) Frequency of cytotoxic CD8 T cells (CD45^{POS} CD3ε^{POS} CD8^{POS} IFN-γ^{POS} TNF-α^{POS}) and activated NK cells (CD45^{POS} NKp46^{POS} CD3ε^{NEG} KLRG1^{POS}) in tumor-bearing lungs of young (7.KPAR) and old (72.KPAR) mice at 16 days post-tumor inoculation. (**C**) Absolute number of lung neutrophils and Ly6C^{HI} monocytes (mono) in the tumor-bearing lungs of young and old mice at 16 days post-tumor inoculation. (**D**) Absolute number of tissue-resident AMs (CD45^{POS} CD64^{POS} Siglec-F^{POS} CD11c^{POS}), MPs (CD45^{POS} Lin^{NEG} Sca-1^{NEG} c-Kit^{POS} CD135^{NEG} FcγRII/III^{POS} CD34^{POS}), and mo-mac (CD45^{POS} CD64^{POS} CD2^{NEG} Siglec-F^{NEG}) in naïve and tumor-bearing lungs of young and old mice at 5-, 10- and 20-days post-tumor inoculation. Across all experiments, n=3-5 mice were used per group. Data in panels (**A**) and (**B**) are from two independent experiments. Data in panel (**C**) are representative of two independent experiments. In panel (**D**), data from day 20 are representative of three independent experiments; other timepoints are from one independent experiment. Across all panels, data represent mean ± SEM. *P*-values computed by unpaired *t*-test. (AM, alveolar macrophage; mo-mac, monocyte-derived macrophage; lung MP, lung progenitor-like myeloid cell)

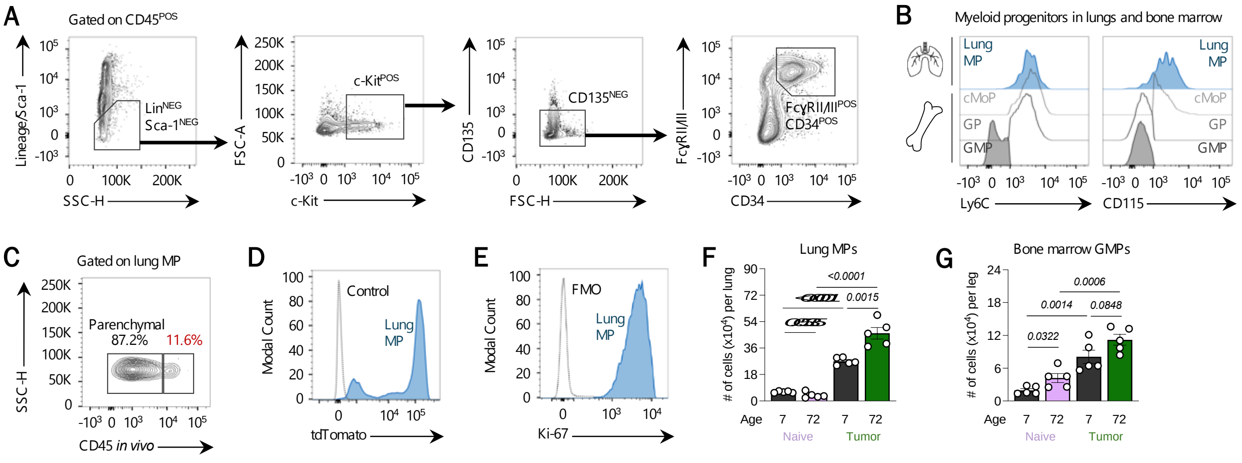

Fig. S3. Expansion of progenitor-like myeloid cells in lung tumors is a local, tumor-specific readout of emergency myelopoiesis. (**A**) Gating strategy for lung MPs (CD45^{POS} Lin^{NEG} Sca-1^{NEG} c-Kit^{POS} CD135^{NEG} FcɣRII/III^{POS} CD34^{POS}) in the lungs of young (7-week-old, 7.KPAR) and old (72-week-old, 72.KPAR) mice. (**B**) Surface expression of Ly6C and CD115 on lung MPs, compared to expression profiles of GMP, GP, and cMoP in bone marrow. (**C**) *In vivo* labeling of intra- and extravascular (parenchymal) immune cells via intravenous administration of APC-conjugated anti-CD45 antibody, separating lung MPs that are in the lung vasculature or are in the lung parenchyma. (**D**) tdTomato signal from lung MPs isolated from the tumor-bearing lungs of old *Ms4a3*^{CRE}-TdT mice, indicating that lung MPs are of bone marrow origin. (**E**) Intracellular staining for Ki-67 in lung MPs. Absolute number of (**F**) lung MPs and (**G**) bone marrow GMPs in the lungs and bone marrow, respectively, of naïve and tumor-bearing young and old mice. In panels (**F**) and (**G**), n=5 mice were used per group and are representative of at least two independent experiments. Across these panels, data represent mean ± SEM. *P*-values were computed by unpaired *t*-test. (Lung MP, lung progenitor-like myeloid cell; GMP, granulocyte-monocyte progenitor; GP, granulocyte progenitor; cMoP, common monocyte progenitor)

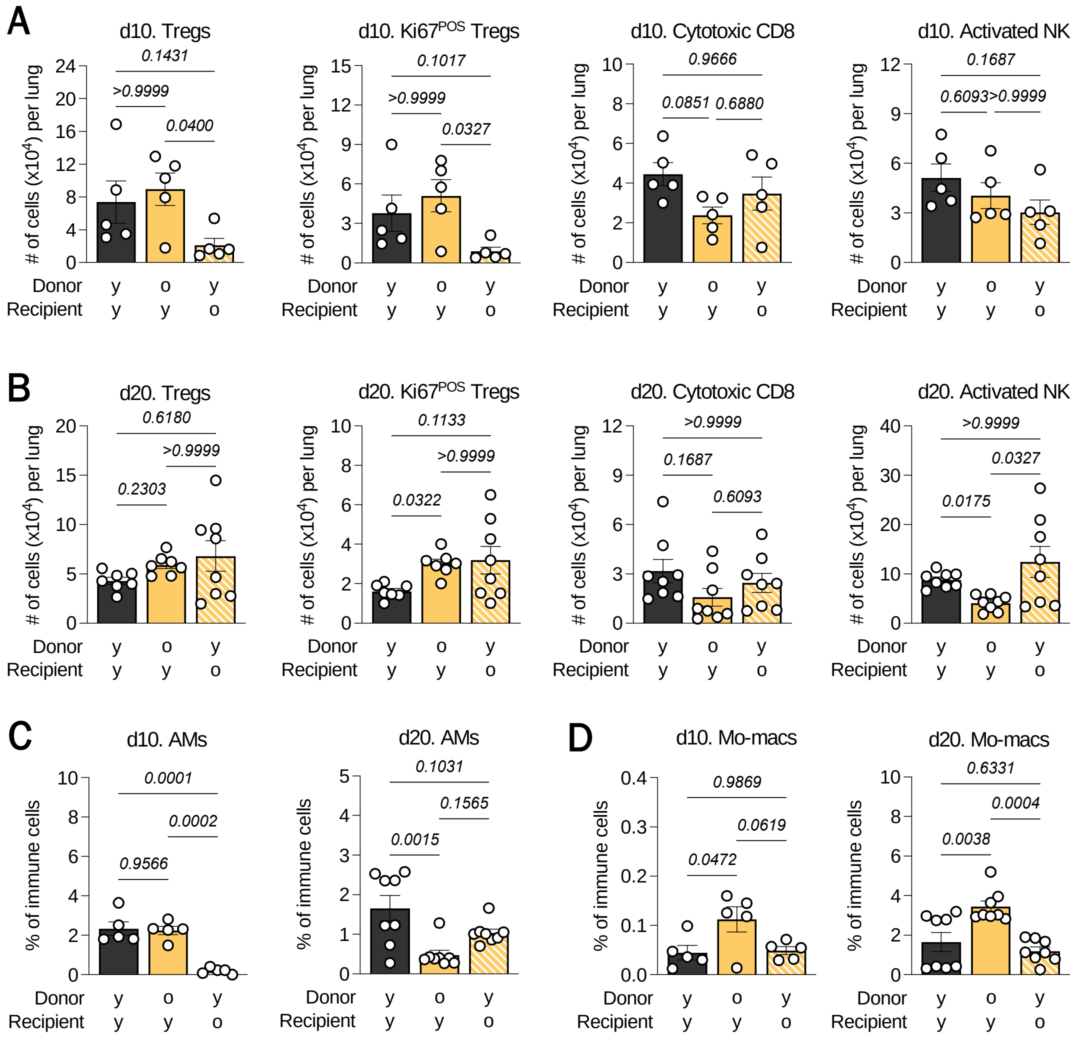

Fig. S4. A dysregulated adaptive immune response underscores age-dependent lung cancer progression. (**A**) Quantification of Tregs ($CD45^{POS}$ $CD3^{POS}$ $CD4^{POS}$ $FoxP3^{POS}$), cycling Tregs ($Ki67^{POS}$ Tregs), cytotoxic CD8 T cells ($CD45^{POS}$ $CD3^{POS}$ $CD8^{POS}$ IFN-γ^{POS} TNF-α^{POS}), and activated NK cells ($CD45^{POS}$ $CD3^{NEG}$ $NKp46^{POS}$ $CD69^{POS}$) in lung tumors of heterochronic bone marrow transplant mice at 10 days post-inoculation. (**B**) Quantification of the lymphoid cell populations shown in (**A**) at 20 days post-inoculation. Frequencies of (**C**) AMs and (**D**) mo-macs in tumor-bearing lungs of chimeric mice at 10 days and 20 days post-inoculation. Data on the day 10 timepoint are from one experiment; data on the day 20 endpoint are from two independent experiments. Across all panels, data represent mean ± SEM. *P*-values were computed by one-way ANOVA. (Treg, regulatory T cell; NK cell, natural killer cell; AM, alveolar macrophage; mo-mac, monocyte-derived macrophage)

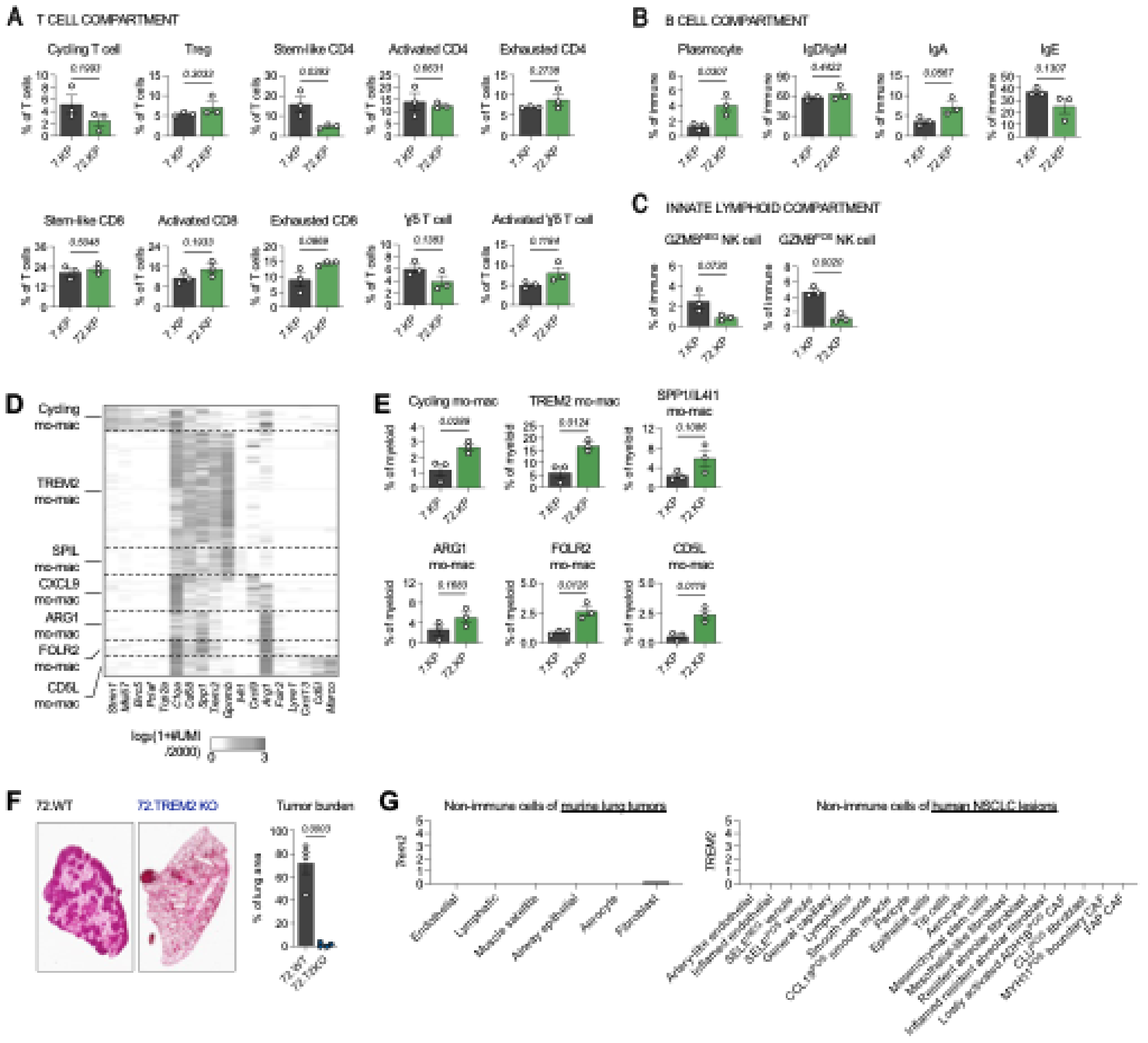

Fig. S5. A single-cell atlas of lung cancer and aging. Immune cell subtypes seen in the single-cell RNA sequencing of sorted immune cells from tumor-bearing lungs of young (7-week-old, 7.wks) and old (72-week-old, 72.wks) mice (n=3 mice per group). Frequency differences between young and old mice of cell subtypes in the (**A**) T cell compartment, (**B**) B cell compartment, and (**C**) innate lymphoid cell compartment. (**D**) Heatmap showing gene expression of highly variable markers (per column) for each single cell (rows) classified as a monocyte-derived macrophage (mo-mac). (**E**) Frequency differences of mo-mac cell subtypes in young and in old tumor-bearing mice. (**F**) Tumor burden in the lungs of old wild-type (WT) and *Trem2* knockout (TREM2 KO) mice (n=4 mice per group). (**G**) (Left) Expression of *Trem2* by non-immune cells in tumor-bearing lungs of mice, and (right) expression of *TREM2* by non-immune cells in human NSCLC lesions. Data shown in (**A**)-(**F**) are one independent experiment. Raw data from panel (**G**, right) were taken from Grout et al., 2022. Data represent mean ± SEM. *P*-values were computed by unpaired student's *t*-test.

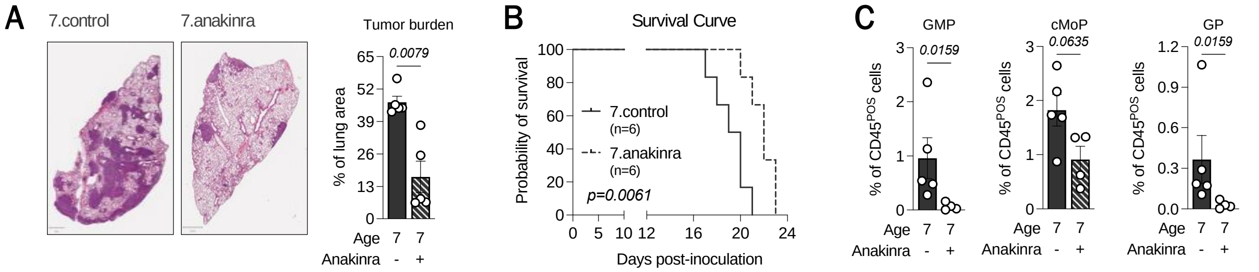

Fig. S6. Anakinra confers a delayed therapeutic effect in young mice. (**A**) Tumor burden in the lungs of young mice that received either control (PBS) or anakinra after 22 days post-inoculation. Scale bar = 1mm. (**B**) Survival curve of control and anakinra-treated tumor-bearing young mice. Solid line: control; Dotted line: anakinra-treated. Difference in median survival of 2.5 days. *P*-value was computed using the Log-rank Mantel-Cox test. (**C**) Frequency of GMPs, cMoPs, and GPs in the bone marrow of control and anakinra-treated young mice at 22 days post tumor-cell inoculation. Data shown in (**A**), (**C**) are representative of two experiments. Data are represented as mean ± SEM. *P*-values were computed by unpaired Mann Whitney *t*-test. (GMP, granulocyte-monocyte progenitor; cMoP, common monocyte progenitor; GP, granulocyte progenitor)

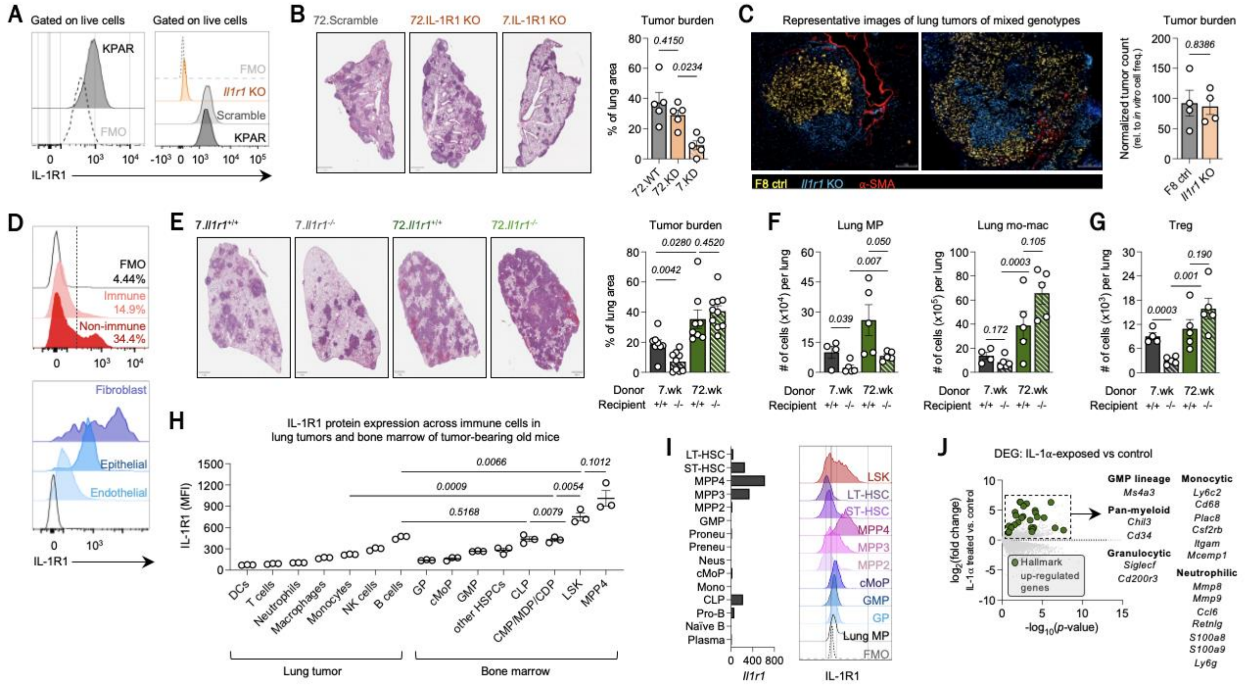

Fig. S7. Hematopoietic response to IL-1α signaling underscores age-enhanced tumor progression. (**A**) Protein expression of IL-1R1 on wild-type/scramble and IL-1R1-deficient KPAR cells. (**B**) Tumor burden in mice challenged with either scramble KPAR cells or *Il1r1* KD KPAR cells at 20 days post-inoculation. Scale bar = 1 mm. (**C**) Representative images of IL-1R1-proficient (yellow) and –deficient (blue) KPAR tumors sharing the same microenvironment in young mice (left) and normalized tumor count measurement of the two genotypes (right). Scale bar = 100 µm. (**D**) Protein expression of IL-1R1 on immune and non-immune cells (top) and specifically fibroblasts, epithelial cells, and endothelial cells (bottom) in the TME of the lungs from tumor-bearing old mice. (**E**) Tumor burden in the lungs of chimeric IL-1R1-proficient and –deficient recipients of young and old donor bone marrow. Scale bar = 1mm. Absolute number of (**F**) lung MPs and mo-macs and (**G**) Tregs in the tumor-bearing lungs of chimeric mice shown in (**E**). (**H**) Protein expression of IL-1R1 by local immune cells and hematopoietic progenitors in bone marrow of tumor-bearing old mice. (**I**) mRNA (left) and protein expression (right) of IL-1R1 by hematopoietic progenitors in bone marrow of naïve mice. (**J**) DEGs between HSPCs exposed to IL-1α and controls. Hallmark genes plotted in green. Data represent mean ± SEM. *P*-values were computed by unpaired *t*-test. (IL-1R1, IL-1 receptor 1; lung MP, progenitor-like myeloid cell; mo-mac, monocyte-derived macrophage; Treg, regulatory T cell; HSPC, hematopoietic stem and progenitor cell)

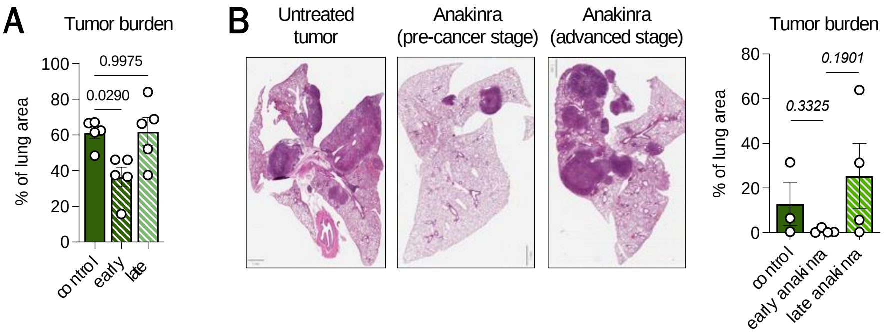

Fig. S8. Blocking IL-1 signaling during tumor development delays cancer progression. (**A**) Tumor burden in the tumor-bearing lungs of control (PBS) mice and mice that either received anakinra immediately following inoculation or 2 weeks post-inoculation. Scale bar = 1 mm. (**B**) Tumor burden in the tumor-bearing lungs of genetically-engineered *Kras*$^{G12D/+}$ *p53*$^{-/-}$ mice that either received control (PBS), anakinra immediately following Cre recombination, or anakinra at 14 weeks post-recombination. Data represent mean ± SEM. *P*-values were computed by unpaired *t*-test (with Welch's correction in (**B**)).

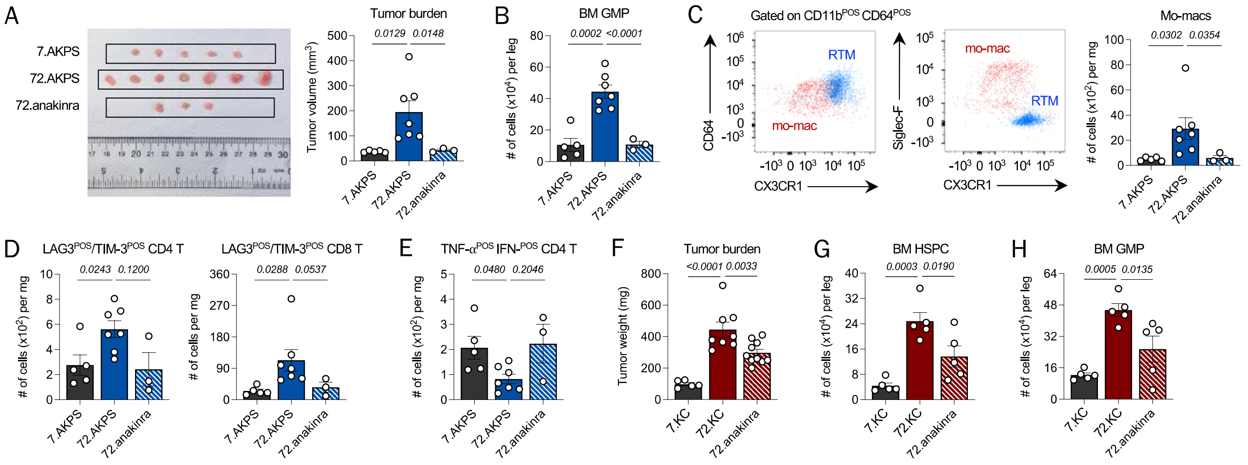

Fig. S9. Blocking IL-1 signaling delays the age-enhanced progression of colorectal and pancreatic cancer. To model colorectal cancer, *Apc*$^{fl/fl}$ *Kras*$^{G12D/+}$ *p53*$^{fl/fl}$ *Smad4*$^{fl/fl}$ (AKPS) tumor cells were orthotopically transplanted into mice via intracecal injection. (**A**) Tumor burden in young and old mice that received control (PBS) and old mice that received anakinra starting at one-week post-inoculation. (**B**) Absolute number of GMPs, GPs, and cMoPs in bone marrow of mice shown in (A). Absolute number of (**C**) mo-macs, (**D**) exhausted CD4 and CD8 T cells, and (**E**) cytotoxic CD4 T cells in AKPS tumors surgically excised from the colon of mice shown in (**A**). To model pancreatic cancer, pancreatic adenocarcinoma cancer cell lines derived from Kras$^{LSL-G12D/+}$; *Ptf1a*$^{CRE/+}$ (KC) mice were orthotopically transplanted into the tail of the pancreas of young and old mice. (**F**) Tumor burden in young and old mice that received control (PBS) and old mice that received anakinra starting at one-week post-inoculation. Absolute number of (**G**) HSPCs and (**H**) GMPs in bone marrow of mice shown in (**F**). In panels (**A**)-(**E**), n=3-7 mice per group were used; in panels (**F**)-(**H**), n=5-10 mice per group were used. Data represent mean ± SEM. *P*-values were computed by unpaired *t*-test or ordinary one-way ANOVA. (HSPC, hematopoietic stem and progenitor cell; GMP, granulocyte-monocyte progenitor; GP, granulocyte progenitor; cMoP, common monocyte progenitor; mo-mac, monocyte-derived macrophage)

Supplemental Tables

Table S1. Age-associated transcriptional differences between myeloid cells in lung tumors of young and old mice by cell type. Differentially expressed genes (DEG) that distinguish differentiated myeloid and myeloid progenitor cell types in lung tumors of young and old mice.

Table S2. Functional markers of maturation for differentiated myeloid cells. Transcriptional expression of known cell surface markers of maturation for differentiated myeloid cells.

Table S3. Transcriptional signature for the monocytic progenitor cell state. Differentially expressed genes (DEG) that define the monocytic progenitors (MonoP) and constitute the IL-1α mRNA program.

References and Notes

1. S. Pilleron, E. Soto-Perez-de-Celis, J. Vignat, J. Ferlay, I. Soerjomataram, F. Bray, D. Sarfati, Estimated global cancer incidence in the oldest adults in 2018 and projections to 2050. *Int. J. Cancer* **148**, 601–608 (2021). doi:10.1002/ijc.33232 Medline

2. C. López-Otín, F. Pietrocola, D. Roiz-Valle, L. Galluzzi, G. Kroemer, Meta-hallmarks of aging and cancer. *Cell Metab.* **35**, 12–35 (2023). doi:10.1016/j.cmet.2022.11.001 Medline

3. Z. Yu, J. Wang, L. Feng, X. Yang, Q. Qi, W. Li, X. Zhang, M. Ge, H. Qin, Association of tumor mutational burden with age in solid tumors. *J. Clin. Oncol.* **38** (15_suppl), e13590–e13590 (2020). doi:10.1200/JCO.2020.38.15_suppl.e13590

4. M. Klutstein, J. Moss, T. Kaplan, H. Cedar, Contribution of epigenetic mechanisms to variation in cancer risk among tissues. *Proc. Natl. Acad. Sci. U.S.A.* **114**, 2230–2234 (2017). doi:10.1073/pnas.1616556114 Medline

5. C. Tomasetti, L. Li, B. Vogelstein, Stem cell divisions, somatic mutations, cancer etiology, and cancer prevention. *Science* **355**, 1330–1334 (2017). doi:10.1126/science.aaf9011 Medline

6. S. Han, P. Georgiev, A. E. Ringel, A. H. Sharpe, M. C. Haigis, Age-associated remodeling of T cell immunity and metabolism. *Cell Metab.* **35**, 36–55 (2023). doi:10.1016/j.cmet.2022.11.005 Medline

7. B. K. Kennedy, S. L. Berger, A. Brunet, J. Campisi, A. M. Cuervo, E. S. Epel, C. Franceschi, G. J. Lithgow, R. I. Morimoto, J. E. Pessin, T. A. Rando, A. Richardson, E. E. Schadt, T. Wyss-Coray, F. Sierra, Geroscience: Linking aging to chronic disease. *Cell* **159**, 709–713 (2014). doi:10.1016/j.cell.2014.10.039 Medline

8. L. Ferrucci, E. Fabbri, Inflammageing: Chronic inflammation in ageing, cardiovascular disease, and frailty. *Nat. Rev. Cardiol.* **15**, 505–522 (2018). doi:10.1038/s41569-018-0064-2 Medline

9. J. L. Schneider, J. H. Rowe, C. Garcia-de-Alba, C. F. Kim, A. H. Sharpe, M. C. Haigis, The aging lung: Physiology, disease, and immunity. *Cell* **184**, 1990–2019 (2021). doi:10.1016/j.cell.2021.03.005 Medline

10. M. R. L. Lichtenstein, R. D. Nipp, A. Muzikansky, K. Goodwin, D. Anderson, R. A. Newcomb, J. F. Gainor, Impact of Age on Outcomes with Immunotherapy in Patients with Non-Small Cell Lung Cancer. *J. Thorac. Oncol.* **14**, 547–552 (2019). doi:10.1016/j.jtho.2018.11.011 Medline

11. Z. Ma, C. Zhu, H. Wang, M. Ji, Y. Huang, X. Wei, J. Zhang, Y. Wang, R. Yin, J. Dai, L. Xu, H. Ma, Z. Hu, G. Jin, M. Zhu, H. Shen, Association between biological aging and lung cancer risk: Cohort study and Mendelian randomization analysis. *iScience* **26**, 106018 (2023). doi:10.1016/j.isci.2023.106018 Medline

12. R. Blanco, I. Maestu, M. G. de la Torre, A. Cassinello, I. Nuñez, A review of the management of elderly patients with non-small-cell lung cancer. *Ann. Oncol.* **26**, 451–463 (2015). doi:10.1093/annonc/mdu268 Medline

13. J. Boumelha, S. de Carné Trécesson, E. K. Law, P. Romero-Clavijo, M. A. Coelho, K. W. Ng, E. Mugarza, C. Moore, S. Rana, D. R. Caswell, M. Murillo, D. C. Hancock, P. P. Argyris, W. L. Brown, C. Durfee, L. K. Larson, R. I. Vogel, A. Suárez-Bonnet, S. L. Priestnall, P. East, S. J. Ross, G. Kassiotis, M. Molina-Arcas, C. Swanton, R. Harris, J. Downward, An Immunogenic Model of KRAS-Mutant Lung Cancer Enables Evaluation of Targeted Therapy and Immunotherapy Combinations. *Cancer Res.* **82**, 3435–3448 (2022). doi:10.1158/0008-5472.CAN-22-0325 Medline

14. M. D. Park, A. Silvin, F. Ginhoux, M. Merad, Macrophages in health and disease. *Cell* **185**, 4259–4279 (2022). doi:10.1016/j.cell.2022.10.007 Medline

15. H. Aegerter, J. Kulikauskaite, S. Crotta, H. Patel, G. Kelly, E. M. Hessel, M. Mack, S. Beinke, A. Wack, Influenza-induced monocyte-derived alveolar macrophages confer prolonged antibacterial protection. *Nat. Immunol.* **21**, 145–157 (2020). doi:10.1038/s41590-019-0568-x Medline

16. B. Machiels, M. Dourcy, X. Xiao, J. Javaux, C. Mesnil, C. Sabatel, D. Desmecht, F. Lallemand, P. Martinive, H. Hammad, M. Guilliams, B. Dewals, A. Vanderplasschen, B. N. Lambrecht, F. Bureau, L. Gillet, A gammaherpesvirus provides protection against allergic asthma by inducing the replacement of resident alveolar macrophages with regulatory monocytes. *Nat. Immunol.* **18**, 1310–1320 (2017). doi:10.1038/ni.3857 Medline

17. Z. Liu, Y. Gu, S. Chakarov, C. Bleriot, I. Kwok, X. Chen, A. Shin, W. Huang, R. J. Dress, C.-A. Dutertre, A. Schlitzer, J. Chen, L. G. Ng, H. Wang, Z. Liu, B. Su, F. Ginhoux, Fate Mapping via Ms4a3-Expression History Traces Monocyte-Derived Cells. *Cell* **178**, 1509–1525.e19 (2019). doi:10.1016/j.cell.2019.08.009 Medline

18. K. Adé, J. S. Coronilla, D. Obino, T. Weinberger, C. Kaiser, S. Mella, C. Chen, L. Katsimpardi, C. Werts, H. Li, P. Dardenne, Y. Lallemand, E. G. Perdiguero, Inflammation drives age-induced loss of tissue resident macrophages. *bioRxiv* (2022), p. 2022.10.02.510513.

19. I. Shchukina, J. Bagaitkar, O. Shpynov, E. Loginicheva, S. Porter, D. A. Mogilenko, E. Wolin, P. Collins, G. Demidov, M. Artomov, K. Zaitsev, S. Sidorov, C. Camell, M. Bambouskova, L. Arthur, A. Swain, A. Panteleeva, A. Dievskii, E. Kurbatsky, P. Tsurinov, R. Chernyatchik, V. D. Dixit, M. Jovanovic, S. A. Stewart, M. J. Daly, S. Dmitriev, E. M. Oltz, M. N. Artyomov, Enhanced epigenetic profiling of classical human monocytes reveals a specific signature of healthy aging in the DNA methylome. *Nat. Aging* **1**, 124–141 (2021). doi:10.1038/s43587-020-00002-6 Medline

20. K. Hashimoto, T. Kouno, T. Ikawa, N. Hayatsu, Y. Miyajima, H. Yabukami, T. Terooatea, T. Sasaki, T. Suzuki, M. Valentine, G. Pascarella, Y. Okazaki, H. Suzuki, J. W. Shin, A. Minoda, I. Taniuchi, H. Okano, Y. Arai, N. Hirose, P. Carninci, Single-cell transcriptomics reveals expansion of cytotoxic CD4 T cells in supercentenarians. *Proc. Natl. Acad. Sci. U.S.A.* **116**, 24242–24251 (2019). doi:10.1073/pnas.1907883116 Medline

21. M. Terekhova, A. Swain, P. Bohacova, E. Aladyeva, L. Arthur, A. Laha, D. A. Mogilenko, S. Burdess, V. Sukhov, D. Kleverov, B. Echalar, P. Tsurinov, R. Chernyatchik, K. Husarcikova, M. N. Artyomov, Single-cell atlas of healthy human blood unveils age-related loss of $NKG2C^{+}GZMB^{-}CD8^{+}$ memory T cells and accumulation of type 2

memory T cells. *Immunity* **56**, 2836–2854.e9 (2023). doi:10.1016/j.immuni.2023.10.013 Medline

22. Y. Zheng, X. Liu, W. Le, L. Xie, H. Li, W. Wen, S. Wang, S. Ma, Z. Huang, J. Ye, W. Shi, Y. Ye, Z. Liu, M. Song, W. Zhang, J. J. Han, J. C. I. Belmonte, C. Xiao, J. Qu, H. Wang, G.-H. Liu, W. Su, A human circulating immune cell landscape in aging and COVID-19. *Protein Cell* **11**, 740–770 (2020). doi:10.1007/s13238-020-00762-2 Medline

23. Y. Cao, Y. Fan, F. Li, Y. Hao, Y. Kong, C. Chen, X. Hao, D. Han, G. Li, Z. Wang, C. Song, J. Han, H. Zeng, Phenotypic and functional alterations of monocyte subsets with aging. *Immun. Ageing* **19**, 63 (2022). doi:10.1186/s12979-022-00321-9 Medline

24. H. M. Sadeghi, J. F. Schnelle, J. K. Thoma, P. Nishanian, J. L. Fahey, Phenotypic and functional characteristics of circulating monocytes of elderly persons. *Exp. Gerontol.* **34**, 959–970 (1999). doi:10.1016/S0531-5565(99)00065-0 Medline

25. S. Trzebanski, J.-S. Kim, N. Larossi, A. Raanan, D. Kancheva, J. Bastos, M. Haddad, A. Solomon, E. Sivan, D. Aizik, J. S. Kralova, M. Gross-Vered, S. Boura-Halfon, T. Lapidot, R. Alon, K. Movahedi, S. Jung, Classical monocyte ontogeny dictates their functions and fates as tissue macrophages. *Immunity* **57**, 1710–1712 (2024). doi:10.1016/j.immuni.2024.06.011 Medline

26. I. Beerman, D. Bhattacharya, S. Zandi, M. Sigvardsson, I. L. Weissman, D. Bryder, D. J. Rossi, Functionally distinct hematopoietic stem cells modulate hematopoietic lineage potential during aging by a mechanism of clonal expansion. *Proc. Natl. Acad. Sci. U.S.A.* **107**, 5465–5470 (2010). doi:10.1073/pnas.1000834107 Medline

27. W. W. Pang, E. A. Price, D. Sahoo, I. Beerman, W. J. Maloney, D. J. Rossi, S. L. Schrier, I. L. Weissman, Human bone marrow hematopoietic stem cells are increased in frequency and myeloid-biased with age. *Proc. Natl. Acad. Sci. U.S.A.* **108**, 20012–20017 (2011). doi:10.1073/pnas.1116110108 Medline

28. I. Beerman, C. Bock, B. S. Garrison, Z. D. Smith, H. Gu, A. Meissner, D. J. Rossi, Proliferation-dependent alterations of the DNA methylation landscape underlie hematopoietic stem cell aging. *Cell Stem Cell* **12**, 413–425 (2013). doi:10.1016/j.stem.2013.01.017 Medline

29. N. M. LaMarche, S. Hegde, M. D. Park, B. B. Maier, L. Troncoso, J. Le Berichel, P. Hamon, M. Belabed, R. Mattiuz, C. Hennequin, T. Chin, A. M. Reid, I. Reyes-Torres, E. Nemeth, R. Zhang, O. C. Olson, D. B. Doroshow, N. C. Rohs, J. E. Gomez, R. Veluswamy, N. Hall, N. Venturini, F. Ginhoux, Z. Liu, M. Buckup, I. Figueiredo, V. Roudko, K. Miyake, H. Karasuyama, E. Gonzalez-Kozlova, S. Gnjatic, E. Passegué, S. Kim-Schulze, B. D. Brown, F. R. Hirsch, B. S. Kim, T. U. Marron, M. Merad, An IL-4 signalling axis in bone marrow drives pro-tumorigenic myelopoiesis. *Nature* **625**, 166–174 (2024). Medline

30. A. M. Leader, J. A. Grout, B. B. Maier, B. Y. Nabet, M. D. Park, A. Tabachnikova, C. Chang, L. Walker, A. Lansky, J. Le Berichel, L. Troncoso, N. Malissen, M. Davila, J. C. Martin, G. Magri, K. Tuballes, Z. Zhao, F. Petralia, R. Samstein, N. R. D'Amore, G. Thurston, A. O. Kamphorst, A. Wolf, R. Flores, P. Wang, S. Müller, I. Mellman, M. B. Beasley, H. Salmon, A. H. Rahman, T. U. Marron, E. Kenigsberg, M. Merad, Single-cell analysis of human non-small cell lung cancer lesions refines tumor classification and

patient stratification. *Cancer Cell* **39**, 1594–1609.e12 (2021). doi:10.1016/j.ccell.2021.10.009 Medline

31. M. Casanova-Acebes, E. Dalla, A. M. Leader, J. LeBerichel, J. Nikolic, B. M. Morales, M. Brown, C. Chang, L. Troncoso, S. T. Chen, A. Sastre-Perona, M. D. Park, A. Tabachnikova, M. Dhainaut, P. Hamon, B. Maier, C. M. Sawai, E. Agulló-Pascual, M. Schober, B. D. Brown, B. Reizis, T. Marron, E. Kenigsberg, C. Moussion, P. Benaroch, J. A. Aguirre-Ghiso, M. Merad, Tissue-resident macrophages provide a pro-tumorigenic niche to early NSCLC cells. *Nature* **595**, 578–584 (2021). doi:10.1038/s41586-021-03651-8 Medline

32. P.-L. Loyher, P. Hamon, M. Laviron, A. Meghraoui-Kheddar, E. Goncalves, Z. Deng, S. Torstensson, N. Bercovici, C. Baudesson de Chanville, B. Combadière, F. Geissmann, A. Savina, C. Combadière, A. Boissonnas, Macrophages of distinct origins contribute to tumor development in the lung. *J. Exp. Med.* **215**, 2536–2553 (2018). doi:10.1084/jem.20180534 Medline

33. M. D. Park, I. Reyes-Torres, J. LeBerichel, P. Hamon, N. M. LaMarche, S. Hegde, M. Belabed, L. Troncoso, J. A. Grout, A. Magen, E. Humblin, A. Nair, M. Molgora, J. Hou, J. H. Newman, A. M. Farkas, A. M. Leader, T. Dawson, D. D'Souza, S. Hamel, A. R. Sanchez-Paulete, B. Maier, N. Bhardwaj, J. C. Martin, A. O. Kamphorst, E. Kenigsberg, M. Casanova-Acebes, A. Horowitz, B. D. Brown, L. F. De Andrade, M. Colonna, T. U. Marron, M. Merad, TREM2 macrophages drive NK cell paucity and dysfunction in lung cancer. *Nat. Immunol.* **24**, 792–801 (2023). doi:10.1038/s41590-023-01475-4 Medline

34. V. Cortez-Retamozo, M. Etzrodt, A. Newton, P. J. Rauch, A. Chudnovskiy, C. Berger, R. J. H. Ryan, Y. Iwamoto, B. Marinelli, R. Gorbatov, R. Forghani, T. I. Novobrantseva, V. Koteliansky, J.-L. Figueiredo, J. W. Chen, D. G. Anderson, M. Nahrendorf, F. K. Swirski, R. Weissleder, M. J. Pittet, Origins of tumor-associated macrophages and neutrophils. *Proc. Natl. Acad. Sci. U.S.A.* **109**, 2491–2496 (2012). doi:10.1073/pnas.1113744109 Medline

35. D. A. G. Barisas, A. U. Kabir, J. Wu, K. Krchma, M. Kim, M. Subramanian, B. H. Zinselmeyer, C. L. Stewart, K. Choi, Tumor-derived interleukin-1α and leukemia inhibitory factor promote extramedullary hematopoiesis. *PLOS Biol.* **21**, e3001746 (2023). doi:10.1371/journal.pbio.3001746 Medline

36. L. Sikkema, C. Ramírez-Suástegui, D. C. Strobl, T. E. Gillett, L. Zappia, E. Madissoon, N. S. Markov, L.-E. Zaragosi, Y. Ji, M. Ansari, M.-J. Arguel, L. Apperloo, M. Banchero, C. Bécavin, M. Berg, E. Chichelnitskiy, M.-I. Chung, A. Collin, A. C. A. Gay, J. Gote-Schniering, B. Hooshiar Kashani, K. Inecik, M. Jain, T. S. Kapellos, T. M. Kole, S. Leroy, C. H. Mayr, A. J. Oliver, M. von Papen, L. Peter, C. J. Taylor, T. Walzthoeni, C. Xu, L. T. Bui, C. De Donno, L. Dony, A. Faiz, M. Guo, A. J. Gutierrez, L. Heumos, N. Huang, I. L. Ibarra, N. D. Jackson, P. Kadur Lakshminarasimha Murthy, M. Lotfollahi, T. Tabib, C. Talavera-López, K. J. Travaglini, A. Wilbrey-Clark, K. B. Worlock, M. Yoshida, M. van den Berge, Y. Bossé, T. J. Desai, O. Eickelberg, N. Kaminski, M. A. Krasnow, R. Lafyatis, M. Z. Nikolic, J. E. Powell, J. Rajagopal, M. Rojas, O. Rozenblatt-Rosen, M. A. Seibold, D. Sheppard, D. P. Shepherd, D. D. Sin, W. Timens, A. M. Tsankov, J. Whitsett, Y. Xu, N. E. Banovich, P. Barbry, T. E. Duong, C. S. Falk, K. B. Meyer, J. A. Kropski, D. Pe'er, H. B. Schiller, P. R. Tata, J. L. Schultze, S. A.

Teichmann, A. V. Misharin, M. C. Nawijn, M. D. Luecken, F. J. Theis, K. B. Meyer, J. A. Kropski, D. Pe'er, H. B. Schiller, P. R. Tata, J. L. Schultze, S. A. Teichmann, A. V. Misharin, M. C. Nawijn, M. D. Luecken, F. J. Theis; Lung Biological Network Consortium, An integrated cell atlas of the lung in health and disease. *Nat. Med.* **29**, 1563–1577 (2023). doi:10.1038/s41591-023-02327-2 Medline

37. N. Mende, H. P. Bastos, A. Santoro, K. T. Mahbubani, V. Ciaurro, E. F. Calderbank, M. Quiroga Londoño, K. Sham, G. Mantica, T. Morishima, E. Mitchell, M. R. Lidonnici, F. Meier-Abt, D. Hayler, L. Jardine, A. Curd, M. Haniffa, G. Ferrari, H. Takizawa, N. K. Wilson, B. Göttgens, K. Saeb-Parsy, M. Frontini, E. Laurenti, Unique molecular and functional features of extramedullary hematopoietic stem and progenitor cell reservoirs in humans. *Blood* **139**, 3387–3401 (2022). doi:10.1182/blood.2021013450 Medline

38. W.-C. Wu, H.-W. Sun, H.-T. Chen, J. Liang, X.-J. Yu, C. Wu, Z. Wang, L. Zheng, Circulating hematopoietic stem and progenitor cells are myeloid-biased in cancer patients. *Proc. Natl. Acad. Sci. U.S.A.* **111**, 4221–4226 (2014). doi:10.1073/pnas.1320753111 Medline

39. E. Lefrançais, G. Ortiz-Muñoz, A. Caudrillier, B. Mallavia, F. Liu, D. M. Sayah, E. E. Thornton, M. B. Headley, T. David, S. R. Coughlin, M. F. Krummel, A. D. Leavitt, E. Passegué, M. R. Looney, The lung is a site of platelet biogenesis and a reservoir for haematopoietic progenitors. *Nature* **544**, 105–109 (2017). doi:10.1038/nature21706 Medline

40. K. G. Anderson, K. Mayer-Barber, H. Sung, L. Beura, B. R. James, J. J. Taylor, L. Qunaj, T. S. Griffith, V. Vezys, D. L. Barber, D. Masopust, Intravascular staining for discrimination of vascular and tissue leukocytes. *Nat. Protoc.* **9**, 209–222 (2014). doi:10.1038/nprot.2014.005 Medline

41. P. Hamon, P.-L. Loyher, C. Baudesson de Chanville, F. Licata, C. Combadière, A. Boissonnas, CX3CR1-dependent endothelial margination modulates Ly6C^{high} monocyte systemic deployment upon inflammation in mice. *Blood* **129**, 1296–1307 (2017). doi:10.1182/blood-2016-08-732164 Medline

42. A. Kaur, M. R. Webster, K. Marchbank, R. Behera, A. Ndoye, C. H. Kugel 3rd, V. M. Dang, J. Appleton, M. P. O'Connell, P. Cheng, A. A. Valiga, R. Morissette, N. B. McDonnell, L. Ferrucci, A. V. Kossenkov, K. Meeth, H.-Y. Tang, X. Yin, W. H. Wood 3rd, E. Lehrmann, K. G. Becker, K. T. Flaherty, D. T. Frederick, J. A. Wargo, Z. A. Cooper, M. T. Tetzlaff, C. Hudgens, K. M. Aird, R. Zhang, X. Xu, Q. Liu, E. Bartlett, G. Karakousis, Z. Eroglu, R. S. Lo, M. Chan, A. M. Menzies, G. V. Long, D. B. Johnson, J. Sosman, B. Schilling, D. Schadendorf, D. W. Speicher, M. Bosenberg, A. Ribas, A. T. Weeraratna, sFRP2 in the aged microenvironment drives melanoma metastasis and therapy resistance. *Nature* **532**, 250–254 (2016). doi:10.1038/nature17392 Medline

43. M. E. Fane, Y. Chhabra, G. M. Alicea, D. A. Maranto, S. M. Douglass, M. R. Webster, V. W. Rebecca, G. E. Marino, F. Almeida, B. L. Ecker, D. J. Zabransky, L. Hüser, T. Beer, H.-Y. Tang, A. Kossenkov, M. Herlyn, D. W. Speicher, W. Xu, X. Xu, E. M. Jaffee, J. A. Aguirre-Ghiso, A. T. Weeraratna, Stromal changes in the aged lung induce an emergence from melanoma dormancy. *Nature* **606**, 396–405 (2022). doi:10.1038/s41586-022-04774-2 Medline

44. Z. Gong, Q. Li, J. Shi, P. Li, L. Hua, L. D. Shultz, G. Ren, Immunosuppressive reprogramming of neutrophils by lung mesenchymal cells promotes breast cancer metastasis. *Sci. Immunol.* **8**, eadd5204 (2023). doi:10.1126/sciimmunol.add5204 Medline

45. W. Kuribayashi, M. Oshima, N. Itokawa, S. Koide, Y. Nakajima-Takagi, M. Yamashita, S. Yamazaki, B. Rahmutulla, F. Miura, T. Ito, A. Kaneda, A. Iwama, Limited rejuvenation of aged hematopoietic stem cells in young bone marrow niche. *J. Exp. Med.* **218**, e20192283 (2021). doi:10.1084/jem.20192283 Medline

46. T. T. Ho, P. V. Dellorusso, E. V. Verovskaya, S. T. Bakker, J. Flach, L. K. Smith, P. B. Ventura, O. M. Lansinger, A. Hérault, S. Y. Zhang, Y.-A. Kang, C. A. Mitchell, S. A. Villeda, E. Passegué, Aged hematopoietic stem cells are refractory to bloodborne systemic rejuvenation interventions. *J. Exp. Med.* **218**, e20210223 (2021). doi:10.1084/jem.20210223 Medline

47. N. Itokawa, M. Oshima, S. Koide, N. Takayama, W. Kuribayashi, Y. Nakajima-Takagi, K. Aoyama, S. Yamazaki, K. Yamaguchi, Y. Furukawa, K. Eto, A. Iwama, Epigenetic traits inscribed in chromatin accessibility in aged hematopoietic stem cells. *Nat. Commun.* **13**, 2691 (2022). doi:10.1038/s41467-022-30440-2 Medline

48. I. Kwok, E. Becht, Y. Xia, M. Ng, Y. C. Teh, L. Tan, M. Evrard, J. L. Y. Li, H. T. N. Tran, Y. Tan, D. Liu, A. Mishra, K. H. Liong, K. Leong, Y. Zhang, A. Olsson, C. K. Mantri, P. Shyamsunder, Z. Liu, C. Piot, C.-A. Dutertre, H. Cheng, S. Bari, N. Ang, S. K. Biswas, H. P. Koeffler, H. L. Tey, A. Larbi, I.-H. Su, B. Lee, A. St John, J. K. Y. Chan, W. Y. K. Hwang, J. Chen, N. Salomonis, S. Z. Chong, H. L. Grimes, B. Liu, A. Hidalgo, E. W. Newell, T. Cheng, F. Ginhoux, L. G. Ng, Combinatorial Single-Cell Analyses of Granulocyte-Monocyte Progenitor Heterogeneity Reveals an Early Uni-potent Neutrophil Progenitor. *Immunity* **53**, 303–318.e5 (2020). doi:10.1016/j.immuni.2020.06.005 Medline

49. P. R. Taylor, D. M. Reid, S. E. M. Heinsbroek, G. D. Brown, S. Gordon, S. Y. C. Wong, Dectin-2 is predominantly myeloid restricted and exhibits unique activation-dependent expression on maturing inflammatory monocytes elicited in vivo. *Eur. J. Immunol.* **35**, 2163–2174 (2005). doi:10.1002/eji.200425785 Medline

50. N. N. Jarjour, E. A. Schwarzkopf, T. R. Bradstreet, I. Shchukina, C.-C. Lin, S. C.-C. Huang, C.-W. Lai, M. E. Cook, R. Taneja, T. S. Stappenbeck, G. J. Randolph, M. N. Artyomov, J. F. Urban Jr., B. T. Edelson, Bhlhe40 mediates tissue-specific control of macrophage proliferation in homeostasis and type 2 immunity. *Nat. Immunol.* **20**, 687–700 (2019). doi:10.1038/s41590-019-0382-5 Medline

51. M. E. Cook, N. N. Jarjour, C.-C. Lin, B. T. Edelson, Transcription Factor Bhlhe40 in Immunity and Autoimmunity. *Trends Immunol.* **41**, 1023–1036 (2020). doi:10.1016/j.it.2020.09.002 Medline

52. J. J. Kotzin, S. P. Spencer, S. J. McCright, D. B. U. Kumar, M. A. Collet, W. K. Mowel, E. N. Elliott, A. Uyar, M. A. Makiya, M. C. Dunagin, C. C. D. Harman, A. T. Virtue, S. Zhu, W. Bailis, J. Stein, C. Hughes, A. Raj, E. J. Wherry, L. A. Goff, A. D. Klion, J. L. Rinn, A. Williams, R. A. Flavell, J. Henao-Mejia, The long non-coding RNA Morrbid regulates Bim and short-lived myeloid cell lifespan. *Nature* **537**, 239–243 (2016). doi:10.1038/nature19346 Medline

53. Z. Cai, F. Aguilera, B. Ramdas, S. V. Daulatabad, R. Srivastava, J. J. Kotzin, M. Carroll, G. Wertheim, A. Williams, S. C. Janga, C. Zhang, J. Henao-Mejia, R. Kapur, Targeting Bim via a lncRNA Morrbid Regulates the Survival of Preleukemic and Leukemic Cells. *Cell Rep.* **31**, 107816 (2020). doi:10.1016/j.celrep.2020.107816 Medline

54. E. Koncina, M. Nurmik, V. I. Pozdeev, C. Gilson, M. Tsenkova, R. Begaj, S. Stang, A. Gaigneaux, C. Weindorfer, F. Rodriguez, M. Schmoetten, E. Klein, J. Karta, V. S. Atanasova, K. Grzyb, P. Ullmann, R. Halder, M. Hengstschläger, J. Graas, V. Augendre, Y. E. Karapetyan, L. Kerger, N. Zuegel, A. Skupin, S. Haan, J. Meiser, H. Dolznig, E. Letellier, IL1R1$^+$ cancer-associated fibroblasts drive tumor development and immunosuppression in colorectal cancer. *Nat. Commun.* **14**, 4251 (2023). doi:10.1038/s41467-023-39953-w Medline

55. D. F. Boyd, E. K. Allen, A. G. Randolph, X. J. Guo, Y. Weng, C. J. Sanders, R. Bajracharya, N. K. Lee, C. S. Guy, P. Vogel, W. Guan, Y. Li, X. Liu, T. Novak, M. M. Newhams, T. P. Fabrizio, N. Wohlgemuth, P. M. Mourani, T. N. Wight, S. Schultz-Cherry, S. A. Cormier, K. Shaw-Saliba, A. Pekosz, R. E. Rothman, K.-F. Chen, Z. Yang, R. J. Webby, N. Zhong, J. C. Crawford, P. G. Thomas; PALISI Pediatric Intensive Care Influenza (PICFLU) Investigators, Exuberant fibroblast activity compromises lung function via ADAMTS4. *Nature* **587**, 466–471 (2020). doi:10.1038/s41586-020-2877-5 Medline

56. J. W. Griffith, L. D. Faustino, V. I. Cottrell, K. Nepal, L. P. Hariri, R. S.-Y. Chiu, M. C. Jones, A. Julé, C. Gabay, A. D. Luster, Regulatory T cell-derived IL-1Ra suppresses the innate response to respiratory viral infection. *Nat. Immunol.* **24**, 2091–2107 (2023). doi:10.1038/s41590-023-01655-2 Medline

57. A. V. Orjalo, D. Bhaumik, B. K. Gengler, G. K. Scott, J. Campisi, Cell surface-bound IL-1alpha is an upstream regulator of the senescence-associated IL-6/IL-8 cytokine network. *Proc. Natl. Acad. Sci. U.S.A.* **106**, 17031–17036 (2009). doi:10.1073/pnas.0905299106 Medline

58. M. I. Suwara, N. J. Green, L. A. Borthwick, J. Mann, K. D. Mayer-Barber, L. Barron, P. A. Corris, S. N. Farrow, T. A. Wynn, A. J. Fisher, D. A. Mann, IL-1α released from damaged epithelial cells is sufficient and essential to trigger inflammatory responses in human lung fibroblasts. *Mucosal Immunol.* **7**, 684–693 (2014). doi:10.1038/mi.2013.87 Medline

59. R.-M. Laberge, Y. Sun, A. V. Orjalo, C. K. Patil, A. Freund, L. Zhou, S. C. Curran, A. R. Davalos, K. A. Wilson-Edell, S. Liu, C. Limbad, M. Demaria, P. Li, G. B. Hubbard, Y. Ikeno, M. Javors, P.-Y. Desprez, C. C. Benz, P. Kapahi, P. S. Nelson, J. Campisi, MTOR regulates the pro-tumorigenic senescence-associated secretory phenotype by promoting IL1A translation. *Nat. Cell Biol.* **17**, 1049–1061 (2015). doi:10.1038/ncb3195 Medline

60. E. T. Osei, J. A. Noordhoek, T. L. Hackett, A. I. R. Spanjer, D. S. Postma, W. Timens, C. A. Brandsma, I. H. Heijink, Interleukin-1α drives the dysfunctional cross-talk of the airway epithelium and lung fibroblasts in COPD. *Eur. Respir. J.* **48**, 359–369 (2016). doi:10.1183/13993003.01911-2015 Medline

61. P. R. Nagareddy, M. Kraakman, S. L. Masters, R. A. Stirzaker, D. J. Gorman, R. W. Grant, D. Dragoljevic, E. S. Hong, A. Abdel-Latif, S. S. Smyth, S. H. Choi, J. Korner, K. E. Bornfeldt, E. A. Fisher, V. D. Dixit, A. R. Tall, I. J. Goldberg, A. J. Murphy, Adipose

tissue macrophages promote myelopoiesis and monocytosis in obesity. *Cell Metab.* **19**, 821–835 (2014). doi:10.1016/j.cmet.2014.03.029 Medline

62. I. Mitroulis, K. Ruppova, B. Wang, L.-S. Chen, M. Grzybek, T. Grinenko, A. Eugster, M. Troullinaki, A. Palladini, I. Kourtzelis, A. Chatzigeorgiou, A. Schlitzer, M. Beyer, L. A. B. Joosten, B. Isermann, M. Lesche, A. Petzold, K. Simons, I. Henry, A. Dahl, J. L. Schultze, B. Wielockx, N. Zamboni, P. Mirtschink, Ü. Coskun, G. Hajishengallis, M. G. Netea, T. Chavakis, Modulation of Myelopoiesis Progenitors Is an Integral Component of Trained Immunity. *Cell* **172**, 147–161.e12 (2018). doi:10.1016/j.cell.2017.11.034 Medline

63. A. Christ, P. Günther, M. A. R. Lauterbach, P. Duewell, D. Biswas, K. Pelka, C. J. Scholz, M. Oosting, K. Haendler, K. Baßler, K. Klee, J. Schulte-Schrepping, T. Ulas, S. J. C. F. M. Moorlag, V. Kumar, M. H. Park, L. A. B. Joosten, L. A. Groh, N. P. Riksen, T. Espevik, A. Schlitzer, Y. Li, M. L. Fitzgerald, M. G. Netea, J. L. Schultze, E. Latz, Western Diet Triggers NLRP3-Dependent Innate Immune Reprogramming. *Cell* **172**, 162–175.e14 (2018). doi:10.1016/j.cell.2017.12.013 Medline

64. E. M. Pietras, C. Mirantes-Barbeito, S. Fong, D. Loeffler, L. V. Kovtonyuk, S. Zhang, R. Lakshminarasimhan, C. P. Chin, J.-M. Techner, B. Will, C. Nerlov, U. Steidl, M. G. Manz, T. Schroeder, E. Passegué, Chronic interleukin-1 exposure drives haematopoietic stem cells towards precocious myeloid differentiation at the expense of self-renewal. *Nat. Cell Biol.* **18**, 607–618 (2016). doi:10.1038/ncb3346 Medline

65. K. C. Higa, A. Goodspeed, J. S. Chavez, M. De Dominici, E. Danis, V. Zaberezhnyy, J. L. Rabe, D. G. Tenen, E. M. Pietras, J. DeGregori, Chronic interleukin-1 exposure triggers selection for Cebpa-knockout multipotent hematopoietic progenitors. *J. Exp. Med.* **218**, e20200560 (2021). doi:10.1084/jem.20200560 Medline

66. X. Li, H. Wang, X. Yu, G. Saha, L. Kalafati, C. Ioannidis, I. Mitroulis, M. G. Netea, T. Chavakis, G. Hajishengallis, Maladaptive innate immune training of myelopoiesis links inflammatory comorbidities. *Cell* **185**, 1709–1727.e18 (2022). doi:10.1016/j.cell.2022.03.043 Medline

67. F. Caiado, L. V. Kovtonyuk, N. G. Gonullu, J. Fullin, S. Boettcher, M. G. Manz, Aging drives Tet2+/- clonal hematopoiesis via IL-1 signaling. *Blood* **141**, 886–903 (2023). doi:10.1182/blood.2022016835 Medline

68. M. DuPage, A. L. Dooley, T. Jacks, Conditional mouse lung cancer models using adenoviral or lentiviral delivery of Cre recombinase. *Nat. Protoc.* **4**, 1064–1072 (2009). doi:10.1038/nprot.2009.95 Medline

69. R. L. Siegel, N. S. Wagle, A. Cercek, R. A. Smith, A. Jemal, Colorectal cancer statistics, 2023. *CA Cancer J. Clin.* **73**, 233–254 (2023). doi:10.3322/caac.21772 Medline

70. L. V. Kovtonyuk, F. Caiado, S. Garcia-Martin, E.-M. Manz, P. Helbling, H. Takizawa, S. Boettcher, F. Al-Shahrour, C. Nombela-Arrieta, E. Slack, M. G. Manz, IL-1 mediates microbiome-induced inflammaging of hematopoietic stem cells in mice. *Blood* **139**, 44–58 (2022). doi:10.1182/blood.2021011570 Medline

71. D. Sun, M. Luo, M. Jeong, B. Rodriguez, Z. Xia, R. Hannah, H. Wang, T. Le, K. F. Faull, R. Chen, H. Gu, C. Bock, A. Meissner, B. Göttgens, G. J. Darlington, W. Li, M. A. Goodell,

Epigenomic profiling of young and aged HSCs reveals concerted changes during aging that reinforce self-renewal. *Cell Stem Cell* **14**, 673–688 (2014). doi:10.1016/j.stem.2014.03.002 Medline

72. M. D. Kessler, A. Damask, S. O'Keeffe, N. Banerjee, D. Li, K. Watanabe, A. Marketta, M. Van Meter, S. Semrau, J. Horowitz, J. Tang, J. A. Kosmicki, V. M. Rajagopal, Y. Zou, Y. Houvras, A. Ghosh, C. Gillies, J. Mbatchou, R. R. White, N. Verweij, J. Bovijn, N. N. Parikshak, M. G. LeBlanc, M. Jones, D. J. Glass, L. A. Lotta, M. N. Cantor, G. S. Atwal, A. E. Locke, M. A. R. Ferreira, R. Deering, C. Paulding, A. R. Shuldiner, G. Thurston, A. A. Ferrando, W. Salerno, J. G. Reid, J. D. Overton, J. Marchini, H. M. Kang, A. Baras, G. R. Abecasis, E. Jorgenson; Regeneron Genetics Center; GHS-RGC DiscovEHR Collaboration, Common and rare variant associations with clonal haematopoiesis phenotypes. *Nature* **612**, 301–309 (2022). doi:10.1038/s41586-022-05448-9 Medline

73. K. A. Oetjen, K. E. Lindblad, M. Goswami, G. Gui, P. K. Dagur, C. Lai, L. W. Dillon, J. P. McCoy, C. S. Hourigan, Human bone marrow assessment by single-cell RNA sequencing, mass cytometry, and flow cytometry. *JCI Insight* **3**, e124928 (2018). doi:10.1172/jci.insight.124928 Medline

74. M. Ainciburu, T. Ezponda, N. Berastegui, A. Alfonso-Pierola, A. Vilas-Zornoza, P. San Martin-Uriz, D. Alignani, J. Lamo-Espinosa, M. San-Julian, T. Jiménez-Solas, F. Lopez, S. Muntion, F. Sanchez-Guijo, A. Molero, J. Montoro, G. Serrano, A. Diaz-Mazkiaran, M. Lasaga, D. Gomez-Cabrero, M. Diez-Campelo, D. Valcarcel, M. Hernaez, J. P. Romero, F. Prosper, Uncovering perturbations in human hematopoiesis associated with healthy aging and myeloid malignancies at single-cell resolution. *eLife* **12**, e79363 (2023). doi:10.7554/eLife.79363 Medline

75. N. Hadad, D. R. Masser, S. Logan, B. Wronowski, C. A. Mangold, N. Clark, L. Otalora, A. Unnikrishnan, M. M. Ford, C. B. Giles, J. D. Wren, A. Richardson, W. E. Sonntag, D. R. Stanford, W. Freeman, Absence of genomic hypomethylation or regulation of cytosine-modifying enzymes with aging in male and female mice. *Epigenetics Chromatin* **9**, 30 (2016). doi:10.1186/s13072-016-0080-6 Medline

76. D. Midic, J. Rinke, F. Perner, V. Müller, A. Hinze, F. Pester, J. Landschulze, J. Ernst, B. Gruhn, G. Matziolis, F. H. Heidel, A. Hochhaus, T. Ernst, Prevalence and dynamics of clonal hematopoiesis caused by leukemia-associated mutations in elderly individuals without hematologic disorders. *Leukemia* **34**, 2198–2205 (2020). doi:10.1038/s41375-020-0869-y Medline

77. C. R. Zhang, E. L. Ostrander, O. Kukhar, C. Mallaney, J. Sun, E. Haussler, H. Celik, W. K. Koh, K. Y. King, P. Gontarz, G. A. Challen, Txnip Enhances Fitness of Dnmt3a-Mutant Hematopoietic Stem Cells via p21. *Blood Cancer Discov.* **3**, 220–239 (2022). doi:10.1158/2643-3230.BCD-21-0132 Medline

78. M. Jeong, D. Sun, M. Luo, Y. Huang, G. A. Challen, B. Rodriguez, X. Zhang, L. Chavez, H. Wang, R. Hannah, S.-B. Kim, L. Yang, M. Ko, R. Chen, B. Göttgens, J.-S. Lee, P. Gunaratne, L. A. Godley, G. J. Darlington, A. Rao, W. Li, M. A. Goodell, Large conserved domains of low DNA methylation maintained by Dnmt3a. *Nat. Genet.* **46**, 17–23 (2014). doi:10.1038/ng.2836 Medline

79. R. Zheng, A. D. Friedman, D. Small, Targeted inhibition of FLT3 overcomes the block to myeloid differentiation in 32Dcl3 cells caused by expression of FLT3/ITD mutations. *Blood* **100**, 4154–4161 (2002). doi:10.1182/blood-2002-03-0936 Medline

80. T. Schroeder, U. Just, Notch signalling via RBP-J promotes myeloid differentiation. *EMBO J.* **19**, 2558–2568 (2000). doi:10.1093/emboj/19.11.2558 Medline

81. Y. Meng, J. Carrelha, R. Drissen, X. Ren, B. Zhang, A. Gambardella, S. Valletta, S. Thongjuea, S. E. Jacobsen, C. Nerlov, Epigenetic programming defines haematopoietic stem cell fate restriction. *Nat. Cell Biol.* **25**, 812–822 (2023). doi:10.1038/s41556-023-01137-5 Medline

82. A. C. Menezes, R. Jones, A. Shrestha, R. Nicholson, A. Leckenby, A. Azevedo, S. Davies, S. Baker, A. F. Gilkes, R. L. Darley, A. Tonks, Increased expression of RUNX3 inhibits normal human myeloid development. *Leukemia* **36**, 1769–1780 (2022). doi:10.1038/s41375-022-01577-2 Medline

83. C. A. Saito-Reis, V. D. Balise, E. M. Pascetti, M. Jiminez, J. M. Gillette, Tetraspanin CD82 regulates $S1PR_1$-mediated hematopoietic stem and progenitor cell mobilization. *Stem Cell Reports* **16**, 2422–2431 (2021). doi:10.1016/j.stemcr.2021.08.009 Medline

84. Z. Hmama, D. Nandan, L. Sly, K. L. Knutson, P. Herrera-Velit, N. E. Reiner, 1alpha,25-dihydroxyvitamin D(3)-induced myeloid cell differentiation is regulated by a vitamin D receptor-phosphatidylinositol 3-kinase signaling complex. *J. Exp. Med.* **190**, 1583–1594 (1999). doi:10.1084/jem.190.11.1583 Medline

85. K. Wakahashi, K. Minagawa, Y. Kawano, H. Kawano, T. Suzuki, S. Ishii, A. Sada, N. Asada, M. Sato, S. Kato, K. Shide, K. Shimoda, T. Matsui, Y. Katayama, Vitamin D receptor-mediated skewed differentiation of macrophages initiates myelofibrosis and subsequent osteosclerosis. *Blood* **133**, 1619–1629 (2019). doi:10.1182/blood-2018-09-876615 Medline

86. Y.-Y. Chen, Y.-F. Liu, Y.-D. Liu, X.-H. Deng, J. Zhou, IRF7 suppresses hematopoietic regeneration under stress via CXCR4. *Stem Cells* **39**, 183–195 (2021). doi:10.1002/stem.3308 Medline

87. O. A. Guryanova, Y. K. Lieu, F. E. Garrett-Bakelman, B. Spitzer, J. L. Glass, K. Shank, A. B. V. Martinez, S. A. Rivera, B. H. Durham, F. Rapaport, M. D. Keller, S. Pandey, L. Bastian, D. Tovbin, A. R. Weinstein, J. Teruya-Feldstein, O. Abdel-Wahab, V. Santini, C. E. Mason, A. M. Melnick, S. Mukherjee, R. L. Levine, Dnmt3a regulates myeloproliferation and liver-specific expansion of hematopoietic stem and progenitor cells. *Leukemia* **30**, 1133–1142 (2016). doi:10.1038/leu.2015.358 Medline

88. P. J. Rauch, J. Gopakumar, A. J. Silver, D. Nachun, H. Ahmad, M. McConkey, T. Nakao, M. Bosse, T. Rentz, N. Vivanco Gonzalez, N. F. Greenwald, E. F. McCaffrey, Z. Khair, M. Gopakumar, K. B. Rodrigues, A. E. Lin, E. Sinha, M. Fefer, D. N. Cohen, A. Vromman, E. Shvartz, G. Sukhova, S. Bendall, M. Angelo, P. Libby, B. L. Ebert, S. Jaiswal, Loss-of-function mutations in Dnmt3a and Tet2 lead to accelerated atherosclerosis and concordant macrophage phenotypes. *Nat. Cardiovasc. Res.* **2**, 805–818 (2023). doi:10.1038/s44161-023-00326-7 Medline

89. E. Alaluf, B. Vokaer, A. Detavernier, A. Azouz, M. Splittgerber, A. Carrette, L. Boon, F. Libert, M. Soares, A. Le Moine, S. Goriely, Heme oxygenase-1 orchestrates the immunosuppressive program of tumor-associated macrophages. *JCI Insight* **5**, e133929 (2020). doi:10.1172/jci.insight.133929 Medline

90. Y.-J. Chen, G.-N. Li, X.-J. Li, L.-X. Wei, M.-J. Fu, Z.-L. Cheng, Z. Yang, G.-Q. Zhu, X.-D. Wang, C. Zhang, J.-Y. Zhang, Y.-P. Sun, H. Saiyin, J. Zhang, W.-R. Liu, W.-W. Zhu, K.-L. Guan, Y. Xiong, Y. Yang, D. Ye, L.-L. Chen, Targeting IRG1 reverses the immunosuppressive function of tumor-associated macrophages and enhances cancer immunotherapy. *Sci. Adv.* **9**, eadg0654 (2023). doi:10.1126/sciadv.adg0654 Medline

91. E. L. Mills, D. G. Ryan, H. A. Prag, D. Dikovskaya, D. Menon, Z. Zaslona, M. P. Jedrychowski, A. S. H. Costa, M. Higgins, E. Hams, J. Szpyt, M. C. Runtsch, M. S. King, J. F. McGouran, R. Fischer, B. M. Kessler, A. F. McGettrick, M. M. Hughes, R. G. Carroll, L. M. Booty, E. V. Knatko, P. J. Meakin, M. L. J. Ashford, L. K. Modis, G. Brunori, D. C. Sévin, P. G. Fallon, S. T. Caldwell, E. R. S. Kunji, E. T. Chouchani, C. Frezza, A. T. Dinkova-Kostova, R. C. Hartley, M. P. Murphy, L. A. O'Neill, Itaconate is an anti-inflammatory metabolite that activates Nrf2 via alkylation of KEAP1. *Nature* **556**, 113–117 (2018). doi:10.1038/nature25986 Medline

92. I. Cobo, T. N. Tanaka, K. Chandra Mangalhara, A. Lana, C. Yeang, C. Han, J. Schlachetzki, J. Challcombe, B. R. Fixsen, M. Sakai, R. Z. Li, H. Fields, M. Mokry, R. G. Tsai, R. Bejar, K. Prange, M. de Winther, G. S. Shadel, C. K. Glass, DNA methyltransferase 3 alpha and TET methylcytosine dioxygenase 2 restrain mitochondrial DNA-mediated interferon signaling in macrophages. *Immunity* **55**, 1386–1401.e10 (2022). doi:10.1016/j.immuni.2022.06.022 Medline

93. P. G. Kim, A. Niroula, V. Shkolnik, M. McConkey, A. E. Lin, M. Słabicki, J. P. Kemp, A. Bick, C. J. Gibson, G. Griffin, A. Sekar, D. J. Brooks, W. J. Wong, D. N. Cohen, M. M. Uddin, W. J. Shin, J. Pirruccello, J. M. Tsai, M. Agrawal, D. P. Kiel, M. L. Bouxsein, J. B. Richards, D. M. Evans, M. N. Wein, J. F. Charles, S. Jaiswal, P. Natarajan, B. L. Ebert, Dnmt3a-mutated clonal hematopoiesis promotes osteoporosis. *J. Exp. Med.* **218**, e20211872 (2021). doi:10.1084/jem.20211872 Medline

94. J. M. SanMiguel, E. Eudy, M. A. Loberg, K. A. Young, J. J. Mistry, K. D. Mujica, L. S. Schwartz, T. M. Stearns, G. A. Challen, J. J. Trowbridge, Distinct Tumor Necrosis Factor Alpha Receptors Dictate Stem Cell Fitness versus Lineage Output in Dnmt3a-Mutant Clonal Hematopoiesis. *Cancer Discov.* **12**, 2763–2773 (2022). doi:10.1158/2159-8290.CD-22-0086 Medline

95. M. Yamashita, E. Passegué, TNF-α Coordinates Hematopoietic Stem Cell Survival and Myeloid Regeneration. *Cell Stem Cell* **25**, 357–372.e7 (2019). doi:10.1016/j.stem.2019.05.019 Medline

96. Lung Cancer Cohort Consortium (LC3), The blood proteome of imminent lung cancer diagnosis. *Nat. Commun.* **14**, 3042 (2023). Medline

97. Z. Dou, K. Ghosh, M. G. Vizioli, J. Zhu, P. Sen, K. J. Wangensteen, J. Simithy, Y. Lan, Y. Lin, Z. Zhou, B. C. Capell, C. Xu, M. Xu, J. E. Kieckhaefer, T. Jiang, M. Shoshkes-Carmel, K. M. A. A. Tanim, G. N. Barber, J. T. Seykora, S. E. Millar, K. H. Kaestner, B.

A. Garcia, P. D. Adams, S. L. Berger, Cytoplasmic chromatin triggers inflammation in senescence and cancer. *Nature* **550**, 402–406 (2017). doi:10.1038/nature24050 Medline

98. H. Luksch, W. A. Stinson, D. J. Platt, W. Qian, G. Kalugotla, C. A. Miner, B. G. Bennion, A. Gerbaulet, A. Rösen-Wolff, J. J. Miner, STING-associated lung disease in mice relies on T cells but not type I interferon. *J. Allergy Clin. Immunol.* **144**, 254–266.e8 (2019). doi:10.1016/j.jaci.2019.01.044 Medline

99. H. Liu, S. Ghosh, T. Vaidya, S. Bammidi, C. Huang, P. Shang, A. P. Nair, O. Chowdhury, N. A. Stepicheva, A. Strizhakova, S. Hose, N. Mitrousis, S. G. Gadde, T. Mb, P. Strassburger, G. Widmer, E. M. Lad, P. E. Fort, J.-A. Sahel, J. S. Zigler Jr., S. Sethu, P. D. Westenskow, A. D. Proia, A. Sodhi, A. Ghosh, D. Feenstra, D. Sinha, Activated cGAS/STING signaling elicits endothelial cell senescence in early diabetic retinopathy. *JCI Insight* **8**, e168945 (2023). doi:10.1172/jci.insight.168945

100. X. Xie, G. Ma, X. Li, J. Zhao, Z. Zhao, J. Zeng, Activation of innate immune cGAS-STING pathway contributes to Alzheimer's pathogenesis in 5×FAD mice. *Nat. Aging* **3**, 202–212 (2023). doi:10.1038/s43587-022-00337-2 Medline

101. K. B. Chiappinelli, P. L. Strissel, A. Desrichard, H. Li, C. Henke, B. Akman, A. Hein, N. S. Rote, L. M. Cope, A. Snyder, V. Makarov, S. Budhu, D. J. Slamon, J. D. Wolchok, D. M. Pardoll, M. W. Beckmann, C. A. Zahnow, T. Merghoub, T. A. Chan, S. B. Baylin, R. Strick, Inhibiting DNA Methylation Causes an Interferon Response in Cancer via dsRNA Including Endogenous Retroviruses. *Cell* **162**, 974–986 (2015). doi:10.1016/j.cell.2015.07.011 Medline

102. D. Roulois, H. Loo Yau, R. Singhania, Y. Wang, A. Danesh, S. Y. Shen, H. Han, G. Liang, P. A. Jones, T. J. Pugh, C. O'Brien, D. D. De Carvalho, DNA-Demethylating Agents Target Colorectal Cancer Cells by Inducing Viral Mimicry by Endogenous Transcripts. *Cell* **162**, 961–973 (2015). doi:10.1016/j.cell.2015.07.056 Medline

103. M. Scheller, A. K. Ludwig, S. Göllner, C. Rohde, S. Krämer, S. Stäble, M. Janssen, J.-A. Müller, L. He, N. Bäumer, C. Arnold, J. Gerß, M. Schönung, C. Thiede, C. Niederwieser, D. Niederwieser, H. Serve, W. E. Berdel, U. Thiem, I. Hemmerling, F. Leuschner, C. Plass, M. Schlesner, J. Zaugg, M. D. Milsom, A. Trumpp, C. Pabst, D. B. Lipka, C. Müller-Tidow, Hotspot DNMT3A mutations in clonal hematopoiesis and acute myeloid leukemia sensitize cells to azacytidine via viral mimicry response. *Nat. Cancer* **2**, 527–544 (2021). doi:10.1038/s43018-021-00213-9 Medline

104. T. Hong, J. Li, L. Guo, M. Cavalier, T. Wang, Y. Dou, A. DeLaFuente, S. Fang, A. Guzman, K. Wohlan, C. Kapadia, C. Rosas, Y. Yang, C. C. Yin, S. Li, M. J. You, X. Cheng, M. A. Goodell, Y. Zhou, Y. Huang, TET2 modulates spatial relocalization of heterochromatin in aged hematopoietic stem and progenitor cells. *Nat. Aging* **3**, 1387–1400 (2023). doi:10.1038/s43587-023-00505-y Medline

105. E. Kuroda, K. Ozasa, B. Temizoz, K. Ohata, C. X. Koo, T. Kanuma, T. Kusakabe, S. Kobari, M. Horie, Y. Morimoto, S. Nakajima, K. Kabashima, S. F. Ziegler, Y. Iwakura, W. Ise, T. Kurosaki, T. Nagatake, J. Kunisawa, N. Takemura, S. Uematsu, M. Hayashi, T. Aoshi, K. Kobiyama, C. Coban, K. J. Ishii, Inhaled Fine Particles Induce Alveolar Macrophage Death and Interleukin-1α Release to Promote Inducible Bronchus-

Associated Lymphoid Tissue Formation. *Immunity* **45**, 1299–1310 (2016). doi:10.1016/j.immuni.2016.11.010 Medline

106. J. J. Tsay, B. G. Wu, I. Sulaiman, K. Gershner, R. Schluger, Y. Li, T.-A. Yie, P. Meyn, E. Olsen, L. Perez, B. Franca, J. Carpenito, T. Iizumi, M. El-Ashmawy, M. Badri, J. T. Morton, N. Shen, L. He, G. Michaud, S. Rafeq, J. L. Bessich, R. L. Smith, H. Sauthoff, K. Felner, R. Pillai, A.-M. Zavitsanou, S. B. Koralov, V. Mezzano, C. A. Loomis, A. L. Moreira, W. Moore, A. Tsirigos, A. Heguy, W. N. Rom, D. H. Sterman, H. I. Pass, J. C. Clemente, H. Li, R. Bonneau, K.-K. Wong, T. Papagiannakopoulos, L. N. Segal, Lower Airway Dysbiosis Affects Lung Cancer Progression. *Cancer Discov.* **11**, 293–307 (2021). doi:10.1158/2159-8290.CD-20-0263 Medline

107. A. E. Tilley, M. S. Walters, R. Shaykhiev, R. G. Crystal, Cilia dysfunction in lung disease. *Annu. Rev. Physiol.* **77**, 379–406 (2015). doi:10.1146/annurev-physiol-021014-071931 Medline

108. P. M. Ridker, J. G. MacFadyen, T. Thuren, B. M. Everett, P. Libby, R. J. Glynn; CANTOS Trial Group, Effect of interleukin-1β inhibition with canakinumab on incident lung cancer in patients with atherosclerosis: Exploratory results from a randomised, double-blind, placebo-controlled trial. *Lancet* **390**, 1833–1842 (2017). doi:10.1016/S0140-6736(17)32247-X Medline

109. C. Garlanda, A. Mantovani, Interleukin-1 in tumor progression, therapy, and prevention. *Cancer Cell* **39**, 1023–1027 (2021). doi:10.1016/j.ccell.2021.04.011 Medline

110. W. Hong, A. Li, Y. Liu, X. Xiao, D. C. Christiani, R. J. Hung, J. McKay, J. Field, C. I. Amos, C. Cheng, Clonal Hematopoiesis Mutations in Patients with Lung Cancer Are Associated with Lung Cancer Risk Factors. *Cancer Res.* **82**, 199–209 (2022). doi:10.1158/0008-5472.CAN-21-1903 Medline

111. S. Jaiswal, P. Fontanillas, J. Flannick, A. Manning, P. V. Grauman, B. G. Mar, R. C. Lindsley, C. H. Mermel, N. Burtt, A. Chavez, J. M. Higgins, V. Moltchanov, F. C. Kuo, M. J. Kluk, B. Henderson, L. Kinnunen, H. A. Koistinen, C. Ladenvall, G. Getz, A. Correa, B. F. Banahan, S. Gabriel, S. Kathiresan, H. M. Stringham, M. I. McCarthy, M. Boehnke, J. Tuomilehto, C. Haiman, L. Groop, G. Atzmon, J. G. Wilson, D. Neuberg, D. Altshuler, B. L. Ebert, Age-related clonal hematopoiesis associated with adverse outcomes. *N. Engl. J. Med.* **371**, 2488–2498 (2014). doi:10.1056/NEJMoa1408617 Medline

112. S. Jaiswal, P. Natarajan, A. J. Silver, C. J. Gibson, A. G. Bick, E. Shvartz, M. McConkey, N. Gupta, S. Gabriel, D. Ardissino, U. Baber, R. Mehran, V. Fuster, J. Danesh, P. Frossard, D. Saleheen, O. Melander, G. K. Sukhova, D. Neuberg, P. Libby, S. Kathiresan, B. L. Ebert, Clonal Hematopoiesis and Risk of Atherosclerotic Cardiovascular Disease. *N. Engl. J. Med.* **377**, 111–121 (2017). doi:10.1056/NEJMoa1701719 Medline

113. R. Bhattacharya, S. M. Zekavat, J. Haessler, M. Fornage, L. Raffield, M. M. Uddin, A. G. Bick, A. Niroula, B. Yu, C. Gibson, G. Griffin, A. C. Morrison, B. M. Psaty, W. T. Longstreth, J. C. Bis, S. S. Rich, J. I. Rotter, R. P. Tracy, A. Correa, S. Seshadri, A. Johnson, J. M. Collins, K. M. Hayden, T. E. Madsen, C. M. Ballantyne, S. Jaiswal, B. L. Ebert, C. Kooperberg, J. E. Manson, E. A. Whitsel, P. Natarajan, A. P. Reiner; NHLBI

Trans-Omics for Precision Medicine Program, Clonal Hematopoiesis Is Associated With Higher Risk of Stroke. *Stroke* **53**, 788–797 (2022). doi:10.1161/STROKEAHA.121.037388 Medline

114. W. J. Wong, C. Emdin, A. G. Bick, S. M. Zekavat, A. Niroula, J. P. Pirruccello, L. Dichtel, G. Griffin, M. M. Uddin, C. J. Gibson, V. Kovalcik, A. E. Lin, M. E. McConkey, A. Vromman, R. S. Sellar, P. G. Kim, M. Agrawal, J. Weinstock, M. T. Long, B. Yu, R. Banerjee, R. C. Nicholls, A. Dennis, M. Kelly, P.-R. Loh, S. McCarroll, E. Boerwinkle, R. S. Vasan, S. Jaiswal, A. D. Johnson, R. T. Chung, K. Corey, D. Levy, C. Ballantyne, B. L. Ebert, P. Natarajan; NHLBI TOPMed Hematology Working Group, Clonal haematopoiesis and risk of chronic liver disease. *Nature* **616**, 747–754 (2023). doi:10.1038/s41586-023-05857-4 Medline

115. C. Vlasschaert, A. J. M. McNaughton, M. Chong, E. K. Cook, W. Hopman, B. Kestenbaum, C. Robinson-Cohen, J. Garland, S. M. Moran, G. Paré, C. M. Clase, M. Tang, A. Levin, R. Holden, M. J. Rauh, M. B. Lanktree, Association of Clonal Hematopoiesis of Indeterminate Potential with Worse Kidney Function and Anemia in Two Cohorts of Patients with Advanced Chronic Kidney Disease, Association of Clonal Hematopoiesis of Indeterminate Potential with Worse Kidney Function and Anemia in Two Cohorts of Patients with Advanced Chronic Kidney Disease. *J. Am. Soc. Nephrol.* **33**, 985–995 (2022). doi:10.1681/ASN.2021060774 Medline

116. A. A. Z. Dawoud, R. D. Gilbert, W. J. Tapper, N. C. P. Cross, Clonal myelopoiesis promotes adverse outcomes in chronic kidney disease. *Leukemia* **36**, 507–515 (2022). doi:10.1038/s41375-021-01382-3 Medline

117. W. B. Ershler, J. A. Stewart, M. P. Hacker, A. L. Moore, B. H. Tindle, B16 murine melanoma and aging: Slower growth and longer survival in old mice. *J. Natl. Cancer Inst.* **72**, 161–164 (1984a). doi:10.1093/jnci/72.1.161 Medline

118. W. B. Ershler, R. L. Gamelli, A. L. Moore, M. P. Hacker, A. J. Blow, Experimental tumors and aging: Local factors that may account for the observed age advantage in the B16 murine melanoma model. *Exp. Gerontol.* **19**, 367–376 (1984b). doi:10.1016/0531-5565(84)90046-9 Medline

119. W. B. Ershler, A. L. Moore, H. Shore, R. L. Gamelli, Transfer of age-associated restrained tumor growth in mice by old-to-young bone marrow transplantation. *Cancer Res.* **44**, 5677–5680 (1984c). Medline

120. D. Zhivaki, S. N. Kennedy, J. Park, F. Boriello, P. Devant, A. Cao, K. M. Bahleda, S. Murphy, C. McCabe, C. L. Evavold, K. L. Chapman, I. Zanoni, O. Ashenberg, R. J. Xavier, J. C. Kagan, Correction of age-associated defects in dendritic cells enables CD4+ T cells to eradicate tumors. *Cell* **187**, 3888–3903.e18 (2024). doi:10.1016/j.cell.2024.05.026

121. A. C. Y. Chen, S. Jaiswal, D. Martinez, C. Yerinde, K. Ji, V. Miranda, M. E. Fung, S. A. Weiss, M. Zschummel, K. Taguchi, C. S. Garris, T. R. Mempel, N. Hacohen, D. R. Sen, The aged tumor microenvironment limits T cell control of cancer. *Nat. Immunol.* **25**, 1033–1045 (2024). doi:10.1038/s41590-024-01828-7 Medline

122. E. G. Shuldiner, S. Karmakar, M. K. Tsai, J. D. Hebert, Y. J. Tang, L. Andrejka, M. Wang, C. R. Detrick, H. Cai, R. Tang, D. A. Petrov, M. M. Winslow, Aging represses lung tumorigenesis and alters tumor suppression, *bioRxiv* (2024)p. 2024.05.28.596319.

123. X. Zhuang, Q. Wang, S. Joost, A. Ferrena, D. T. Humphreys, Z. Li, M. Blum, K. Bastl, S. Ding, Y. Landais, Y. Zhan, Y. Zhao, R. Chaligne, J.-H. Lee, S. E. Carrasco, U. K. Bhanot, R. P. Koche, M. J. Bott, P. Katajisto, Y. M. Soto-Feliciano, T. Pisanic, T. Thomas, D. Zheng, E. S. Wong, T. Tammela, Aging limits stemness and tumorigenesis in the lung by reprogramming iron homeostasis, *bioRxiv* (2024)p. 2024.06.23.600305.

124. M. J. Yousefzadeh, R. R. Flores, Y. Zhu, Z. C. Schmiechen, R. W. Brooks, C. E. Trussoni, Y. Cui, L. Angelini, K.-A. Lee, S. J. McGowan, A. L. Burrack, D. Wang, Q. Dong, A. Lu, T. Sano, R. D. O'Kelly, C. A. McGuckian, J. I. Kato, M. P. Bank, E. A. Wade, S. P. S. Pillai, J. Klug, W. C. Ladiges, C. E. Burd, S. E. Lewis, N. F. LaRusso, N. V. Vo, Y. Wang, E. E. Kelley, J. Huard, I. M. Stromnes, P. D. Robbins, L. J. Niedernhofer, An aged immune system drives senescence and ageing of solid organs. *Nature* **594**, 100–105 (2021). doi:10.1038/s41586-021-03547-7 Medline

125. L. I. Prieto, I. Sturmlechner, S. I. Graves, C. Zhang, N. P. Goplen, E. S. Yi, J. Sun, H. Li, D. J. Baker, Senescent alveolar macrophages promote early-stage lung tumorigenesis. *Cancer Cell* **41**, 1261–1275.e6 (2023). doi:10.1016/j.ccell.2023.05.006 Medline

126. S. Haston, E. Gonzalez-Gualda, S. Morsli, J. Ge, V. Reen, A. Calderwood, I. Moutsopoulos, L. Panousopoulos, P. Deletic, G. Carreno, R. Guiho, S. Manshaei, J. M. Gonzalez-Meljem, H. Y. Lim, D. J. Simpson, J. Birch, H. A. Pallikonda, T. Chandra, D. Macias, G. J. Doherty, D. M. Rassl, R. C. Rintoul, M. Signore, I. Mohorianu, A. N. Akbar, J. Gil, D. Muñoz-Espín, J. P. Martinez-Barbera, Clearance of senescent macrophages ameliorates tumorigenesis in KRAS-driven lung cancer. *Cancer Cell* **41**, 1242–1260.e6 (2023). doi:10.1016/j.ccell.2023.05.004 Medline

127. C. Falcomatà, S. Bärthel, S. A. Widholz, C. Schneeweis, J. J. Montero, A. Toska, J. Mir, T. Kaltenbacher, J. Heetmeyer, J. J. Swietlik, J.-Y. Cheng, B. Teodorescu, O. Reichert, C. Schmitt, K. Grabichler, A. Coluccio, F. Boniolo, C. Veltkamp, M. Zukowska, A. A. Vargas, W. H. Paik, M. Jesinghaus, K. Steiger, R. Maresch, R. Öllinger, T. Ammon, O. Baranov, M. S. Robles, J. Rechenberger, B. Kuster, F. Meissner, M. Reichert, M. Flossdorf, R. Rad, M. Schmidt-Supprian, G. Schneider, D. Saur, Selective multi-kinase inhibition sensitizes mesenchymal pancreatic cancer to immune checkpoint blockade by remodeling the tumor microenvironment. *Nat. Cancer* **3**, 318–336 (2022). doi:10.1038/s43018-021-00326-1 Medline

138. C. Schmitt, D. Saur, S. Bärthel, C. Falcomatà, Syngeneic Mouse Orthotopic Allografts to Model Pancreatic Cancer. *J. Vis. Exp.* (188): (2022). doi:10.3791/64253-v Medline

129. M. Dhainaut, S. A. Rose, G. Akturk, A. Wroblewska, S. R. Nielsen, E. S. Park, M. Buckup, V. Roudko, L. Pia, R. Sweeney, J. Le Berichel, C. M. Wilk, A. Bektesevic, B. H. Lee, N. Bhardwaj, A. H. Rahman, A. Baccarini, S. Gnjatic, D. Pe'er, M. Merad, B. D. Brown, Spatial CRISPR genomics identifies regulators of the tumor microenvironment. *Cell* **185**, 1223–1239.e20 (2022). doi:10.1016/j.cell.2022.02.015 Medline

130. A. Wroblewska, M. Dhainaut, B. Ben-Zvi, S. A. Rose, E. S. Park, E. D. Amir, A. Bektesevic, A. Baccarini, M. Merad, A. H. Rahman, B. D. Brown, Protein Barcodes

Enable High-Dimensional Single-Cell CRISPR Screens. *Cell* **175**, 1141–1155.e16 (2018). doi:10.1016/j.cell.2018.09.022 Medline
